# Improving Inference from Reported Concentrations in Environmental Surveillance by Modeling the Statistical Features of Digital PCR

**DOI:** 10.1021/acsestwater.5c01051

**Published:** 2026-06-10

**Authors:** Adrian Lison, Timothy R. Julian, Tanja Stadler

**Affiliations:** † Department of Biosystems Science and Engineering, ETH Zurich, Basel 4056, Switzerland; ‡ SIB Swiss Institute of Bioinformatics, Lausanne 1015, Switzerland; § Swiss Federal Institute of Aquatic Science and Technology, 28499 Eawag, Dubendorf 8600, Switzerland; ∥ Swiss Tropical and Public Health Institute, Allschwil 4123, Switzerland; ⊥ University of Basel, Basel 4055, Switzerland

**Keywords:** eDNA, wastewater-based epidemiology, dPCR, limit of detection, limit of quantification

## Abstract

Digital polymerase chain reaction (dPCR) is a powerful technique for quantifying gene targets in environmental samples, with applications ranging from species monitoring to wastewater-based epidemiology. Although accurate statistical models for analyzing dPCR measurements exist, these require exact assay parameters and the number of positive and total PCR partitions. In practice, however, many environmental studies and monitoring programs analyze only concentration estimates, assuming normally or log-normally distributed measurements. Such assumptions ignore key statistical features of PCR assays, including concentration-dependent measurement noise and nondetects, leading to biased environmental estimates. In this work, we present a Bayesian model with a dPCR-specific likelihood that can be fitted directly to reported concentrations, while incorporating uncertainty in assay parameters through interpretable priors. Using real-world case studies of free-eDNA decay in seawater and pathogen transmission from wastewater, we show that our approach yields similar estimates as a fully informed model with partition counts, while avoiding biases from normal or log-normal approximations. This enables accurate inference from dPCR measurements even when partition count data and assay parameters are unavailable. The method is implemented in the R packages “dPCRfit” for regression analyses and “EpiSewer” for wastewater surveillance.

## Graphical abstract

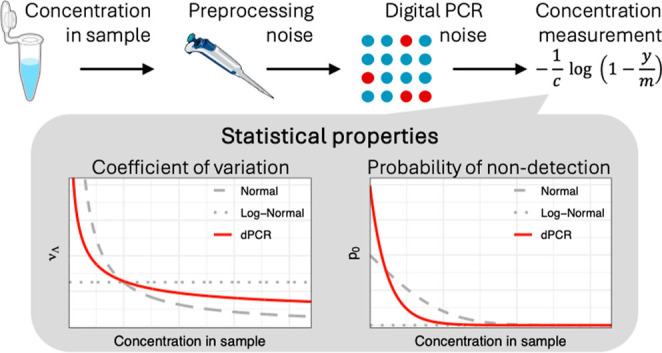

## 1 Introduction

The quantification of gene concentrations from environmental samples has become an important technique in environmental science and engineering, with diverse applications in ecology, conservation, and public health. For example, the collection and analysis of environmental DNA (eDNA), i.e., genetic material shed into the environment by living organisms, enables the detailed monitoring of species abundance and population dynamics.^1,2^ By quantifying the abundance of a specific target gene in samples taken at different locations or times, insights into the occurrence, distribution, movements, and interactions of species can be gained.^3,4^ Similarly, in wastewater-based epidemiology, genetic material from pathogens shed by infected individuals into wastewater is sampled at municipal treatment plants to track infection dynamics in human populations without reliance on clinical testing.^5,6^ This has enabled the cost-effective monitoring of community-level pathogen spread^7–12^ and the estimation of epidemiological parameters, such as the effective reproduction number.^13^

The most widespread techniques for quantifying genetic targets in a sample are quantitative PCR (qPCR) and digital PCR (dPCR). While qPCR quantification relies on a comparison of replication cycle thresholds with a standard curve established from reference samples, dPCR works by partitioning a sample into thousands of independent nanoscale chambers or droplets before PCR amplification.^14^ Using the ratio of positive partitions to the total number of partitions, the concentration in the sample can be estimated.^15^ Due to its independence from standard curves, greater robustness and repeatability, improved handling of inhibition, and increased potential for multiplexing, dPCR is becoming an increasingly popular method for quantifying molecular targets.^16–19^

When statistically analyzing dPCR measurements from multiple environmental samples, a well-established approach is to model the observed number of positive partitions as binomially distributed, conditional on the total valid number of partitions and a concentration-dependent success probability.^15^ This binomial model has been used in both frequentist and Bayesian analyses of dPCR data,^20,21^ and is justified under the assumption that molecules are randomly distributed across independent partitions according to a Poisson process. However, applying the binomial model to dPCR measurements requires several inputs, including the numbers of positive and total valid partitions, the partition volume, the dilution factor, and any changes in concentration due to extraction or added reagents. Although detailed reporting of this information has been recommended,^22^ environmental studies often provide only the maximum-likelihood estimate of concentration (e.g., in gene copies per mL), derived from partition counts using standard software. While this simplifies reporting and facilitates comparison with other quantification methods such as qPCR, it limits the application of binomial modeling because the underlying partition-level data are usually unavailable. This limitation also affects open data from environmental monitoring programs. For instance, wastewater surveillance data published on many countries’ public wastewater dashboards rarely include detailed assay parameters,^23^ hindering the modeling of dPCR partition counts. Instead, many studies directly analyze the reported concentration estimates, typically modeling them as normally^24–30^ or log-normally^9,31–34^ distributed. Although simple to apply, these approaches overlook well-known statistical properties of digital and quantitative PCR.^35,36^ For example, they are unable to capture nonlinear increases in relative measurement error and cannot properly handle nondetects (i.e., zero measurements), which are usually removed or imputed. These issues can bias inference about how concentrations relate to environmental factors or change over time, particularly when analyzing dPCR data with limited sample size and low concentrations, such as estimating species prevalence from a few sampled sites or monitoring pathogen abundance from weekly wastewater samples.

To support reliable inference from dPCR data without complete assay information, such as when analyzing publicly reported environmental data, we here present a statistical model that directly analyzes reported concentration estimates while accounting for the statistical properties of dPCR. Building on established theory of partitioning statistics in dPCR, we propose an approximate likelihood function for reported concentrations that can be applied in regression analyses and more complex modeling of environmental data. This likelihood accounts for the coefficient of variation and the probability of nondetection in dPCR, and adjusts for noise introduced by extraction, preprocessing, and other sources of variation, which can be substantial in the case of environmental samples. When used in a Bayesian framework, the model can also incorporate partial knowledge about assay characteristics and laboratory parameters through suitable priors. We use simulated dPCR data and real-world measurements of (i) marine species free-eDNA decay in seawater and (ii) viral infection dynamics from wastewater to assess the consistency of estimates from our model with those from binomial modeling of positive partition counts.

## 2 Materials and Methods

### 2.1 Partitioning Statistics in Digital PCR

In digital PCR, the goal is to quantify the sample concentration *c*, i. e. the expected number of target molecules per unit volume in the analyzed sample. After extraction and preprocessing, a portion of the resulting reaction mixture is split uniformly into *m* independent partitions, where *m* is often in the order of tens of thousands. The number of target molecules in each partition is assumed to follow a Poisson process,^37^ i. e. partition *q* ∈ {1, ..., *m*} will contain *N*_q_ ∼Pois­(λ) molecules, where λ = *cvs* is the expected number of molecules per partition, which depends on the partition volume *v* as well as a scaling factor *s* that expresses any change in concentration between the original sample and the reaction mixture (e.g., due to extraction, adding of reagents, dilution, and suboptimal recovery efficiency).^38^ We summarize these two factors in a “conversion factor” κ = vs. During PCR, a fluorescent signal is generated if one or several gene copies are present in a partition, leading to a binary result (positive or negative) for each partition, omitting partitions that are deemed not valid. Since the reaction in each partition is regarded as independent, the total number of positive partitions *Y* is binomially distributed with

1 Y∼Binom(m,1−exp(−λ))

where 1 – exp­(−λ) is the probability for at least one gene copy being present in a partition. It is furthermore common to run multiple technical replicates *i* ∈ {1, ..., *n*} of the same sample. The resulting pooled partition counts can be used to compute the maximum likelihood estimate of the concentration *c*, i. e.

2 $$ĉ=-\frac{1}{\kappa }\log\left(1-\frac{\sum_{i=1}^{n}y_{i}}{\sum_{i=1}^{n}m_{i}}\right)$$

where *y*_*i*_ and *m*_*i*_ are the positive and total valid partition counts for replicate *i*, respectively (note that log­(*x*) denotes the natural logarithm of *x*).^15^ In environmental surveillance, studies often report only this maximum likelihood estimate ĉ rather than the positive and total valid partition counts. Moreover, replicate measurements are sometimes also combined using the arithmetic mean of individual maximum likelihood estimates, which gives a concentration similar but not identical to the pooled maximum likelihood estimate (see Supporting Information, Section A.2 for a comparison of both approaches). When partition count data is not available, binomial modeling according to eq 1 is difficult if not impossible, as it would require a back-calculation of the partition counts and exact knowledge of the assay parameters (see Supporting Information, Section F.2). An alternative in such settings is to directly analyze the reported concentration values ĉ , while accounting for their dependence on the partitioning statistics of dPCR, which we describe in the following.

### 2.2 Coefficient of Variation

Since the reported concentration ĉ depends on the binomially distributed positive partition count, it can itself be seen as a random variable Ĉ . Under sufficient dilution in the assay, where the probability of all partitions being positive is almost zero, Ĉ has an asymptotic variance of

3 $$Var\left[Ĉ\right]=\frac{1}{\kappa^{2}\sum_{i=1}^{n}m_{i}}\left(\exp\left(\lambda \right)-1\right)$$

where, as before, λ is the expected number of molecules per partition (see Supporting Information, Section B.1 for derivation). To account for extraction and preprocessing noise and other sources of variation, we apply a random effects model similar to earlier work,^20^ which describes the concentration before partitioning as a random variable *C*_pre_ = *cs*η, where η is a multiplicative noise term with mean 1 and coefficient of variation ν_pre_ > 0. After partitioning, the expected number of molecules per partition is then also a random variable Θ = *C*_pre_ *v*. As we show in Supporting Information, Section B.2, the asymptotic variance of Ĉ under this extended model is

4 $$Var\left[Ĉ\right]=c^{2}\nu_{pre}^{2}+\frac{1}{\kappa^{2}\sum_{i=1}^{n}m_{i}}\left(E\left[\exp\left(\Theta \right)\right]-1\right)$$

where *E*[exp­(Θ)] can be computed from the moment-generating function (MGF) of Θ. For example, when η is gamma-distributed, we obtain

5 $$E\left[\exp\left(\Theta \right)\right]={\left(1-\lambda \nu_{pre}^{2}\right)}^{-\frac{1}{\nu_{pre}^{2}}}$$

We also provide approximations for the case of log-normally distributed noise or if only the mean and variance of *C*_pre_ are known (see Supporting Information, Section B.2).

Using the fact that Ĉ is an asymptotically unbiased estimator of *c* ^37^ (see Supporting Information, Section A.1 for a discussion of the validity for finite partition numbers), we obtain the coefficient of variation (CV) of the measurements as

6 $$\nu_{Ĉ}\left(c\right)=\sqrt{\nu_{pre}^{2}+\frac{1}{c^{2}\kappa^{2}\sum_{i=1}^{n}m_{i}}\left(E\left[\exp\left(\Theta \right)\right]-1\right)}$$

This highlights that the CV of Ĉ is concentration-dependent, which, for instance, introduces heteroskedasticity in linear regression even if measurements are log-transformed.^39^

### 2.3 Probability of Nondetection

A dPCR run can have zero positive partitions even when the target molecule is present. If this happens for all technical replicates, the target is not detected, and the maximum likelihood estimate for the concentration will be ĉ = 0. Absent pre-PCR variation, the probability for nondetection follows directly from the binomial model in eq 1, i. e.

7 $$p_{zero}\left(c\right)=\mathrm{Pr}\left(Ĉ=0|c\right)=\prod_{i=1}^{n}\exp\left(-c\kappa m_{i}\right)=\exp\left(-\lambda \sum_{i=1}^{n}m_{i}\right)$$

This is closely related to the Limit of Detection (LoD) of an assay,^40^ defined as the lowest concentration *c*_LOD_ at which the target will be detected with sufficient probability (see Supporting Information, Section C.1 for further discussion). To account for pre-PCR variation as in Section 2.2, we compute the marginal probability of nondetection, i. e.

8 $$p_{zero}\left(c\right)=E_{\Theta }\left[\mathrm{Pr}\left(Ĉ=0|\Theta \right)\right]=E\left[\exp\left(-\Theta \sum_{i=1}^{n}m_{i}\right)\right]$$

Similar to eq 4, the expectation $E\left[\exp\left(-\Theta \sum_{i=1}^{n}m_{i}\right)\right]$ can be obtained using the MGF of Θ, and we provide solutions for gamma or log-normally distributed pre-PCR noise (see Supporting Information, Section C.2).

### 2.4 Likelihood Function

Based on the concentration-dependent noise and probability of nondetection in digital PCR as derived above, we propose to model dPCR measurements ĉ via a hurdle model, in which concentrations can be measured as zero with probability *p*_zero_(*c*) and otherwise follow a positive continuous probability distribution with density function *f*_PCR_ for nonzero measurements. To adjust for the conditioning on a nonzero outcome, *f*_PCR_ should have a conditional mean

9 $$E\left[Ĉ|Ĉ>0\right]=\frac{c}{1-p_{zero}\left(c\right)}$$

and a conditional coefficient of variation

10 $$\nu_{Ĉ|Ĉ>0}\left(c\right)=\sqrt{\nu_{Ĉ}{\left(c\right)}^{2}\left(1-p_{zero}\left(c\right)\right)-p_{zero}\left(c\right)}$$

see Supporting Information, Section F.1 for details. We note that while ĉ is a real number, its distribution is discrete due to the underlying count nature of the digital PCR. However, as implied by eq 2, the support of this distribution depends both on the conversion factor κ and the number of valid partitions *m*. When these parameters are not reported, the true support is unknown, and we thus model ĉ as continuously distributed. Informed by a simulation-based comparison of different continuous distributions (see Supporting Information, Section F.3), we here chose a gamma density for *f*_PCR_.

Overall, this gives us an approximate likelihood for a dPCR measurement ĉ in the form of a zero-augmented Gamma distribution, i. e.

$$L_{Ĉ}\left(ĉ|c\right)=\{\begin{array}{ll} p_{zero}\left(c\right), & \text{if},ĉ=0\\ \left(1-p_{zero}\left(c\right)\right)p_{Gamma}\left(ĉ;\alpha =\frac{1}{\nu_{Ĉ|Ĉ>0}{\left(c\right)}^{2}},\beta =\frac{1-p_{zero}\left(c\right)}{c\;\nu_{Ĉ|Ĉ>0}{\left(c\right)}^{2}}\right) & otherwise \end{array}$$ 11

where *p*_Gamma_ is the gamma probability density of nonzero concentrations, with shape α and rate β parametrized to give a mean of $\frac{c}{1-p_{zero}\left(c\right)}$ and a coefficient of variation of $\nu_{Ĉ|Ĉ>0}\left(c\right)$ .

We note that a gamma density approximates the true discrete distribution well at larger concentrations, but has limited accuracy at low concentrations, when the probability mass is concentrated on a small set of possible reported concentration values. The approximation accuracy and its implications for inference are further discussed in Section 3.1 and Supporting Information, Section F.4. The identifiability of *c* and κ under this likelihood is demonstrated in Supporting Information, Section F.5.

### 2.5 Inference from dPCR Measurements

We use Bayesian inference with the likelihood defined in eq 11 to model dPCR measurements when the partition counts of an assay are unknown. For this, we require priors for the total number of valid partitions, the conversion factor, and the pre-PCR variation of the assay. To obtain the total number of valid partitions $\sum_{i=1}^{n}m_{i}$ , we model the individual numbers of valid partitions for each technical replicate *i* as *m*_*i*_ = *m*_max_(1 – δ_*i*_), where *m*_max_ is the maximum number of partitions supported by the dPCR system or chip and δ_*i*_ the relative partition loss for replicate *i*. This specification allows us to account for variation in partition loss between technical replicates. We further assume that practically relevant values of *m*_*i*_ are large enough to justify a continuous approximation, allowing efficient estimation via Hamiltonian Monte Carlo (HMC) sampling. Specifically, we place a truncated normal prior on *m*_max_ and a scaled logit-normal prior on δ_*i*_ (see Supporting Information, Section G.1 for details). This prior can easily incorporate additional knowledge, for example, the maximum number of partitions for a dPCR chip is often reported by the manufacturer. To express uncertainty about extraction and preprocessing, we place truncated normal priors on the conversion factor κ and the pre-PCR coefficient of variation ν_pre_.

Given these priors and a set of dPCR measurements ${ĉ}_{j\in \left\{1,...,k\right\}}$ from *k* samples, we can now use the dPCR-specific likelihood $P\left({ĉ}_{j\in \left\{1,...,k\right\}}|c_{j\in \left\{1,...,k\right\}}\right)=\prod_{j=1}^{k}L_{Ĉ}\left({ĉ}_{j}|c_{j}\right)$ to estimate the underlying concentrations *c*_*j*∈{1,...,*k*}_ via Bayesian inference. For example, to fit a concentration model *c*_*j*_ = *f*(*j*, θ) with parameters θ and corresponding prior *P*(θ), we can sample from the posterior distribution P(c,θ|ĉ) via Markov Chain Monte Carlo (MCMC). In this study, we used a generalized linear model (GLM) framework for *f*(*j*, θ), allowing us to estimate the linear association of covariates with the target concentration under a logarithmic link function (Supporting Information, Sections H.3 and I.1). Moreover, as an example of a domain-specific model, we also implemented a model of pathogen concentrations in municipal wastewater for *f*(*j*, θ). Both models are implemented in the probabilistic programming language “stan”^41^ and can be fitted through an interface in the R programming language (see Supporting Information, Section G.2 for details on the estimation). Dedicated R packages for the GLM (“dPCRfit”) and for the wastewater model (“EpiSewer”) are available as open-source software.^42,43^

### 2.6 Model Validation

To validate our approximations for the CV and the probability of nondetection, we compared our theoretical predictions to simulated concentrations estimates under different types of pre-PCR noise. Concentration estimates were computed based on simulated partition counts in a dPCR assay according to eq 1. The coefficient of variation was empirically estimated from 10 000 simulated concentration estimates by dividing the sample standard deviation by the sample mean. The probability of nondetection was empirically estimated by the proportion of zero measurements to the total number of simulated measurements. Further details are provided in Supporting Information, Section D.

To validate our proposed likelihood, we compared the probabilities for reported concentrations implied by eq 11 with those from a binomial model (Supporting Information, Section F.4). For our continuous approximation, we computed the probabilities over bins corresponding to the discrete concentration estimates that can be obtained under eq 2. The bins were defined using midpoints between adjacent attainable concentration estimates. We computed the total absolute error in bins between the continuous dPCR likelihood and the gold-standard binomial model across a range of target concentrations (0.1–100 gc per mL) and for different strengths of pre-PCR noise, assuming a scaling factor of *s* = 30, a partition volume of *v* = 0.519 nL, and *m* = 25 000 partitions per measurement.

To assess the accuracy of estimates under the approximate dPCR likelihood, we used numerical integration to compute posterior distributions for *c* across a range of true target concentrations (0.1–100 gc per mL), assuming a uniform prior $c∼U_{\left[0,10000\right]}$ and the same assay parameters as above. To assess potential bias of our approximation, we computed the deviation of the posterior mean from the true concentration as the number of replicate measurements *n* grows large (Supporting Information, Section H.1). Finally, we tested whether our model can infer a functional relationship for *c* using multiple samples with the same (Supporting Information, Section H.2) or different (Supporting Information, Section H.3) target concentrations. For the latter, we simulated measurements from 10 different samples, whose target concentrations follow a log–linear model, i. e. log­(*c*_*i*_) = 1 + β*x*_*i*_ with *x*_*i*_ ∈ {0, 1, ..., 9}. We fitted a GLM with our dPCR-specific likelihood to the reported concentration values to estimate the slope β. We compared the estimated and true slope across a range of β values (−0.5 and 0.5 in steps of 0.025). For comparison, we also fitted a normal and log-normal likelihood to the measurements, and a binomial model to the corresponding partition counts, assuming knowledge of all assay parameters (see Supporting Information, Sections H.2 and H.3 for details).

### 2.7 Application to eDNA-Based Biomonitoring

We conducted a reanalysis of data from an aquarium study by Scriver et al.,^26^ which tested a direct droplet dPCR assay for eDNA-based marine species monitoring. In the study, the abundance of free-eDNA of the brown bryozoan *Bugula neritina* in aquarium seawater was measured by concentrations of the Cytochrome *c* oxidase subunit 1 (COI) gene at different points in time up to 72 h after removal of the organism from the aquarium. The speed of decline in detectable gene concentrations was then estimated using an exponential decay model. We here applied a GLM with logarithmic link using the “dPCRfit” package to model the exponential decay in free-eDNA, measured in terms of the half-life period. In the original analysis, the authors used the averages of measurements across three tanks with low, medium, and high initial species biomass. Here, we instead applied our GLM to the individual measurements, stratified across tanks. We compared the consistency of eDNA half-life estimates between tanks and with the original result by Scriver et al. when using a dPCR-specific likelihood for the reported concentrations with broad priors for assay parameters (see Supporting Information, Section I for details). For comparison, we also fitted a normal and log-normal likelihood to the measurements, and a binomial likelihood for the partition counts, using the exact assay parameters.

### 2.8 Application to Wastewater-Based Epidemiology

We combined our dPCR-specific likelihood function with a wastewater model implemented in the R package “EpiSewer”^43^ to analyze real-world duplicate dPCR measurements of Influenza A virus from the wastewater treatment plant of Zurich, Switzerland, during the 2022/23, 2023/24, and 2024/25 Influenza seasons. We used discretized estimates from the literature to characterize the generation time distribution (shifted gamma distribution, mean = 2.6, sd = 1.5 ^44,45^ ) and the shedding load distribution since time of infection (gamma distribution, mean = 2.49, sd = 1 ^46^) of Influenza A virus. Based on a comparison with confirmed case numbers, we assumed an average total shedding load per case of 1 × 10^11^ gc.

We applied the model to longitudinal wastewater measurements to estimate the viral load in wastewater and the effective reproduction number *R*_*t*_ over time. The daily viral load in the catchment (in gene copies, gc) was modeled as a latent variable and divided by the measured daily wastewater flow (in ml) at the treatment plant to model the concentration in the sample (in gc/ml). This approach allowed us to account for the varying dilution of genetic material in the wastewater. The model was fitted to the reported sample concentrations from dPCR using a (i) normal, (ii) log-normal, and (iii) dPCR-specific likelihood (assuming broad priors for assay parameters, see Supporting Information, Section J) and, as a gold standard, directly to the underlying partition counts using a binomial likelihood.

## 3 Results

### 3.1 Validation

We compared our theoretical predictions for the coefficient of variation (CV) of reported concentrations and the probability of nondetection with empirical estimates on simulated data and found them to match closely across different levels of pre-PCR noise (Figure 1). Both the CV and probability of nondetection become approximately constant at high concentrations but increase exponentially as the concentration approaches zero. Importantly, the CV also increases when concentrations become extremely high, such that the share of positive partitions approaches one (Figure S6).^35^ Moreover, both the CV and probability of nondetection decrease with the number of partitions (assuming a constant volume per partition) and the number of replicates (Figure S8). For example, a doubling of the number of replicates has the same effect on the probability of nondetection as a doubling of the concentration. Apart from extremely high concentrations, we found that the CV is highly similar under log-normally and gamma-distributed pre-PCR noise and can be well approximated using a Taylor series expansion (Figures S5 and S6). In contrast, the nondetection probability curve differed significantly under log-normal vs gamma-distributed pre-PCR noise (Figure S10), with gamma-distributed noise leading to higher nondetection probabilities.

**Figure 1:**
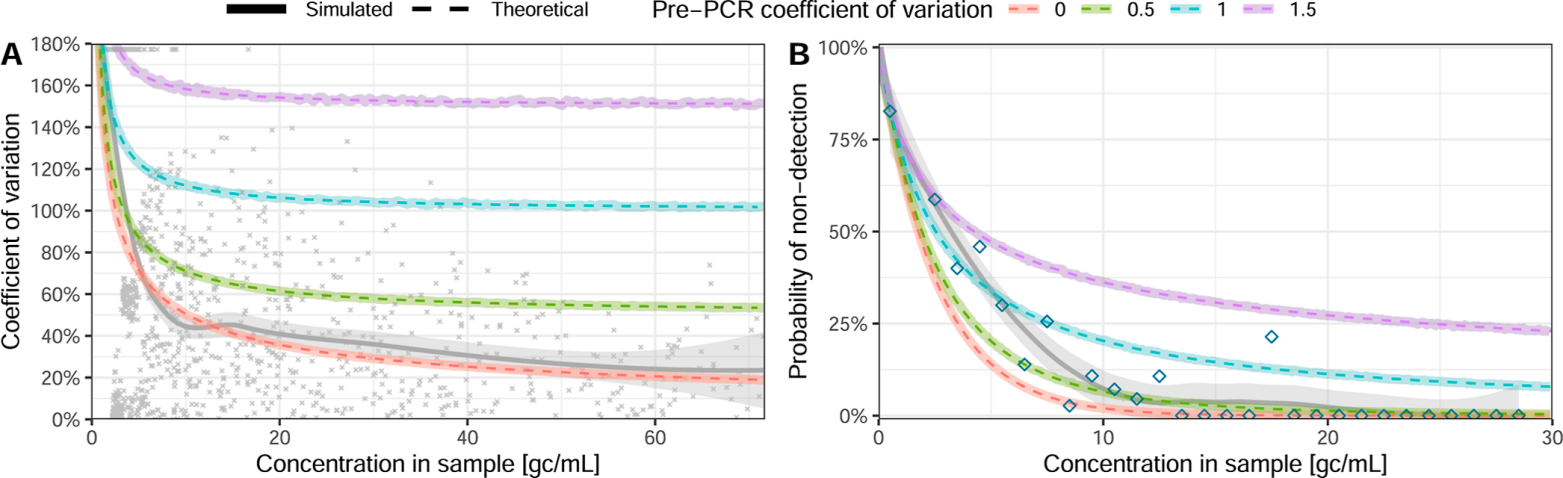
Relationship of the sample concentration with the coefficient of variation and probability of nondetection in digital PCR. Shown is (A) the coefficient of variation (CV) and (B) probability of nondetection (*p*_0_) for a single digital PCR measurement, as a function of the sample concentration *c*. Dashed lines show the CV and *p*_0_ as predicted by eqs 6 and 8, for different strengths of the pre-PCR coefficient of variation ν_pre_ under gamma-distributed pre-PCR noise (assuming a scaling factor of *s* = 30, partition volume of *v* = 0.519 nL, and average partition number of *m* = 22 665). Solid, wide lines show estimates based on simulated dPCR measurements, respectively. Empirical estimates of the CV (dots) and *p*_0_ (diamonds) were computed from duplicate dPCR measurements of Influenza A virus from 14 wastewater treatment plants in Switzerland (Naica Crystal Digital PCR Platform). Gray lines with 95% uncertainty intervals show LOESS-based kernel regressions of the empirical estimates, indicating the relationship with the sample concentration *c*, respectively. As the empirical estimates are based on technical replicates of the same sample, they do not include pre-PCR variation and therefore correspond to ν_pre_ = 0.

For illustration in Figure 1, we also plotted locally estimated scatterplot smoothing (LOESS) curves of empirical estimates for the CV and the probability of nondetection obtained using real-world dPCR data produced by an ongoing wastewater surveillance program by Eawag, Swiss Federal Institute of Aquatic Science and Technology (see Supporting Information, Section E for details on the empirical estimation). These estimates are based on technical replicates of extracted samples that were tested for Influenza A virus and show the same qualitative relationship with the sample concentration as predicted by the theoretical model (see Figure S11 for further pathogens). We note that since the empirical estimates are based on technical replicates of the same preprocessed sample, they correspond to the CV and probability of nondetection without pre-PCR noise.

Using numerical integration, we assessed the accuracy of posterior estimates for the target concentration under our approximate dPCR likelihood (Supporting Information, Section H.1). At low concentrations, the discrete nature of dPCR-based concentration estimates is inaccurately approximated by a continuous density (Figure S14), incurring a limited bias in the posterior distribution for *c* (Figure S20). For example, in a dPCR assay with 25 000 valid partitions, this bias was up to −0.13 gc per mL (assuming a scaling factor of *s* = 30 and partition volume of *v* = 0.519 nL), corresponding to a relative error of up to −1% for concentrations above the 95% detection threshold (Figure S21). This bias is however small compared to the overall uncertainty of posterior estimates for realistic sample sizes. At higher concentrations, the approximation bias approaches zero.

To validate inference across multiple samples with different concentrations using our dPCR-specific likelihood, we simulated measurements from samples whose target concentrations followed a log–linear relationship. We then estimated the slope of this relationship using a generalized linear model. Figure 2 shows estimated slope coefficients for different ground truth slopes, based on 10 simulated dPCR measurements, respectively (see Section 2.6 for details). When partitions count data were available, the coefficients could be correctly estimated using a binomial model for the number of positive partitions (Figure 2A). When partition counts were not available, the dPCR-specific likelihood still recovered the correct coefficients (Figure 2B), while estimates using a normal or log-normal likelihood were miscalibrated (Figure 2C,D). Specifically, estimates under the normal likelihood were highly noisy, and the 95% credible intervals did not consistently cover the true coefficient value. Estimates under the log-normal likelihood showed an upward bias for negative coefficients, i. e. when nondetections were highly probable. In Figures S24–S27 we show that the dPCR-specific likelihood remained well-calibrated across a wide range of concentrations, even when up to 90% of partitions were positive in expectation.

**Figure 2:**
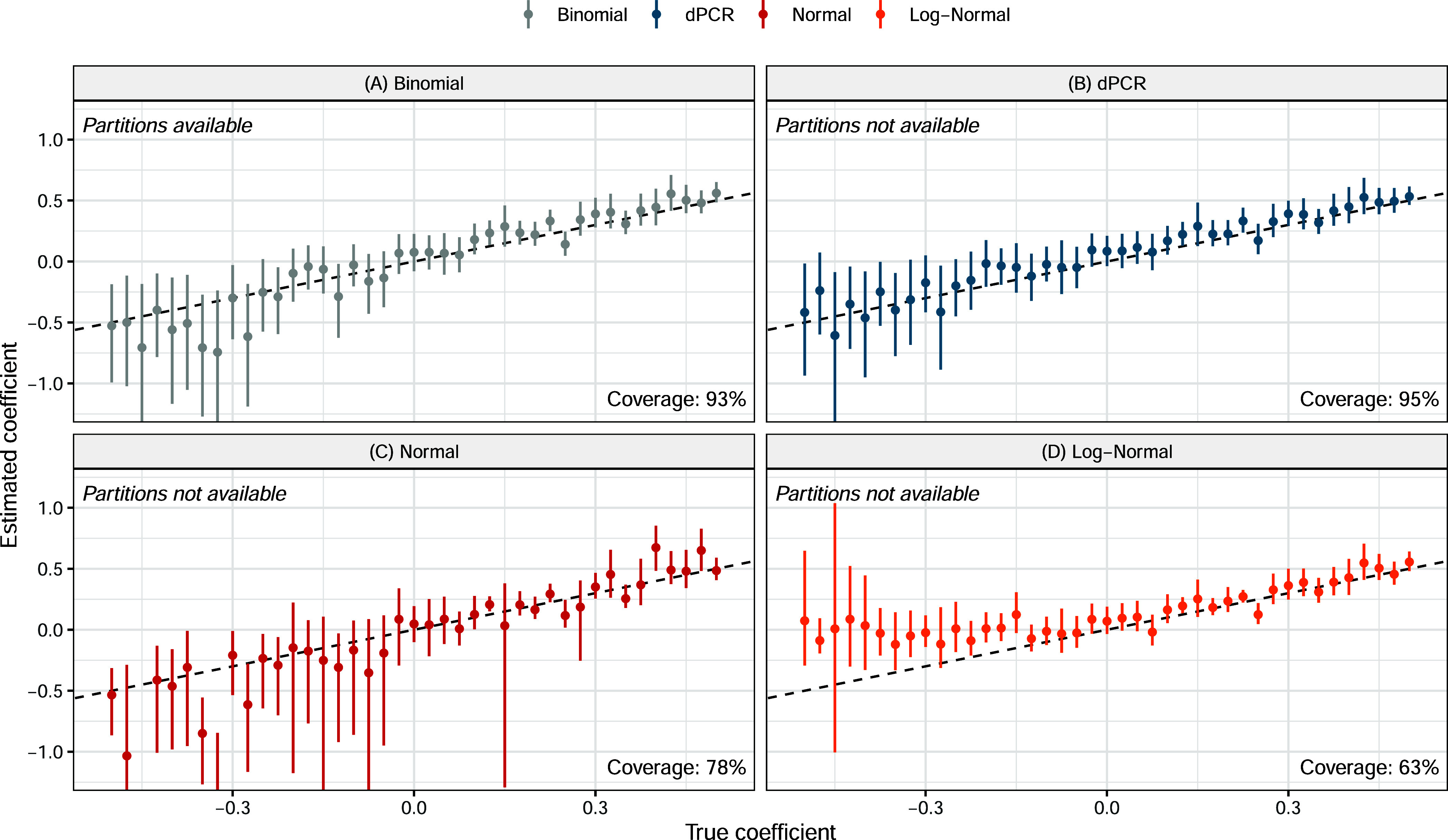
Estimation of regression coefficients from simulated dPCR measurements. Partition counts and estimated concentrations from dPCR were simulated using log–linear regression models with different coefficients. For each simulated data set, the slope coefficient was estimated using 10 simulated dPCR measurements, i. e. (A) with observed partition counts under a binomial model and (B–D) without partition counts (i.e., only reported concentrations) under a dPCR-specific, normal, and log-normal model. Shown are median estimates (dots) with 95% credible intervals (bars). The reported coverage corresponds to the proportion of true simulated coefficients covered the 95% credible intervals of the respective estimates.

### 3.2 Application to eDNA-Based Biomonitoring

We used our dPCR-specific likelihood in a generalized linear model to accurately estimate the half-life of free-eDNA of a marine species in an aquarium seawater experiment by Scriver et al.^26^ In the study, concentrations of the Cytochrome *c* oxidase subunit 1 (COI) gene of the brown bryozoan *B. neritina* were measured in different aquariums with low, medium, and high biomass of the species, using samples taken 0, 4, 8, 24, 48, and 72 h after removal of the organism from the aquarium. Scriver et al. estimated the exponential decay rate of detectable free-eDNA from the averages of all measurements at each point in time (pooled analysis). We stratified this analysis by fitting a separate GLM to the individual samples of each aquarium (Figure 3). Using our dPCR-specific likelihood without partition count data, i. e. only fitting to the reported concentration values, we obtained half-life estimates that closely matched the original pooled estimate of 16.86 h by Scriver et al. and were consistent across aquariums, i. e. 20.15 (95% CrI 10.60–62.51), 13.40 (95% CrI 9.17–23.32), and 15.51 (95% CrI 10.71–30.59) hours. Moreover, these estimates were similar to those obtained using the exact number of positive and total partitions with a binomial likelihood, i. e. 15.60 (95% CrI 8.99–40.16), 14.89 (95% CrI 9.95–26.59), and 15.99 (95% CrI 10.49–33.32) hours for low, medium, and high initial biomass, respectively. In contrast, when fitting a normal or log-normal likelihood to the reported concentration values, half-life estimates became highly uncertain, with inconsistent point estimates across aquariums (Figure 3A). When comparing median concentrations as predicted by the models, predictions under the normal and log-normal models were either below or above those by the binomial and dPCR-specific models (Figure 3B).

**Figure 3:**
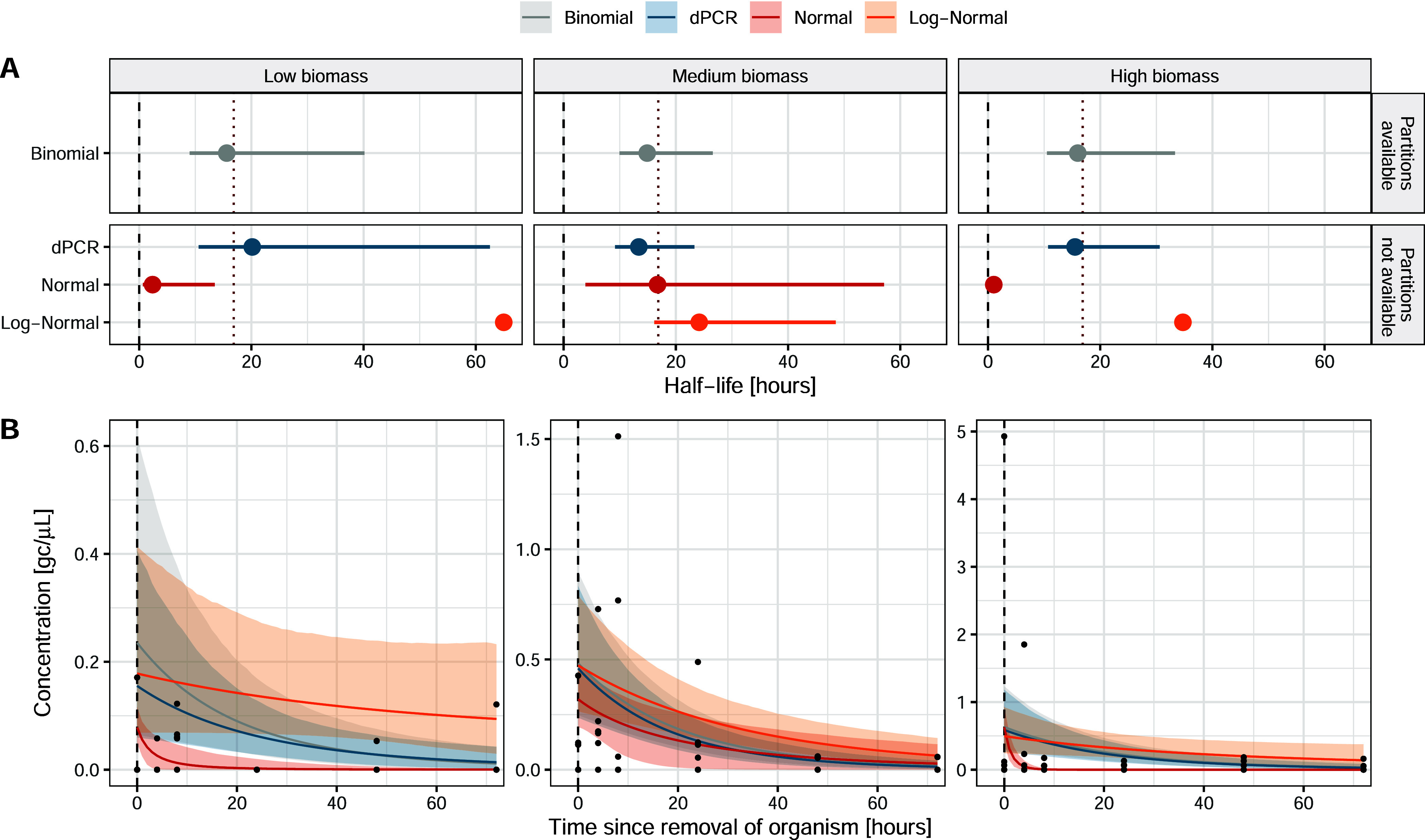
Estimation of eDNA decay for a marine species in aquarium seawater. (A) Estimated half-life period for free-eDNA of *Bugula neritina* in aquarium seawater based on longitudinal dPCR measurements after removal of the organism by Scriver et al.^26^ Shown are median estimates (dots) with 95% credible intervals (bars) from a generalized linear model (GLM) applied to data from an aquarium with low, medium, and high initial biomass, respectively. The GLM was applied to observed partition counts with a binomial likelihood (“gold standard”, gray) and to estimated concentrations with a dPCR-specific (blue), normal (red), and log-normal (orange) likelihood. Red vertical lines show the original pooled estimate reported by Scriver et al. (B) Reported concentrations (dots) and model predictions using the different likelihoods (colored lines) with 95% credible intervals (shaded areas).

### 3.3 Application to Wastewater-Based Epidemiology

The dPCR-specific likelihood enabled accurate estimation of pathogen transmission from wastewater measurements without partition count data. Using dPCR measurements of Influenza A virus at the municipal treatment plant of Zurich, Switzerland, we computed estimates of the viral load in wastewater over time and the effective reproduction number *R*_*t*_ during the winter season 2022/23 (Figure 4, see Figures S29–S33 for the 2023/24 and 2024/25 season). When applying the dPCR-specific likelihood with uncertain assay parameters, i. e. using only broad priors, we obtained viral load and *R*_*t*_ estimates that closely matched those from a binomial model with exact partition counts and assay parameters (mean absolute percentage error MAPE of median load: 1.82%, MAPE of median *R*_*t*_: 0.41%). The posterior predictive distribution for measurements was well-calibrated at low and high concentrations (80% CrIs covered 83.33% of observations both in Oct and Dec, 2022).

**Figure 4:**
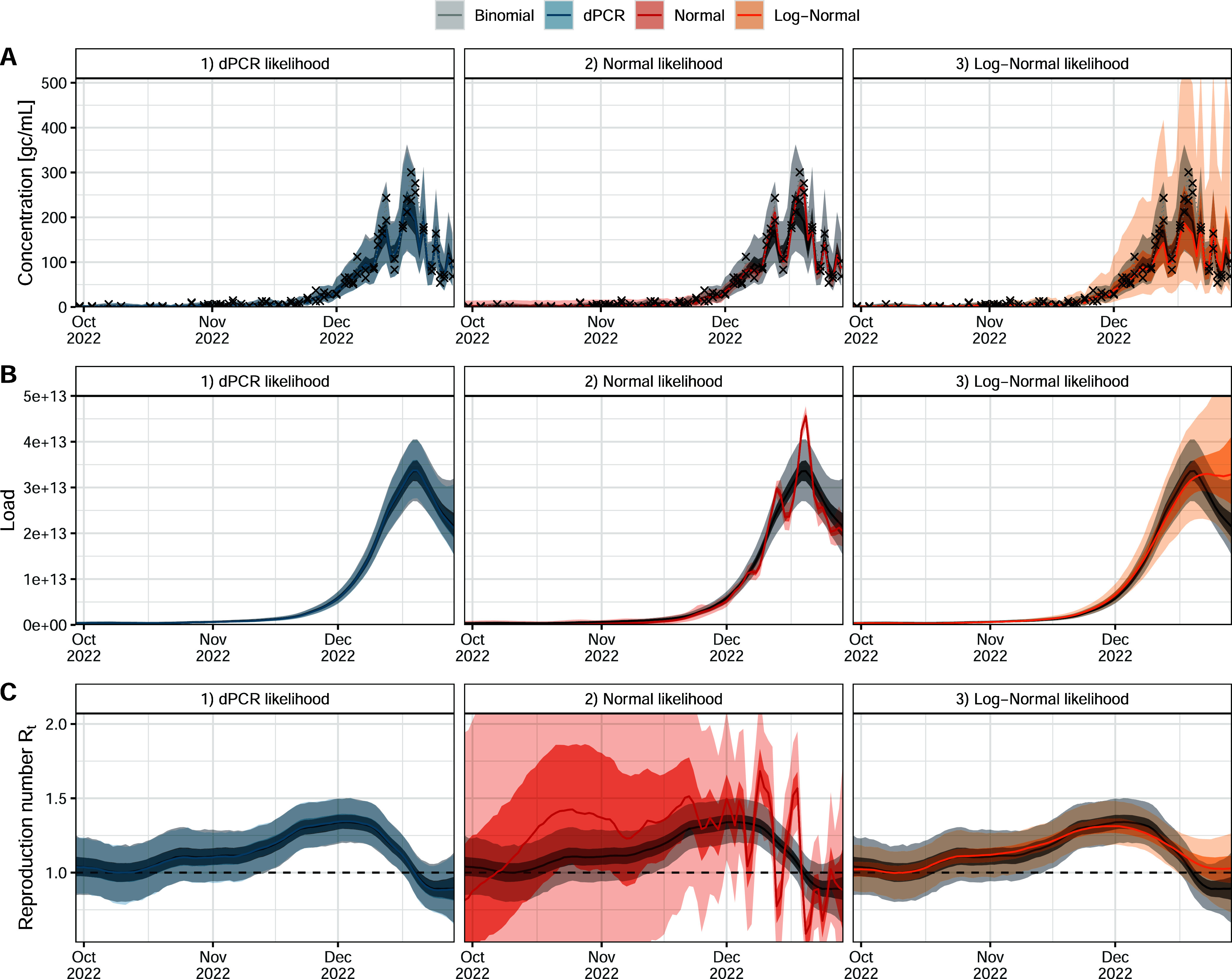
*R*_*t*_ estimation from wastewater measurements. The epidemiological wastewater model (“EpiSewer”) was fitted to duplicate dPCR measurements of Influenza A virus concentrations at the municipal treatment plant of Zurich, Switzerland (Sep 16–Dec 29, 2022) using (1) a dPCR-specific likelihood (blue), (2) a normal likelihood (red), and (3) a log-normal likelihood (orange) for reported concentrations. “Gold standard” estimates using a binomial likelihood for the partition counts and exact assay information are shown in gray. Each panel shows the median (lines) and 50 and 95% credible intervals (strong and weakly shaded areas) of (A) posterior predictive distributions for dPCR measurements, (B) estimated viral loads in wastewater over time, and (C) the estimated effective reproduction number over time. Reported concentrations are shown in panel A (crosses).

In contrast, the use of a normal likelihood resulted in uncertain *R*_*t*_ estimates at the start of the seasonal wave and highly volatile load and *R*_*t*_ estimates at the peak of the wave that differed from those obtained using exact partition count data (MAPE of median load: 50.22%, MAPE of median *R*_*t*_: 16.67%). Credible intervals were too wide at low concentrations (80% CrI coverage in Oct, 2022: 100%) and too narrow at high concentrations (80% CrI coverage in Dec, 2022: 54.76%). Under the log-normal likelihood, load and *R*_*t*_ estimates were less volatile, but also differed from those obtained using exact partition count data (MAPE of median load: 7.95%, MAPE of median *R*_*t*_: 2.74%). Here, credible intervals were too narrow at low concentrations (80% CrI coverage in Oct, 2022: 38.89%) and too wide at high concentrations (80% CrI coverage in Dec, 2022: 100%).

The dPCR-specific model may also provide additional insights about the measurement process even with limited information on the assay parameters. For example, the posterior distribution for ν_pre_ had a median of 19.97% (95% credible interval: 12.05–31.62%) and differed clearly from its prior (Figure S28). As this parameter describes the variation in concentration before the PCR, it allows us to distinguish the technical noise of dPCR from other sources of variation. For instance, using the median estimate for ν_pre_ and the values of *m* and κ, the overall CV at a concentration of 100 gc/mL is predicted as 24.48%, which is 10.32 percentage points higher than the theoretical minimum CV obtained without preprocessing noise or other unexplained variation (14.15%).

## 4 Discussion

In this work, we presented an accurate approach for modeling reported concentration measurements from dPCR when the parameters of the assay and the counts of positive and total partitions are uncertain, as is often the case for publicly reported dPCR data. For this, we developed a dPCR-specific continuous likelihood function that, using priors for the assay parameters, can be fitted directly to concentrations reported by environmental studies and laboratories to estimate an underlying functional relationship across samples. In contrast to simpler models, this likelihood reflects important properties of PCR assays, specifically, that measurement noise is concentration-dependent and that nondetects can occur even if the target gene is present in the sample.

Using both simulated data and real-world examples of dPCR measurements, we showed that estimates from our model closely match those obtained from detailed partition count data using a binomial model. These findings demonstrate that, using a suitable model of the underlying systematic relationship between samples, our model can reliably estimate the association between concentration and other covariates, even when laboratory parameters are not exactly known. In contrast, the use of normal or log-normal models for reported concentrations should be avoided, as they produce noisy and biased estimates, particularly at low concentrations. For example, it is common practice to model log-transformed measurements as normally distributed, yet this will only yield accurate results if all concentrations are sufficiently high, e. g. above the Limit of Quantification, operationally defined as a concentration below which the CV surpasses a fixed threshold such as 30%.^40^ When concentrations are lower, a log-normal model cannot capture the exponential increase in relative measurement error and does not account for zero measurements, which must therefore be removed or imputed, leading to biased estimates. This can impact inferences about underlying biological processes, as we demonstrated for eDNA decay in biomonitoring and pathogen transmission dynamics in wastewater-based epidemiology.

Using Bayesian inference, our approach accounts not only for dPCR noise but also the uncertainty about partition numbers and other parameters of the assay through appropriate priors. In the examples tested, we were able to obtain precise estimates even with broad priors for these parameters by leveraging signal about the error structure using replicate measurements of the same sample or by integrating additional data informing the relationship between samples through a GLM. At the same time, specific laboratory knowledge can be easily incorporated by setting parameters to their known values or by providing informative priors. This is particularly feasible for fixed parameters of the dPCR system, such as the maximum number of partitions or the partition volume, as these are usually specified by the manufacturer. We stress that information about assay parameters is ideally directly provided by the reporting laboratory, but acknowledge that this may not be possible in all settings, particularly when compiling public data from multiple sources. In such a case, our approach could be readily extended by modeling separate assay parameters for each data source or laboratory and providing identical or lab-specific priors for them.

Our findings also have broader implications for data analysis in dPCR-based environmental surveillance. Our examples highlight that environmental models of dPCR data should be applied to the observed partition counts, or, if these are unavailable, to the reported concentrations by using a suitable method as described here. This in contrast to many models currently used in practice, which are often applied to domain-specific indicators derived from the measurements. For example, in wastewater surveillance, pathogen concentrations are typically normalized by flow rates or fecal biomarker concentrations, such as crAssphage or PMMoV.^47,48^ While these indicators of pathogen “load” improve the interpretability of reporting and data visualization, they are not ideal as direct inputs to wastewater models since their measurement distribution depends on the normalization factor. For example, depending on the flow rate, the same “load” may correspond to vastly different concentrations, with different measurement noise and probabilities of nondetection. The same applies to concentrations normalized by fecal markers, with the added complication that marker concentrations may themselves be subject to measurement noise from digital or quantitative PCR. Therefore, we recommend modeling the reported, unadjusted concentrations as observations and incorporating important environmental factors as covariates into the model, as for example described for wastewater flow rates in Section 2.8.

Moreover, for modeling dPCR measurements at low concentrations, a hurdle model approach as used in this work should be preferred over censoring approaches in which zero measurements or measurements below the LoD are replaced with a predefined value.^34,49^ As has been previously noted, modeling nondetects from digital PCR as censored data can lead to bias.^50^ This is in part because censoring uses a fixed threshold, while the probability of nondetection gradually increases as the concentration approaches zero. Furthermore, censoring applies to the measurements, which are subject to noise, not to the true concentration in the sample. Similar arguments apply to the LoQ, considering the gradual increase of the CV as concentrations approach zero and the fact that the CV depends on the true sample concentration, not the measurement. We thus argue that it is better to model the increase in CV at low concentrations than to classify measurements by a predefined threshold. Indeed, removing measurements below the LoQ from statistical analyses may lead to an overestimation of concentrations.

We note a few limitations of our model. First, in line with the dMIQE guidelines,^22^ our estimators for the CV and probability of nondetection account not only for Poisson error but also for other variation before the PCR. While in principle, any positive continuous distribution with a well-defined moment generating function (MGF) could be used, we have here only tested log-normally and gamma-distributed pre-PCR noise, for which either a closed-form expression or approximation of the MGF are available. We acknowledge that choosing a pre-PCR noise distribution can be difficult, as this parameter summarizes all unexplained variation in a model. For the CV, we found that the exact distribution of pre-PCR noise is irrelevant for most concentration values and can be well approximated using a Taylor series expansion. For the probability of nondetection, however, we found notable differences between log-normally and gamma-distributed pre-PCR noise and no suitable Taylor series approximation. Second, our estimators for the CV are asymptotic and assume nonsaturation of the assay through appropriate dilution, where the probability of all partitions being positive is almost zero. Under extreme conditions where the saturation of the assay is approached, the CV becomes undefined, and our approximation does not apply. Third, we assumed that dPCR measurements are in principle unbiased, i. e. we do not account for unknown systematic distortions of dPCR measurements such as from miscalibration of the partition volume.^51^ In our applications, we also assumed that assay parameters are stable over time. Systematic changes in the laboratory protocol would require explicit modeling. We also highlight that in our examples, concentrations were measured in gene copies per volume, but in the case of solids-based extraction, measurements can also have other units, such as gene copies per gram of dry weight.^47^ Fourth, we did not account for partition volume variation, which can be an additional source of bias in droplet-based systems.^35^ Fifth, our hurdle likelihood approximates the joint effect of pre-PCR noise on nondetection and on the distribution of nonzero measurements by separately marginalizing over pre-PCR variation when computing nondetection probability and the CV of concentration estimates. As a result, it does not fully capture the correlation between these quantities induced by the underlying pre-PCR noise. However, we find that the accuracy of our likelihood is not significantly affected by this approximation (Supporting Information, Section F.4). Sixth, our model assumes that technical replicates are pooled into a single concentration estimate, as is common practice. If measurements have not yet been pooled across technical replicates and no partition data are available (such that the pooled maximum likelihood estimate cannot be computed), an alternative is to use the arithmetic mean of individual concentration estimates (leading to a small bias, as discussed in Supporting Information, Section A.2). Note that our approximations for the CV and the probability of nondetection for measurements assume that all technical replicates are based on the same preprocessed sample, and therefore only include the effects of Poisson error and partition number variation between technical replicates. Seventh, reported concentration values from digital PCR are discrete in theory, and our continuous likelihood approximates their underlying probabilities only with limited accuracy. We have shown that this leads to a bias at low concentrations that is, however, small compared to the overall uncertainty of estimates under limited sample sizes. Finally, since this work focused on the quantification of targets already present in the environment, we assumed that the true concentration can be arbitrarily small but is larger than zero. If, instead, the objective is to determine whether a particular target is present or absent, it may also be important to model the probability of false positives.^40^

## 5 Conclusions

We have developed a dPCR-specific likelihood, implemented in the R package “dPCRfit”,^42^ providing a Bayesian generalized linear model for regression analyses of dPCR data, e.g., to improve eDNA abundance estimates in species inventorying.^30,33^ We have also integrated the likelihood as a module in the “EpiSewer” package, e.g., to improve wastewater-based estimates of pathogen transmission rates.^43^ When the detailed partition counts and assay parameters are known, these packages also support direct fitting to the number of positive partitions in the assay, which remains the most accurate approach for analyzing dPCR data. However, in settings where this information is not available or impractical to obtain, our dPCR-specific likelihood enables reliable inferences from reported concentration data only. While this work has focused on digital PCR, analogous statistical relationships apply to quantitative PCR methods and can be modeled in a similar vein.^36,52^ Overall, we envision that accurate modeling of laboratory and measurement processes will improve environmental surveillance and provide new insights into environmental variability by disentangling it from measurement noise.

## Supplementary Material



## Data Availability

All data, code for simulation, and analysis scripts are publicly available at https://github.com/adrian-lison/dPCR-observation-model-study.

## Notes

Additional details on the methods, mathematical derivations, simulation results, and sensitivity analyses (PDF)

## References

[1] Kelly R. P., Lodge D. M., Lee K. N., Theroux S., Sepulveda A. J., Scholin C. A., Craine J. M., Allan E. A., Nichols K. M., Parsons K. M. (2024). Toward a National eDNA Strategy for the United
States. Environ. DNA.

[2] Beng K. C., Corlett R. T. (2020). Applications of Environmental DNA (eDNA) in Ecology
and Conservation: Opportunities, Challenges and Prospects. Biodiversity Conserv..

[3] Barnes M. A., Turner C. R. (2016). The Ecology of Environmental DNA
and Implications for
Conservation Genetics. Conserv. Genet..

[4] Rees H. C., Maddison B. C., Middleditch D. J., Patmore J. R. M., Gough K. C. (2014). REVIEW:
The Detection of Aquatic Animal Species Using Environmental DNA –
a Review of eDNA as a Survey Tool in Ecology. J. Appl. Ecol..

[5] Keshaviah A., Diamond M. B., Wade M. J., Scarpino S. V., Ahmed W., Amman F. (2023). Wastewater Monitoring Can Anchor Global Disease Surveillance
Systems. Lancet Global Health.

[6] Fernandez-Cassi X., Scheidegger A., Bänziger C., Cariti F., Tuñas
Corzon A., Ganesanandamoorthy P. (2021). Wastewater Monitoring
Outperforms Case Numbers as a Tool to Track COVID-19 Incidence Dynamics
When Test Positivity Rates Are High. Water Res..

[7] Ho J., Stange C., Suhrborg R., Wurzbacher C., Drewes J. E., Tiehm A. (2022). SARS-CoV-2 Wastewater Surveillance
in Germany: Long-term RT-digital Droplet PCR Monitoring, Suitability
of Primer/Probe Combinations and Biomarker Stability. Water Res..

[8] Bivins A., Bibby K. (2021). Wastewater Surveillance
during Mass COVID-19 Vaccination on a College
Campus. Environ. Sci. Technol. Lett..

[9] Toribio-Avedillo D., Gómez-Gómez C., Sala-Comorera L., Rodríguez-Rubio L., Carcereny A., García-Pedemonte D. (2023). Monitoring Influenza
and Respiratory Syncytial Virus in Wastewater. Beyond COVID-19. Sci. Total Environ..

[10] Julian T. R., Devaux A. J., Brülisauer L., Conforti S., Rusch J. C., Gan C., Bagutti C., Stadler T., Kohn T., Ort C. (2024). Monitoring
an Emergent Pathogen at Low Incidence in Wastewater Using qPCR: Mpox
in Switzerland. Food Environ. Virol..

[11] Boehm A. B., Hughes B., Duong D., Chan-Herur V., Buchman A., Wolfe M. K. (2023). Wastewater
Concentrations
of Human Influenza, Metapneumovirus, Parainfluenza, Respiratory Syncytial
Virus, Rhinovirus, and Seasonal Coronavirus Nucleic-Acids during the
COVID-19 Pandemic: A Surveillance Study. Lancet
Microbe.

[12] Conforti S., Holschneider A., Sylvestre É., Julian T. R. (2024). Monitoring ESBL-Escherichia
Coli in Swiss Wastewater between November 2021 and November 2022:
Insights into Population Carriage. mSphere.

[13] Huisman J. S., Scire J., Caduff L., Fernandez-Cassi X., Ganesanandamoorthy P., Kull A., Scheidegger A., Stachler E., Boehm A. B., Hughes B. (2022). Wastewater-Based
Estimation of the Effective Reproductive Number of SARS-CoV-2. Environ. Health Perspect..

[14] Basu A. S. (2017). Digital
Assays Part I: Partitioning Statistics and Digital PCR. SLAS TECHNOLOGY: Translating Life Sciences Innovation.

[15] Dorazio R. M., Hunter M. E. (2015). Statistical Models
for the Analysis and Design of Digital
Polymerase Chain Reaction (dPCR) Experiments. Anal. Chem..

[16] Doi H., Takahara T., Minamoto T., Matsuhashi S., Uchii K., Yamanaka H. (2015). Droplet Digital
Polymerase Chain
Reaction (PCR) Outperforms Real-Time PCR in the Detection of Environmental
DNA from an Invasive Fish Species. Environ.
Sci. Technol..

[17] Ahmed W., Smith W. J. M., Metcalfe S., Jackson G., Choi P. M., Morrison M. (2022). Comparison of RT-qPCR and RT-dPCR Platforms
for the Trace Detection of SARS-CoV-2 RNA in Wastewater. ACS ES&T Water.

[18] Tiwari A., Ahmed W., Oikarinen S., Sherchan S. P., Heikinheimo A., Jiang G. (2022). Application
of Digital PCR for Public Health-Related
Water Quality Monitoring. Sci. Total Environ..

[19] Ciesielski M., Blackwood D., Clerkin T., Gonzalez R., Thompson H., Larson A. (2021). Assessing Sensitivity and Reproducibility of RT-ddPCR
and RT-qPCR for the Quantification of SARS-CoV-2 in Wastewater. J. Virol. Methods.

[20] Vynck M., Vandesompele J., Nijs N., Menten B., De Ganck A., Thas O. (2016). Flexible Analysis
of Digital PCR Experiments Using Generalized Linear
Mixed Models. Biomol. Detect. Quantif..

[21] Petterson S. R., Dumoutier N., Loret J. F., Ashbolt N. J. (2009). Quantitative Bayesian
Predictions of Source Water Concentration for QMRA from Presence/Absence
Data for E. Coli O157:H7. Water Sci. Technol..

[22] Huggett J. F., The dMIQE Group (2020). The Digital
MIQE Guidelines Update: Minimum Information for Publication of Quantitative
Digital PCR Experiments for 2020. Clin. Chem..

[23] Naughton C. C., Roman F. A., Alvarado A. G. F., Tariqi A. Q., Deeming M. A., Kadonsky K. F., Bibby K., Bivins A., Medema G., Ahmed W. (2023). Show Us
the Data: Global COVID-19 Wastewater Monitoring
Efforts, Equity, and Gaps. FEMS Microbes.

[24] Kaplan E. H., Wang D., Wang M., Malik A. A., Zulli A., Peccia J. (2020). Aligning SARS-CoV-2 Indicators via an Epidemic Model:
Application to Hospital Admissions and RNA Detection in Sewage Sludge. Health Care Management Science.

[25] Vallejo J. A., Trigo-Tasende N., Rumbo-Feal S., Conde-Pérez K., López-Oriona A.
´., Barbeito I. (2022). Modeling
the Number of People Infected with SARS-COV-2 from Wastewater Viral
Load in Northwest Spain. Sci. Total Environ..

[26] Scriver M., von Ammon U., Youngbull C., Pochon X., Stanton J. A. L., Gemmell N. J. (2024). Drop It All: Extraction-Free Detection
of Targeted Marine Species through Optimized Direct Droplet Digital
PCR. PeerJ.

[27] Capo E., Spong G., Königsson H., Byström P. (2020). Effects of
Filtration Methods and Water Volume on the Quantification of Brown
Trout (Salmo Trutta) and Arctic Char (Salvelinus Alpinus) eDNA Concentrations
via Droplet Digital PCR. Environ. DNA.

[28] Wood S. A., Pochon X., Laroche O., von Ammon U., Adamson J., Zaiko A. (2019). A Comparison of Droplet
Digital Polymerase
Chain Reaction (PCR), Quantitative PCR and Metabarcoding for Species-Specific
Detection in Environmental DNA. Mol. Ecol. Resour..

[29] Thalinger B., Wolf E., Traugott M., Wanzenböck J. (2019). Monitoring
Spawning Migrations of Potamodromous Fish Species via eDNA. Sci. Rep..

[30] Chucholl F., Fiolka F., Segelbacher G., Epp L. S. (2021). eDNA Detection of
Native and Invasive Crayfish Species Allows for Year-Round Monitoring
and Large-Scale Screening of Lotic Systems. Frontiers in Environmental Science.

[31] Li G., Denise H., Diggle P., Grimsley J., Holmes C., James D. (2023). A Spatio-Temporal
Framework for Modelling Wastewater
Concentration during the COVID-19 Pandemic. Environ. Int..

[32] Renninger N., Nastasi N., Bope A., Cochran S. J., Haines S. R., Balasubrahmaniam N., Stuart K., Bivins A., Bibby K., Hull N. M. (2021). Indoor Dust as a Matrix for Surveillance of COVID-19. mSystems.

[33] Capo E., Spong G., Norman S., Königsson H., Bartels P., Byström P. (2019). Droplet Digital PCR Assays for the
Quantification of Brown Trout (Salmo Trutta) and Arctic Char (Salvelinus
Alpinus) from Environmental DNA Collected in the Water of Mountain
Lakes. PLoS One.

[34] Uthicke S., Lamare M., Doyle J. R. (2018). eDNA Detection of
Corallivorous Seastar
(Acanthaster Cf. Solaris) Outbreaks on the Great Barrier Reef Using
Digital Droplet PCR. Coral Reefs.

[35] Jacobs B. K., Goetghebeur E., Clement L. (2014). Impact of Variance Components on
Reliability of Absolute Quantification Using Digital PCR. BMC Bioinf..

[36] Forootan A., Sjöback R., Björkman J., Sjögreen B., Linz L., Kubista M. (2017). Methods to
Determine Limit of Detection
and Limit of Quantification in Quantitative Real-Time PCR (qPCR). Biomol. Detect. Quantif..

[37] Dube S., Qin J., Ramakrishnan R. (2008). Mathematical Analysis of Copy Number Variation in a
DNA Sample Using Digital PCR on a Nanofluidic Device. PLoS One.

[38] Borchardt M. A., Boehm A. B., Salit M., Spencer S. K., Wigginton K. R., Noble R. T. (2021). The Environmental Microbiology Minimum Information
(EMMI) Guidelines: qPCR and dPCR Quality and Reporting for Environmental
Microbiology. Environ. Sci. Technol..

[39] White H. (1980). A Heteroskedasticity-Consistent
Covariance Matrix Estimator and a Direct Test for Heteroskedasticity. Econometrica.

[40] Armbruster D. A., Pry T. (2008). Limit of Blank, Limit of Detection and Limit of Quantitation. Clinical Biochemist Reviews.

[41] Stan Development Team . Stan Modeling Language Users Guide and Reference Manual; Version 2.31, 2022.

[42] Lison, A. dPCRfit: Fit regression models to digital PCR data. Zenodo, 2025. https://zenodo.org/records/16942055.

[43] Lison, A. EpiSewer: Estimate Epidemiological Parameters from Wastewater Measurements. Zenodo, 2024. https://zenodo.org/records/13899759.

[44] Cori A., Ferguson N. M., Fraser C., Cauchemez S. (2013). A New Framework
and Software to Estimate Time-Varying Reproduction Numbers during
Epidemics. Am. J. Epidemiol..

[45] Ferguson N. M., Cummings D. A. T., Cauchemez S., Fraser C., Riley S., Meeyai A. (2005). Strategies for Containing an Emerging Influenza Pandemic
in Southeast Asia. Nature.

[46] Carrat F., Vergu E., Ferguson N. M., Lemaitre M., Cauchemez S., Leach S. (2008). Time Lines of Infection
and Disease in Human Influenza:
A Review of Volunteer Challenge Studies. Am.
J. Epidemiol..

[47] Graham K. E., Loeb S. K., Wolfe M. K., Catoe D., Sinnott-Armstrong N., Kim S. (2021). SARS-CoV-2 RNA in Wastewater Settled Solids Is Associated
with COVID-19 Cases in a Large Urban Sewershed. Environ. Sci. Technol..

[48] Greenwald H. D., Kennedy L. C., Hinkle A., Whitney O. N., Fan V. B., Crits-Christoph A. (2021). Tools for Interpretation of Wastewater
SARS-CoV-2 Temporal and Spatial Trends Demonstrated with Data Collected
in the San Francisco Bay Area. Water Res. X.

[49] Safford H., Zuniga-Montanez R. E., Kim M., Wu X., Wei L., Sharpnack J. (2022). Wastewater-Based Epidemiology for COVID-19:
Handling qPCR Nondetects and Comparing Spatially Granular Wastewater
and Clinical Data Trends. ACS ES&T Water.

[50] Chik A. H. S., Schmidt P. J., Emelko M. B. (2018). Learning
Something From Nothing:
The Critical Importance of Rethinking Microbial Non-detects. Front. Microbiol..

[51] Emslie K. R., H McLaughlin J. L., Griffiths K., Forbes-Smith M., Pinheiro L. B., Burke D. G. (2019). Droplet
Volume Variability and Impact
on Digital PCR Copy Number Concentration Measurements. Anal. Chem..

[52] Jones M., Matechou E., Cole D., Diana A., Griffin J., Peixoto S., Handley L. L., Buxton A. (2025). More than
Presence-Absence;
Modelling (e)­DNA Concentration across Time and Space from qPCR Survey
Data. Journal of Statistical Theory and Practice.

