# Supplementary material for: Improving Inference from Reported Concentrations in Environmental Surveillance by Modeling the Statistical Features of Digital PCR
Source: ACS ES T Water. 2026 Jun 10;6(7):4119–29. doi: 10.1021/acsestwater.5c01051 (PMC13366574; doi:10.1021/acsestwater.5c01051)
Supplement: Supplementary file 1 [file ew5c01051_si_001.pdf]

Supporting Information: Improving inference from reported concentrations in environmental surveillance by modeling the statistical features of digital PCR

Adrian Lison[a,b*], Timothy R. Julian[c,d,e], and Tanja Stadler[a,b]

[a]ETH Zurich, Department of Biosystems Science and Engineering, Basel, 4056, Switzerland

[b]SIB Swiss Institute of Bioinformatics, Lausanne, 1015, Switzerland

[c]Eawag, Swiss Federal Institute of Aquatic Science and Technology, Dubendorf, 8600, Switzerland

[d]Swiss Tropical and Public Health Institute, Allschwil, 4123, Switzerland

[e]University of Basel, Basel, 4055, Switzerland

[*]Corresponding author: adrian.lison@bsse.ethz.ch

Contents

A Bias of concentration estimates **2**

A.1 Maximum-likelihood estimator . . . 2

A.2 Averaging of dPCR measurements . . . 4

B Coefficient of variation of concentration estimates **6**

B.1 CV without pre-PCR variation . . . 6

B.2 CV with pre-PCR variation . . . 7

Gamma distribution ▪ Log-normal distribution ▪ Taylor series expansion

C Probability of non-detection **10**

C.1 Relationship between probability of non-detection and limit of detection . . . 10

C.2 Probability of non-detection with pre-PCR variation . . . 10

D Simulation analysis **11**

D.1 Coefficient of variation . . . 11

D.2 Probability of non-detection . . . 16

E Comparison with empirical data **17**

F Likelihoods for non-zero dPCR measurements **20**

F.1 Conditional distribution of non-zero measurements . . . 20

F.2 Back-computation of binomial likelihood . . . 21

F.3 Comparison of continuous measurement distributions 22

F.4 Continuous approximation to the binomial likelihood 23

F.5 Identifiability of c and κ 28

G Inference from dPCR measurements **29**

G.1 Priors for assay parameters 29

Number of total partitions ▪ Conversion factor ▪ Pre-PCR coefficient of variation

G.2 Estimation 30

H Validation of inference **32**

H.1 Posterior distribution under approximate likelihood 32

H.2 Estimating a single concentration 36

H.3 Estimating coefficients of a regression model 36

I Application to eDNA-based biomonitoring **43**

I.1 Model 43

I.2 Assay parameters 43

J Application to wastewater-based epidemiology **44**

J.1 Assay parameters 45

J.2 Additional results 46

References **52**

A Bias of concentration estimates

A.1 Maximum-likelihood estimator

Dorazio and Hunter [1] describe a maximum likelihood estimator for the target concentration c

$$\hat{c} = -\frac{1}{\kappa} \log\left(1 - \frac{y}{m}\right), \tag{1}$$

when y out of a total of m valid partitions were positive. As shown by Dube et al. [2], $\hat{c}$ is an asymptotically unbiased estimator of the true concentration, i. e.

$$\lim_{m\to\infty} \mathbb{E}[\hat{C}_m] = c. \tag{2}$$

However, this relationship is only valid asymptotically. We computed the relative bias of the estimator at finite m using the distribution of $\hat{c}$ values implied by the binomial distribution of positive partition counts y for a given concentration c (and conditional on $y < m$). We find that $\hat{c}$ has a small bias that is on the order of 0.001–0.01% for typical digital PCR systems with 10000–50000 total partitions (Figure S1). Even for systems with lower partition numbers per sample (≈ 1000), the bias remains rather low, i. e. below 0.1% for $\lambda \leq 1$. In Supplement B, we use Eq. (2) to derive approximations for the coefficient of variation (CV) of $\hat{c}$. The bias of $\hat{c}$ at finite m thus also influences our CV approximations. For example, when $\hat{c}$ has a relative bias of τ, using Eq. (2) to obtain the coefficient of variation given the asymptotic variance of $\hat{c}$ incurs a bias of $-\frac{\tau}{1+\tau}$ in the CV estimate. For digital PCR systems with 10000–50000 total partitions, this bias is also rather small, i. e. on the order of -0.005 – -0.01%.

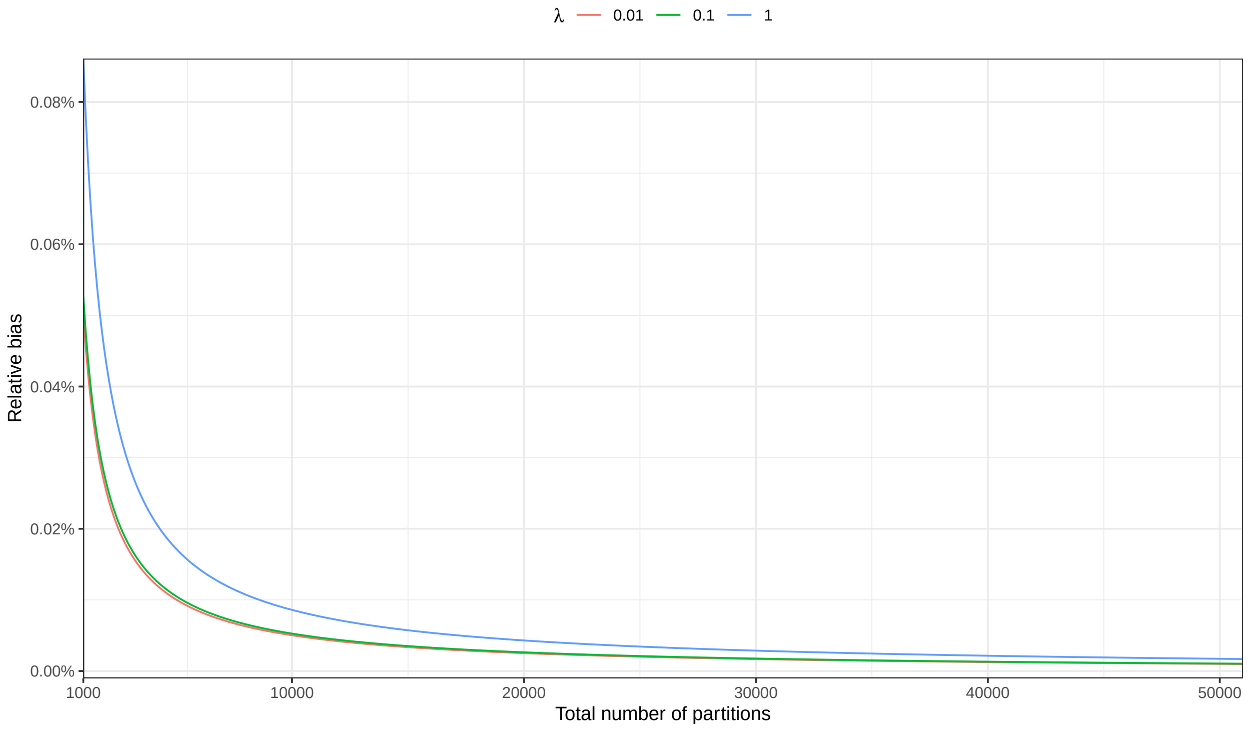

Fig S1. Relative bias of the maximum-likelihood concentration estimator $\hat{c}$. Shown is the bias of $\hat{c}$ as a function of the total number of valid partitions in a digital PCR assay. The bias was numerically computed for different target concentration of λ, i. e. 0.01, 0.1, and 1, using a weighted sum of possible $\hat{c}$ values with the binomial probabilities as weights. The relative bias is larger for larger values of λ.

A.2 Averaging of dPCR measurements

There exist different approaches to combine replicate measurements $i \in \{1, \dots, n\}$ of a sample into a single concentration estimate. Dorazio and Hunter [1] propose the pooled ML estimate

$$\hat{c}^{\mathrm{ML}} = -\frac{1}{\kappa} \log\left(1 - \frac{\overline{y}}{\overline{m}}\right) = -\frac{1}{\kappa} \log\left(1 - \frac{\sum_{i=1}^{n} y_i}{\sum_{i=1}^{n} m_i}\right), \tag{3}$$

where the replicate runs had $m_1, \dots, m_n$ total partitions and $y_1, \dots, y_n$ positive partitions, respectively. This approach is typically used when pooling partition counts from several wells.

A different approach also used in practice is to compute the arithmetic mean of individual ML estimates for each replicate

$$\hat{c}^{\mathrm{AVG}} = \frac{1}{n} \sum_{i=1}^{n} \hat{c}_i = \frac{1}{n} \sum_{i=1}^{n} \left(-\frac{1}{\kappa} \log\left(1 - \frac{y_i}{m_i}\right)\right). \tag{4}$$

In essence, the ML estimate in Eq. (3) differs from the arithmetic mean of estimates in Eq. (4) in two regards. First, the ML estimate computes an average of the estimated probabilities $p_1, \dots, p_n$ for positive partitions, not an average of the concentrations. Second, this average is weighted by the total number of partitions of each replicate, while the arithmetic mean gives equal weight to all replicates.

To assess the magnitude of these differences, we simulated duplicate dPCR measurements for various concentrations. While the first replicate was assumed to have 25000 total partitions, we simulated the second replicate to have between 5000 and 25000 partitions in order to study the effect of differences in the total number of partitions. We assumed a scaling factor of $s = 30$ and a partition volume of $v = 0.519\,\mathrm{nL}$. As a first step, we compared the arithmetic mean estimate $\hat{c}^{\mathrm{AVG}}$ with an unweighted ML estimate

$$\hat{c}^{\mathrm{uML}} = -\frac{1}{\kappa} \log\left(1 - \frac{1}{n} \sum_{i=1}^{n} \frac{y_i}{m_i}\right), \tag{5}$$

in which each replicate receives the same weight. Thereby, we could isolate the effect of averaging concentrations instead of averaging probabilities. Figure S2 shows how the arithmetic mean of measurements deviates from the unweighted ML estimate. We find that the arithmetic mean has a small positive bias compared to the unweighted ML estimate, as the concentration estimate is a

non-linear function of the probability of positive partitions, such that the average of concentrations is not equal to the concentration estimate based on the average probability. The potential deviation increases linearly with the concentration in the PCR, however even for high concentrations of 1000 gc/mL, the deviation is considerably less than 1 gc/mL.

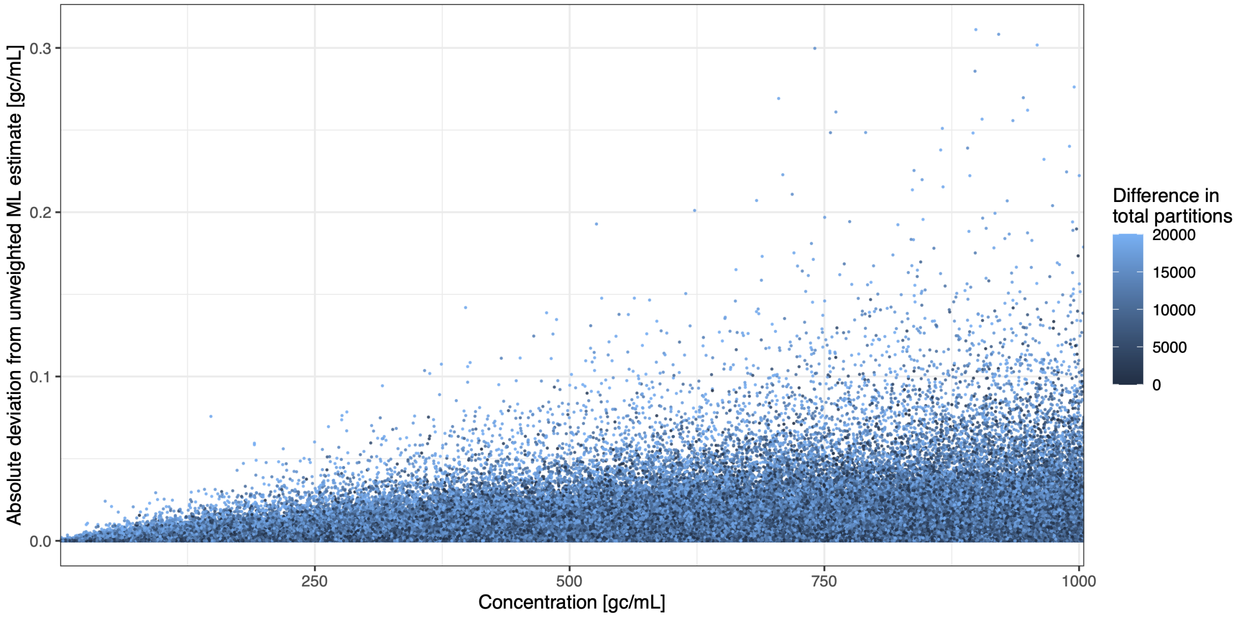

Fig S2. Comparison of arithmetic mean estimate with unweighted ML estimate of concentrations. Shown are deviations of the arithmetic mean of replicate measurements from an unweighted ML estimate for simulated measurements as a function of concentration, and for different degrees of partition number variation between replicates. Simulated measurements assume a scaling factor of $s = 30$ and partition volume of $v = 0.519\,\text{nL}$.

Next, we compared the unweighted ML estimate defined in Eq. (5) to the weighted ML estimate defined in Eq. (3). We found larger deviations than between the arithmetic mean and unweighted ML estimate, but no systematic bias (Figure S3). Deviations were particularly large in cases where one of the replicates only had few total partitions, such that the corresponding concentration estimate was noisy. In the arithmetic mean and the unweighted ML estimate, such replicates are weighted equally to other replicates. The ML estimate defined in Eq. (3) gives more weight to replicates with more partitions, making it less sensitive to noisy measurements based on few partitions. In practice, however, differences in the total number of partitions larger than 5000 do not occur frequently in dPCR assays. For smaller differences in the total number of partitions, the differences between unweighted and weighted estimates are considerably smaller. Overall, we conclude that the ML estimate of multiple replicates has higher accuracy and precision than the arithmetic mean of

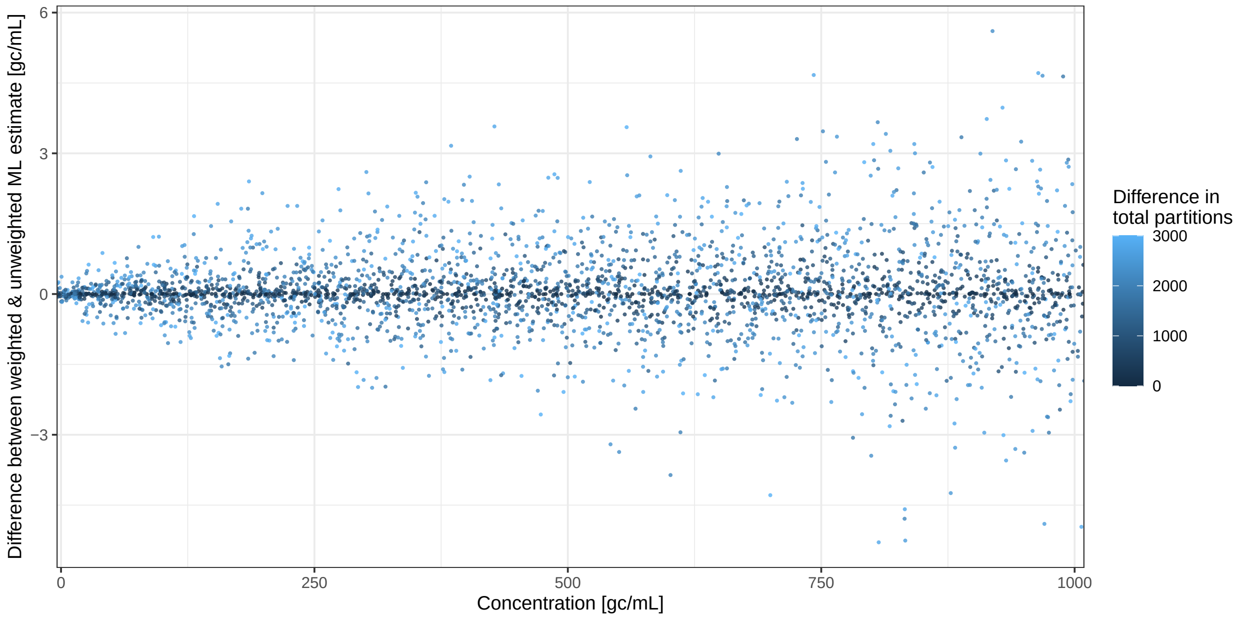

Fig S3. Comparison of unweighted and weighted ML estimates of concentrations. Shown are deviations of the unweighted ML estimate from the weighted ML estimate for simulated measurements as a function of concentration, and for different degrees of partition number variation between replicates. Simulated measurements assume a scaling factor of $s = 30$ and partition volume of $v = 0.519\,\text{nL}$.

individual estimates, but in practice the differences will be small compared to the overall uncertainty of measurements, except in cases where the total number of partitions varies greatly.

B Coefficient of variation of concentration estimates

B.1 CV without pre-PCR variation

The variance of the maximum likelihood concentration estimate $\hat{C} = -\frac{1}{\kappa}\log\Big(1 - \frac{\sum_{i=1}^{n} Y_i}{\sum_{i=1}^{n} m_i}\Big)$ is

$$\mathrm{Var}[\hat{C}] = \mathrm{Var}\left[-\frac{1}{\kappa}\log\Big(1 - \frac{\sum_{i=1}^{n} Y_i}{\sum_{i=1}^{n} m_i}\Big)\right] = \frac{1}{\kappa^2}\mathrm{Var}\left[\log\Big(1 - \frac{\sum_{i=1}^{n} Y_i}{\sum_{i=1}^{n} m_i}\Big)\right]. \tag{6}$$

Using $\mathrm{Var}[\log(X)] \approx \frac{\mathrm{Var}[X]}{\mathbb{E}[X]^2}$, we obtain the asymptotic approximation

$$\mathrm{Var}[\hat{C}] \approx \frac{1}{\kappa^2(\sum_{i=1}^{n} m_i)^2} \frac{\mathrm{Var}[\sum_{i=1}^{n} Y_i]}{\left(1 - \mathbb{E}\left[\frac{\sum_{i=1}^{n} Y_i}{\sum_{i=1}^{n} m_i}\right]\right)^2}. \tag{7}$$

As the sum $\sum_{i=1}^{n} Y_i$ is also binomially distributed with variance $\mathrm{Var}[\sum_{i=1}^{n} Y_i] = (\sum_{i=1}^{n} m_i)\, p\,(1-p)$, and probability of success $p = \mathbb{E}\left[\frac{\sum_{i=1}^{n} Y_i}{\sum_{i=1}^{n} m_i}\right]$, we further get

$$\mathrm{Var}[\hat{C}] \approx \frac{1}{\kappa^2 (\sum_{i=1}^{n} m_i)^2} \frac{(\sum_{i=1}^{n} m_i)\, p\,(1-p)}{(1-p)^2} = \frac{1}{\kappa^2 \sum_{i=1}^{n} m_i} \frac{p}{(1-p)}. \tag{8}$$

Finally, using the probability for a positive partition $p = 1 - \exp(-\lambda)$ [1], we obtain

$$\mathrm{Var}[\hat{C}] \approx \frac{1}{\kappa^2 \sum_{i=1}^{n} m_i} \frac{1-\exp(-\lambda)}{(\exp(-\lambda))} = \frac{1}{\kappa^2 \sum_{i=1}^{n} m_i} (\exp(\lambda) - 1). \tag{9}$$

That is, the asymptotic variance of $\hat{C}$ increases exponentially with the concentration in the PCR. This relationship between the asymptotic variance of $\hat{C}$ and c can also be obtained through the Fisher information of $\hat{C}$ [1].

We note that in the special case where all $m_i = m$, the concentration estimates from individual replicates have identical variances $\mathrm{Var}[\hat{C}_i]$ and their arithmetic mean (Eq. (4)) has asymptotic variance

$$\mathrm{Var}[\hat{C}^{\mathrm{AVG}}] = \mathrm{Var}\left[\frac{1}{n}\sum_{i=1}^{n} \hat{C}_i\right] = \frac{1}{n^2}\left(n \frac{1}{\kappa^2 m} (\exp(\lambda) - 1)\right) = \frac{1}{\kappa^2 \sum_{i=1}^{n} m_i} (\exp(\lambda) - 1), \tag{10}$$

which is identical to the asymptotic variance of the maximum likelihood estimate in Eq. (9).

Using Eqs. (2) and (9) for the mean of $\hat{C}$, the coefficient of variation of $\hat{C}$ can be estimated as

$$\nu_{\hat{C}}(c) = \frac{\sqrt{\mathrm{Var}[\hat{C}]}}{\mathbb{E}[\hat{C}_m]} = \frac{1}{c} \frac{\sqrt{\exp(\lambda) - 1}}{\kappa \sqrt{\sum_{i=1}^{n} m_i}}. \tag{11}$$

B.2 CV with pre-PCR variation

As described in the main text, we account for pre-PCR noise by modeling the concentration in the reaction mixture as a random variable $C_{\mathrm{pre}} = c\, s\, \eta$, where η is a multiplicative noise term that follows a parametric distribution with mean 1 and coefficient of variation $\nu_{\mathrm{pre}} > 0$. Hence, C_{pre} has an expected value of $\mathbb{E}[C_{\mathrm{pre}}] = c\, s$ and coefficient of variation ν_{pre}. The expected number of molecules per partition is then also a random variable $\Theta = C_{\mathrm{pre}}\, v$ with expected value $\mathbb{E}[\Theta] = C_{\mathrm{pre}}\, v = c\, s\, v$ and coefficient of variation ν_{pre}. Under this model, the reported concentration value $\hat{C}$ is still an

unbiased estimator of the true concentration c since

$$\mathbb{E}[\hat{C}|\Theta] = \frac{1}{vs}\mathbb{E}[\hat{\Theta}|\Theta] \overset{\text{Eq. (2)}}{=} \frac{1}{vs}\Theta, \tag{12}$$

where $\hat{\Theta}$ represents the "raw" concentration estimate (before accounting for partition volume and scaling) and

$$\mathbb{E}[\frac{1}{vs}\Theta] = \mathbb{E}[\frac{1}{s}C_{\text{pre}}] = \frac{1}{s}\mathbb{E}[C_{\text{pre}}] = \frac{1}{s}c\,s = c \tag{13}$$

Using the Law of Total Variance, we can express the asymptotic variance of the reported concentration value $\hat{C}$ under pre-PCR noise as

$$\text{Var}[\hat{C}] = \text{Var}[\mathbb{E}[\hat{C}|\Theta]] + \mathbb{E}[\text{Var}[\hat{C}|\Theta]], \tag{14}$$

with the "explained" component of the variance

$$\text{Var}[\mathbb{E}[\hat{C}|\Theta]] \overset{\text{Eq. (12)}}{=} \text{Var}[\frac{1}{vs}\Theta] = \text{Var}[\frac{1}{s}C_{\text{pre}}] = \frac{1}{s^2}\text{Var}[C_{\text{pre}}] = \frac{1}{s^2}(cs)^2\,\nu_{\text{pre}}^2 = c^2\,\nu_{\text{pre}}^2 \tag{15}$$

and the "unexplained" component of the variance

$$\mathbb{E}[\text{Var}[\hat{C}|\Theta]] \overset{\text{Eq. (9)}}{\approx} \mathbb{E}\left[\frac{1}{\kappa^2\sum_{i=1}^{n} m_i}(\exp(\Theta)-1)\right] = \frac{1}{\kappa^2\sum_{i=1}^{n} m_i}(\mathbb{E}[\exp(\Theta)]-1), \tag{16}$$

i. e. we have substituted the constant λ in the expression for the asymptotic variance of $\hat{C}$ in Eq. (9) with the random variable Θ.

B.2.1 Gamma distribution

If we model η as gamma distributed with mean 1 and coefficient of variation ν_{pre}, Θ will also be gamma distributed, with parameters $\alpha = \frac{1}{\nu_{\text{pre}}^2}$ and $\beta = \frac{1}{\lambda\nu_{\text{pre}}^2}$. In this case, an exact solution for the "unexplained" component of the variance can be obtained. That is, by the MGF of the gamma distribution, we know that

$$\mathbb{E}[\exp(\Theta)] = M_{\Theta}(1) = \left(1-\frac{1}{\beta}\right)^{-\alpha} = (1-\lambda\,\nu_{\text{pre}}^2)^{-\frac{1}{\nu_{\text{pre}}^2}}. \tag{17}$$

Substituting back, we get the variance

$$\mathrm{Var}[\hat{C}] = c^2\,\nu_{\mathrm{pre}}^2 + \frac{1}{\kappa^2\sum_{i=1}^n m_i}\left((1-\lambda\,\nu_{\mathrm{pre}}^2)^{-\frac{1}{\nu_{\mathrm{pre}}^2}} - 1\right) \tag{18}$$

and thus

$$\nu_{\hat{C}}(c) = \sqrt{\nu_{\mathrm{pre}}^2 + \frac{1}{c^2\kappa^2\sum_{i=1}^n m_i}\left((1-\lambda\,\nu_{\mathrm{pre}}^2)^{-\frac{1}{\nu_{\mathrm{pre}}^2}} - 1\right)}. \tag{19}$$

B.2.2 Log-normal distribution

We can also model η as log-normally distributed, in which case Θ will also be log-normally distributed with parameters $\hat{\mu} = \log(\lambda) - \frac{\log(1+\nu_{\mathrm{pre}}^2)}{2}$ and $\hat{\sigma}^2 = \log(1+\nu_{\mathrm{pre}}^2)$. While no closed-form solution for the MGF of the log-normal distribution exists, we can use an approximation [3] to obtain

$$\mathbb{E}[\exp(\Theta)] \approx \frac{1}{\sqrt{1+w}}\exp\left(-\frac{(w^2+2\,w)}{2\,\hat{\sigma}^2}\right) = \frac{1}{\sqrt{1+w}}\exp\left(-\frac{(w^2+2\,w)}{2\,\log(1+\nu_{\mathrm{pre}}^2)}\right), \tag{20}$$

where w is an evaluation of the Lambert W function [4] with

$$w = W\left(\hat{\sigma}^2\exp(\hat{\mu})\right) = W\left(\lambda\frac{\log(1+\nu_{\mathrm{pre}}^2)}{\sqrt{1+\nu_{\mathrm{pre}}^2}}\right). \tag{21}$$

B.2.3 Taylor series expansion

If only the mean and coefficient of variation of Θ are known, we can approximate $E[\exp(\Theta)]$ using a Taylor polynomial, i. e.

$$\mathbb{E}[\exp(\Theta)] \approx 1 + \mathbb{E}[\Theta] + \frac{\mathbb{E}[\Theta]^2 + \mathrm{Var}[\Theta]}{2} = 1 + \lambda + \frac{1}{2}\lambda^2(1+\nu_{\mathrm{pre}}^2). \tag{22}$$

Substituting this back into Eq. (14), we obtain

$$\mathrm{Var}[\hat{C}] = c^2\,\nu_{\mathrm{pre}}^2 + \frac{1}{\kappa^2\sum_{i=1}^n m_i}\left(1+\lambda+\frac{1}{2}\lambda^2(1+\nu_{\mathrm{pre}}^2) - 1\right) = c^2\left(\nu_{\mathrm{pre}}^2 + \frac{1}{c\kappa\sum_{i=1}^n m_i} + \frac{1+\nu_{\mathrm{pre}}^2}{2\sum_{i=1}^n m_i}\right) \tag{23}$$

and thus

$$\nu_{\hat{C}}(c) = \sqrt{\nu_{\text{pre}}^2 + \frac{1}{c\kappa \sum_{i=1}^n m_i} + \frac{1+\nu_{\text{pre}}^2}{2\sum_{i=1}^n m_i}}. \tag{24}$$

C Probability of non-detection

C.1 Relationship between probability of non-detection and limit of detection

The Limit of Detection (LoD) of an assay is typically defined as the lowest concentration c_{LOD} at which detection is feasible with sufficient confidence α [5]. If we leave aside the probability of false positive partitions, this corresponds to the complement of the probability of a zero measurement, i. e. $\alpha = 1 - p_{\text{zero}}(c_{\text{LOD}}) = 1 - \exp(-c_{\text{LOD}}\kappa \sum_{i=1}^n m_i)$.

If the specific parameters of an assay are unknown, but the LoD is reported, it may thus be possible to obtain a rough estimate of $\kappa \sum_{i=1}^n m_i$ from the reported LoD via

$$\kappa \sum_{i=1}^n m_i = -log(1-\alpha)/c_{\text{LOD}}. \tag{25}$$

Once an estimate of $\kappa \sum_{i=1}^n m_i$ is available, the probability of non-detection can be computed for arbitrary concentrations in the assay. On the other hand, as the details of the experiment used to establish the LoD may also be unknown and could violate the above simple relationship, we believe it is generally better to jointly estimate the unknown PCR parameters from the provided non-zero measurements, as demonstrated in this work.

C.2 Probability of non-detection with pre-PCR variation

As described in the main text, the probability of non-detection under pre-PCR variation is

$$p_{\text{zero}}(c) = \mathbb{E}_\Theta\left[\Pr(\hat{C} = 0|\Theta = \lambda)\right] = \mathbb{E}[\exp(-\Theta \sum_{i=1}^n m_i)], \tag{26}$$

where, as before, Θ is drawn from a parametric distribution with mean λ and coefficient of variation $\nu_{\text{pre}} > 0$. Moreover, $\mathbb{E}[\exp(-\Theta \sum_{i=1}^n m_i)]$ is again an evaluation of the MGF of Θ. As the Taylor series expansion of Eq. (26) is alternating and its partial sum up to the 2nd degree has a large error, we cannot provide a suitable approximation without further assumptions about Θ. However, if we

assume that Θ is gamma distributed, we can again obtain an exact solution using its MGF, i. e.

$$\mathbb{E}[\exp(-\Theta\sum_{i=1}^{n} m_i)] = M_\Theta(-\sum_{i=1}^{n} m_i) = \left(1 - \frac{-\sum_{i=1}^{n} m_i}{\beta}\right)^{-\alpha} = (1 + \lambda\nu_{\text{pre}}^2 \sum_{i=1}^{n} m_i)^{-\frac{1}{\nu_{\text{pre}}^2}}. \tag{27}$$

If we instead assume that Θ is log-normally distributed, we can use the approximation of its MGF as in Eq. (20), but now with

$$w = W\left(-\lambda\sum_{i=1}^{n} m_i \frac{\log(1+\nu_{\text{pre}}^2)}{\sqrt{1+\nu_{\text{pre}}^2}}\right). \tag{28}$$

D Simulation analysis

In the following section, we provide results of simulation analysis in which we compared our analytical approximations for the coefficient of variation and the probability of non-detection with simulated dPCR measurements. For our simulations, we assumed exemplary PCR parameters based on the Naica Crystal Digital PCR Platform with Sapphire Chips (Stilla Technologies, France), a scaling factor of $s = 30$, a partition volume of $v = 0.519\,\text{nL}$, and $m = 25000$ partitions per measurement, but we also provide a sensitivity analysis of these parameters in Figures S7 and S8. To simulate pre-PCR noise, we randomly drew from a gamma or lognormal distribution with a mean equal to the true concentration and a respective coefficient of pre-PCR variation. Then, the number of positive partitions was simulated under the binomial model described in the main text and transformed into a concentration estimate using the maximum likelihood estimator by Dorazio and Hunter [1], see Eq. (1). The coefficient of variation was empirically estimated from 100 000 simulated concentration values by dividing the sample standard deviation by the sample mean. The probability of non-detection was empirically estimated by the proportion of zero measurements to the total number of simulated measurements.

D.1 Coefficient of variation

Figure S4 shows the coefficient of variation (CV) as a function of the sample concentration under gamma distributed pre-PCR noise. The theoretical prediction for the CV based on Eq. (19) matches the simulated dPCR data well. As can be seen, pre-PCR variation acts as an intercept for the CV function only at higher concentrations, and the CV functions converge as the concentration

approaches zero.

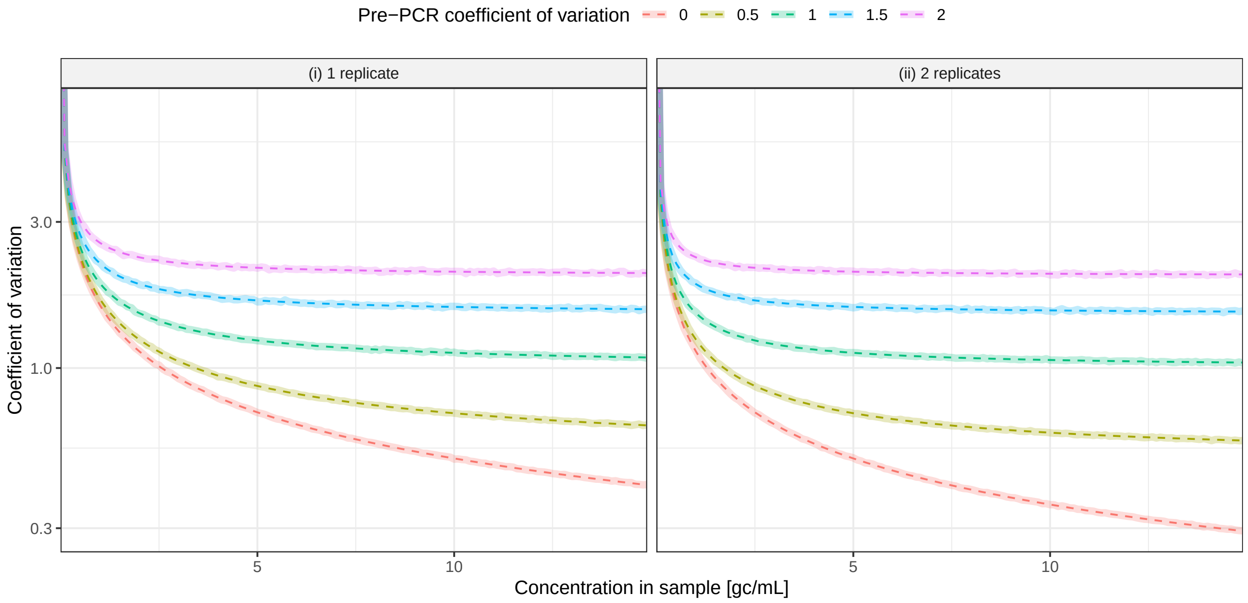

Fig S4. Relationship between concentration and coefficient of variation of dPCR measurements under gamma distributed pre-PCR noise. Shown is the coefficient of variation (CV) of measurements from digital PCR for (i) a single replicate and (ii) the arithmetic mean of two replicates, as a function of the sample concentration c under gamma distributed pre-PCR noise. Solid, wide lines show the CV of simulated measurements for different concentrations in steps of 0.1 gc/mL. Dashed lines show the corresponding CV as predicted by Eq. (19). Colors correspond to different strengths of pre-PCR noise, as measured by the pre-PCR coefficient of variation ν_{pre}.

Figure S5 shows the coefficient of variation (CV) as a function of the sample concentration under log-normally instead of gamma distributed pre-PCR noise. In addition to the CV of simulated measurements, two theoretical approximations are shown. While dashed lines show an approximation of the log-normal MGF by Asmussen et al. [3] (see Supplement B.2.2), dotted lines show the CV as approximated using a Taylor series expansion (see Supplement B.2.3). As can be seen, the approximations yield fairly similar results, however, we find that for high levels of pre-PCR variation, the MGF-based approximation gives slightly lower CV values than the Taylor series-based approximation.

Figure S6 compares the exact solution for gamma distributed pre-PCR noise with the log-normal MGF approximation and the Taylor series-based approximation across a wide range of concentrations. We note that the upper limit of concentrations shown (900 000 gc/mL) corresponds to an extreme case where the expected number of gene copies per partition is 15 or higher (in practice, dPCR assays aim for concentrations that correspond to an expected number of gene copies well below 1,

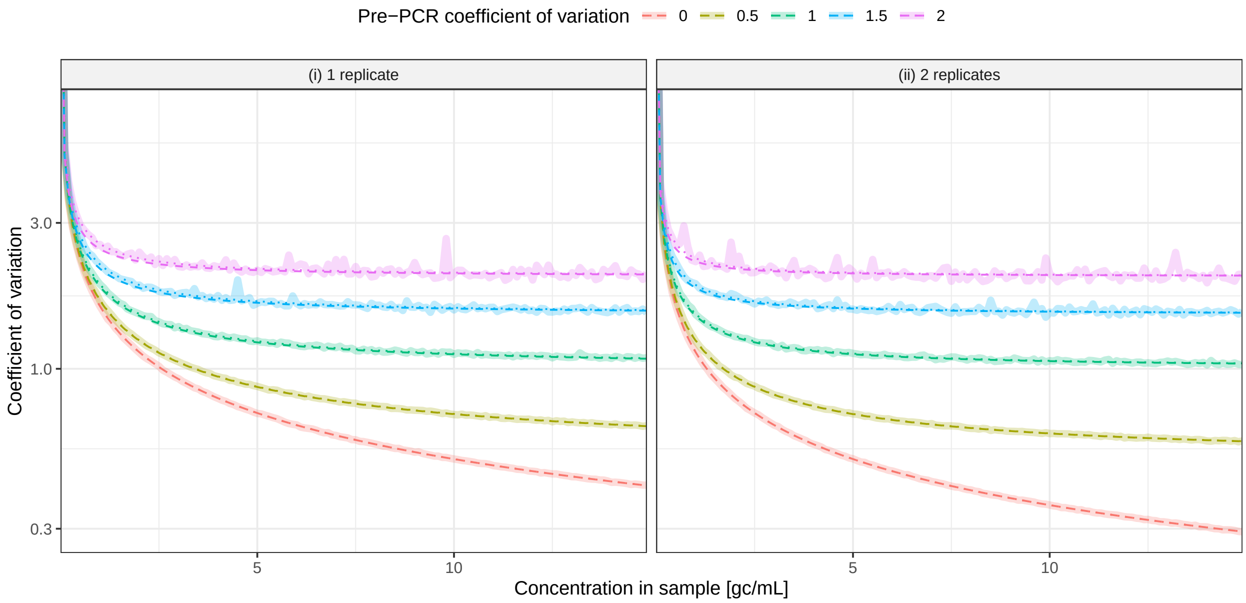

Fig S5. Relationship between concentration and coefficient of variation of dPCR measurements under log-normally distributed pre-PCR noise. Shown is the coefficient of variation (CV) of measurements from digital PCR for (i) a single replicate and (ii) the arithmetic mean of two replicates, as a function of the sample concentration c under log-normally distributed pre-PCR noise. Solid, wide lines show the CV of simulated measurements for different concentrations in steps of 0.1 gc/mL. Dotted lines show the corresponding CV as approximated using a Taylor series expansion, and dashed lines show an alternative approximation of the log-normal MGF by Asmussen et al. [3]. Colors correspond to different strengths of pre-PCR noise, as measured by the pre-PCR coefficient of variation ν_{pre}.

which can be achieved via dilution). As the share of positive partitions in the PCR approaches 1, the CV increases considerably. This increase in CV at extreme concentrations is not captured by the Taylor series expansion approximation but by the exact solution under gamma noise and the MGF approximation under log-normal noise. There are small differences in the CV between the gamma case and the log-normal approximation.

Figure S7 shows a sensitivity analysis of the CV as a function of the sample concentration to the conversion factor κ for the dPCR assay. Compared are results for a conversion factor 10x as large (green), and 10x as small (blue) as assumed in the main analysis (red). The simulated and predicted CV values are based on an assumed pre-PCR coefficient of variation of 0.5. As can be seen, a larger conversion factor reduces the CV, while a smaller conversion factor increases the CV.

Figure S8 shows a sensitivity analysis of the CV as a function of the sample concentration to the total number of valid partitions in the dPCR assay. Compared are results for 15000 partitions less (green), and 15000 partitions more (blue) as assumed in the main analysis (red). The simulated and

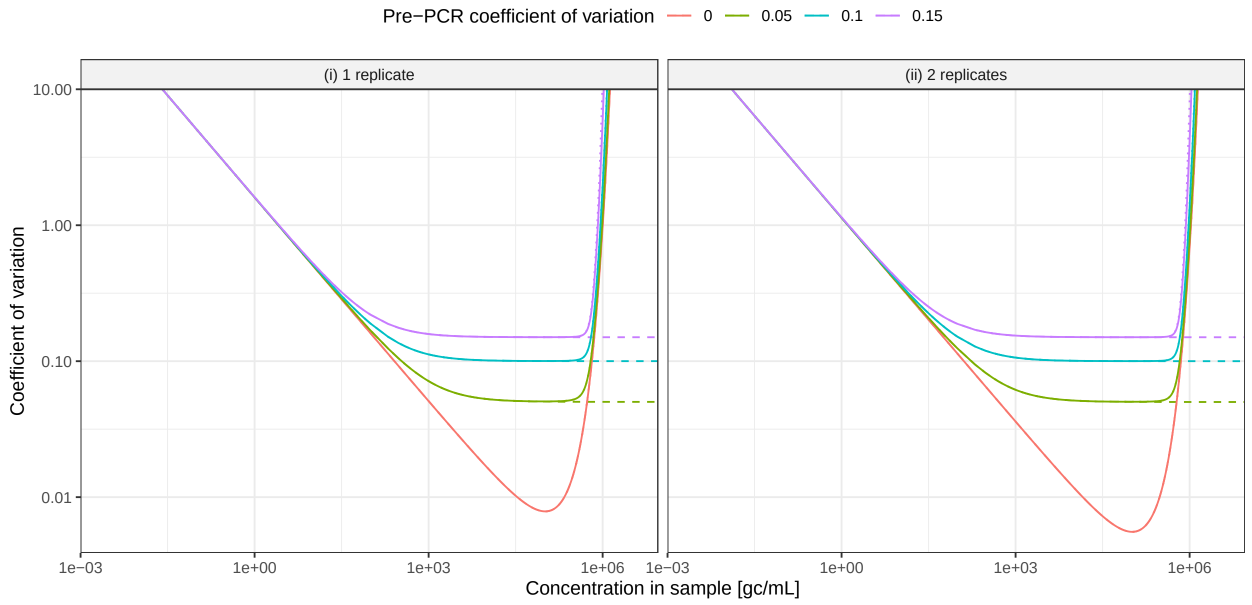

Fig S6. Relationship between concentration and coefficient of variation of dPCR measurements across wide range of concentrations. Shown is the coefficient of variation (CV) of measurements from digital PCR for (i) a single replicate and (ii) the arithmetic mean of two replicates, as a function of the sample concentration c under gamma distributed pre-PCR noise for different concentrations. Solid lines show the CV as predicted under gamma distributed pre-PCR noise, dashed lines show an approximation using a Taylor series expansion, and dotted lines show an approximation under log-normally distributed pre-PCR noise. Colors correspond to different strengths of pre-PCR noise, as measured by the pre-PCR coefficient of variation ν_{pre}.

predicted CV values are based on an assumed pre-PCR coefficient of variation of 0.5. As can be seen, a larger number of partitions reduces the CV, while a smaller number of partitions increases the CV.

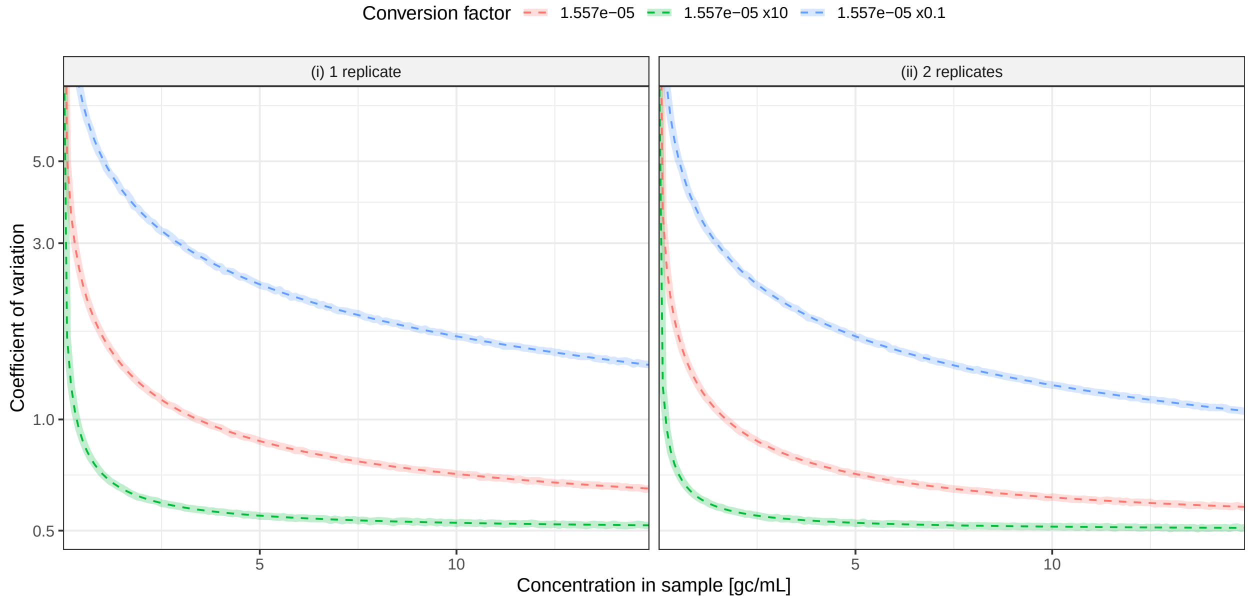

Fig S7. Sensitivity of the coefficient of variation of dPCR measurements to the conversion factor. Shown is the coefficient of variation (CV) of measurements from digital PCR for a conversion factor as in the main analysis ($\kappa = 30 \cdot 0.519\,\mathrm{nL}$, red), 10x as large (green), and 10x as small (blue). The CV is shown for (i) a single replicate and (ii) the arithmetic mean of two replicates, as a function of the sample concentration c under gamma distributed pre-PCR noise with a pre-PCR coefficient of variation of 0.5. Solid, wide lines show the CV of simulated measurements for different concentrations in steps of 0.1 gc/mL, dashed lines show the corresponding CV as predicted by Eq. (19).

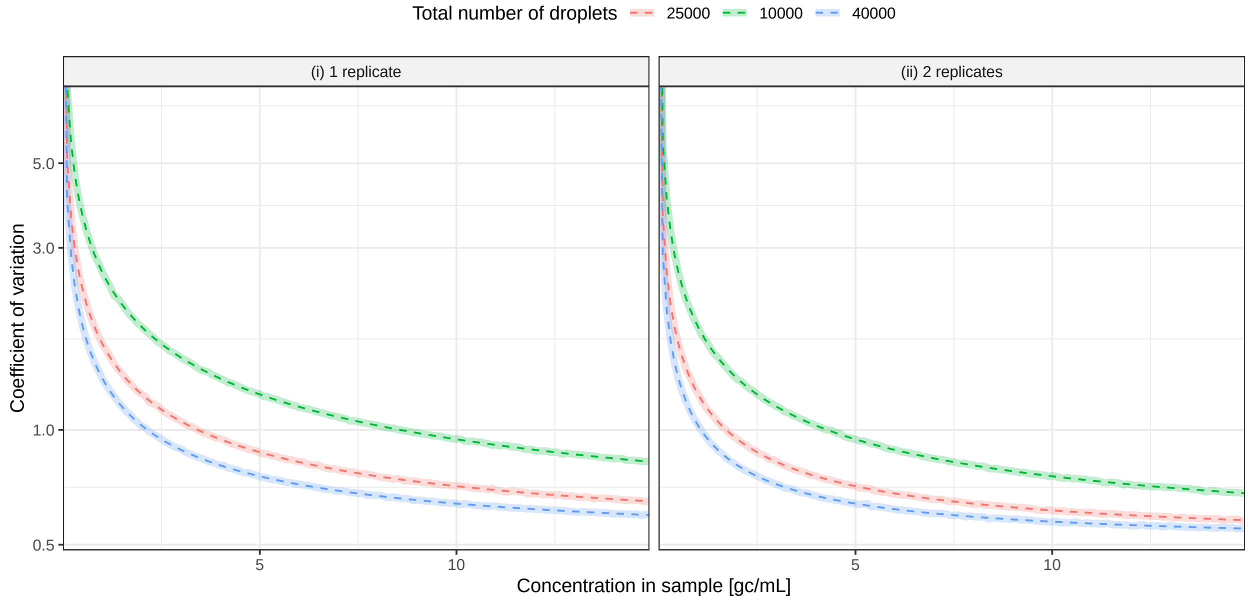

Fig S8. Sensitivity of the coefficient of variation of dPCR measurements to the total number of partitions. Shown is the coefficient of variation (CV) of measurements from digital PCR for 25000 total partitions, i. e. as in the main analysis (red), 10000 total partitions (green), and 40000 total partitions (blue). The CV is shown for i) a single replicate and (ii) the arithmetic mean of two replicates, as a function of the sample concentration c under gamma distributed pre-PCR noise with a pre-PCR coefficient of variation of 0.5. Solid, wide lines show the CV of simulated measurements for different concentrations in steps of 0.1 gc/mL, dashed lines show the corresponding CV as predicted by Eq. (19).

D.2 Probability of non-detection

Figure S9 shows the probability of non-detection as a function of the sample concentration under gamma distributed pre-PCR noise. The presence of pre-PCR noise considerably increases the probability of zero measurements. Moreover, from Eq. (27) we see that the probability of non-detection decreases with the number of partitions and the number of replicates.

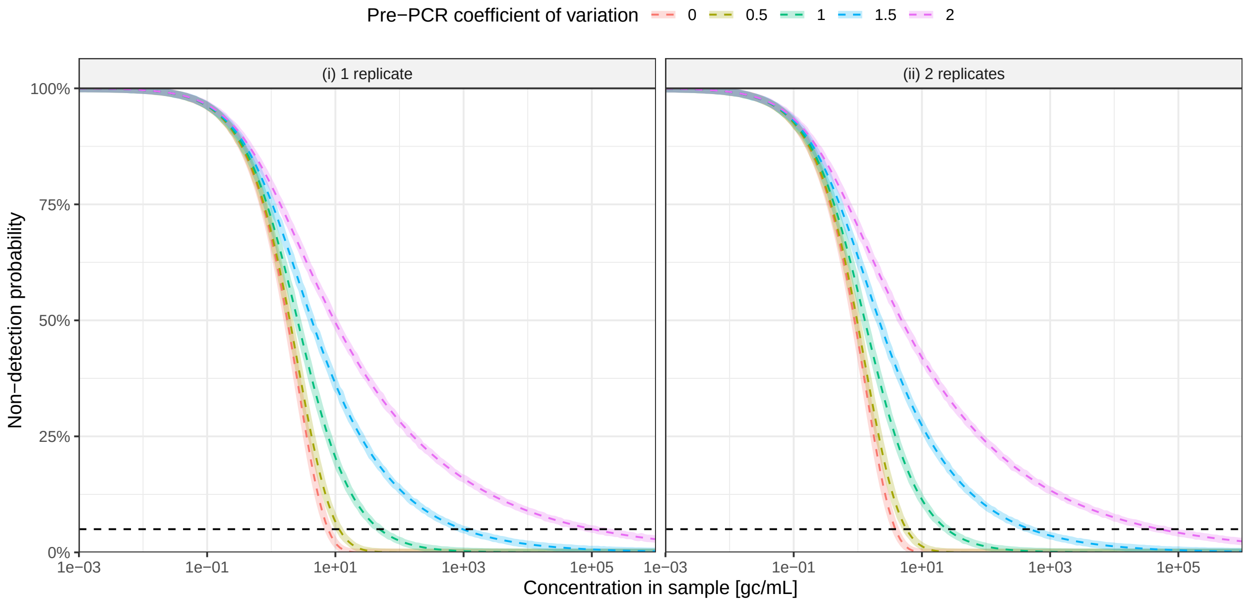

Fig S9. Relationship between concentration and probability of non-detection of dPCR measurements under gamma distributed pre-PCR noise. Shown is the probability of a zero measurement (i. e. non-detection) in digital PCR for (i) a single replicate and (ii) the arithmetic mean of two replicates, as a function of the sample concentration c under gamma distributed pre-PCR noise. Solid, wide lines show the probability of non-detection of simulated measurements for different concentrations in steps of 0.1 gc/µL, while dashed lines show the corresponding probability of non-detection as predicted by Eq. (27). Dashed horizontal lines indicate the limit of detection (LoD), defined as a 5% probability of non-detection. Colors correspond to different strengths of pre-PCR noise, as measured by the pre-PCR coefficient of variation ν_{pre}.

Figure S10 shows the probability of non-detection as a function of the sample concentration under log-normally instead of gamma distributed pre-PCR noise. In addition to the CV of simulated measurements, a theoretical prediction using an approximation of the log-normal MGF by Asmussen et al. [3] (see Supplement C.2) is shown. As can be seen, the approximation matches the simulated CV values well, but slightly underestimates the CV at low concentrations when the pre-PCR variation is very high (Figure S10). Compared to the case of gamma distributed pre-PCR noise (Figure S9), the probability of non-detection falls considerably faster under log-normally distributed pre-PCR noise.

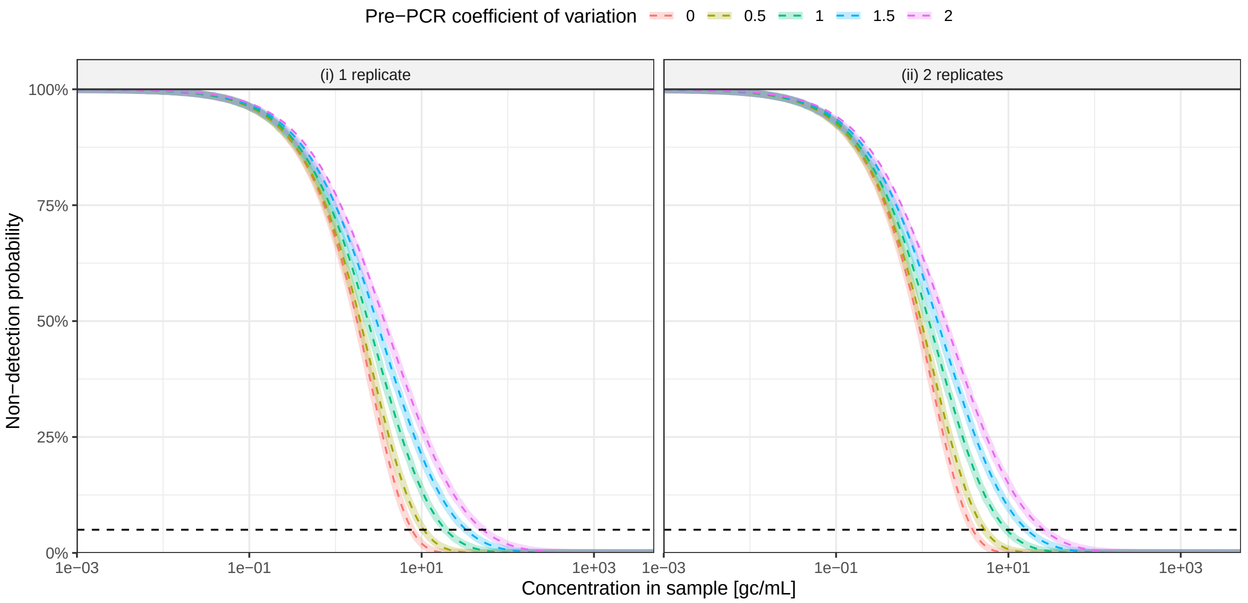

Fig S10. Relationship between concentration and probability of non-detection of dPCR measurements under log-normally distributed pre-PCR noise. Shown is the probability of a zero measurement (i. e. non-detection) in digital PCR for (i) a single replicate and (ii) the arithmetic mean of two replicates, as a function of the sample concentration c under log-normally distributed pre-PCR noise. Solid, wide lines show the probability of non-detection of simulated measurements for different concentrations in steps of 0.1 gc/µL, while dashed lines show the corresponding probability of non-detection as predicted using an approximation of the log-normal MGF by Asmussen et al. [3]. Dashed horizontal lines indicate the limit of detection (LoD), defined as a 5% probability of non-detection. Colors correspond to different strengths of pre-PCR noise, as measured by the pre-PCR coefficient of variation ν_{pre}.

E Comparison with empirical data

In Figure 1 in the main text, we plotted empirical estimates of the CV and probability of non-detection based on real-world dPCR measurements of pathogen concentrations in Swiss wastewater. For this we used data from 3031 autosampler-based, 24-hour-composite samples taken at 14 different municipal treatment plants throughout Switzerland in the time period July 2023 – June 2024. For each sample, viral RNA was extracted from a 40 mL aliquot by vacuum filtration, yielding an elution of 80 µL, and subsequently diluted by a factor of 1:3. As described in Huisman et al. [6] and Nadeau et al. [7], for quantification via dPCR, a 25 µL reaction mix with 5 µL of the template and 20 µL of reagents was run in a droplet-based, sixplex dPCR assay with Influenza A (M gene), Influenza B (M gene), Respiratory Syncytial Virus (N gene), SARS-CoV-2 (N1 and N2 genes), and an internal extraction efficiency control as targets. Quantification was performed using the Naica Crystal Digital PCR System (Stilla® Technologies) with a Sapphire Chip with a maximum number of 30000 droplets

and an average droplet volume of 0.519 nL.

For each target (Influenza A virus, Influenza B virus, Respiratory syncytial virus, and SARS-CoV-2) and sample, the CV was empirically estimated using the sample standard deviation of two technical replicates divided by their sample mean, i. e.

$$\hat{\nu}_{\hat{c}} = \sqrt{\frac{\pi}{2}} \frac{\sqrt{\frac{1}{n-1}\sum_{i=1}^{n}(\hat{c}_i - \mu_{\hat{c}})^2}}{\mu_{\hat{c}}}, \tag{29}$$

where $\mu_{\hat{c}} = \frac{1}{n}\sum_{i=1}^{n}\hat{c}_i$ is the empirical mean of the replicates and $\sqrt{\frac{\pi}{2}}$ is Cureton's bias correction factor for the empirical standard deviation [8]. We note that this correction is based on a normality assumption and therefore becomes increasingly inaccurate at low concentrations. Moreover, in the special case where one of the replicate measurements is zero, we obtain $\hat{\nu}_{\hat{c}} = \sqrt{\pi}$.

To estimate the probability of non-detection, we used the technical replicates by treating the first replicate as an estimate of the true target concentration, and the second replicate as an indicator of the probability of zero. Specifically, for a given window $w \in \{0, \ldots, 30\}$, we selected all replicate pairs where the result of the first replicate was between w and $w+1$ gc/mL. Of these, we then computed the percentage of second replicates with a result of zero to estimate $p_{\text{zero}}(w+0.5)$. The moving window was here used to group samples with a similar concentration together while accounting for the noise of the measurements.

Given the empirical estimates for different target concentrations, we applied locally estimated scatterplot smoothing (LOESS) [9] to estimate a smooth relationship of the target concentration with the CV and probability of non-detection, respectively. Figure S11 shows the comparison between theoretical predictions and the real-world dPCR measurements for Influenza A and B virus, RSV, and SARS-CoV-2.

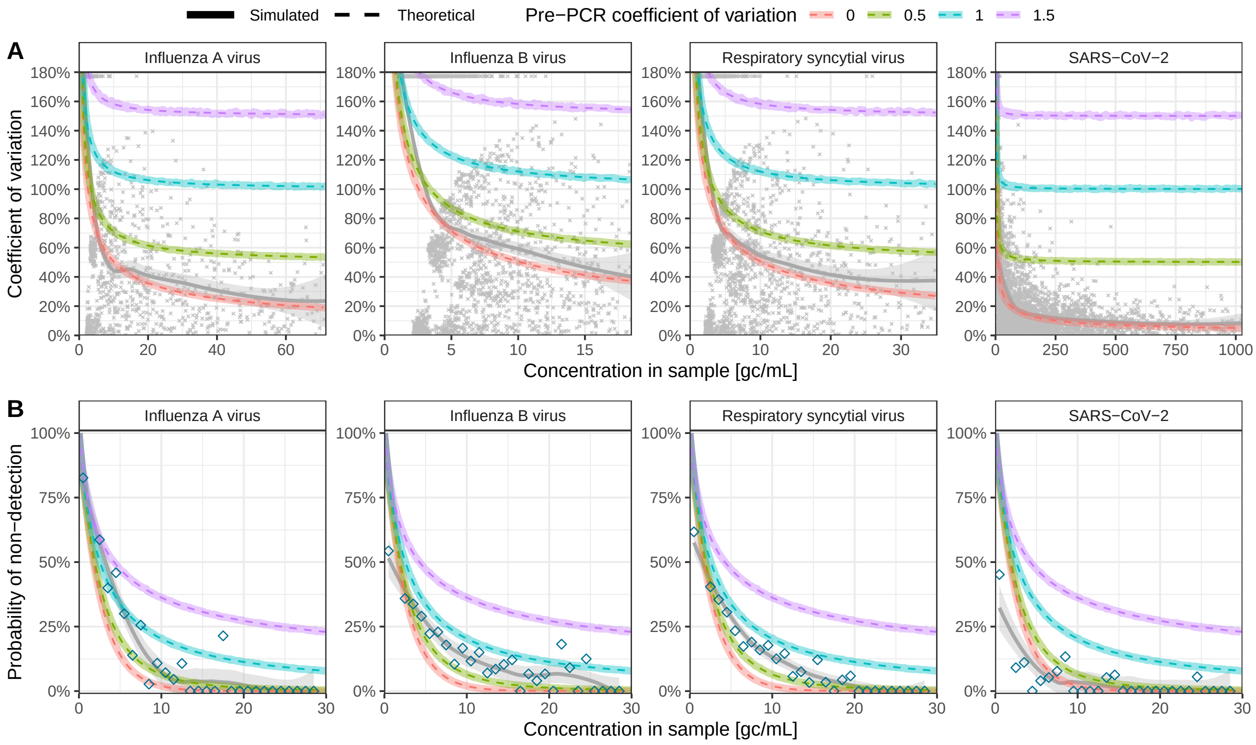

Fig S11. Comparison of theoretical predictions with real-world dPCR measurements. Shown are empirical estimates of (A) the coefficient of variation and (B) the probability of non-detection for dPCR measurements of Influenza A and B virus, RSV, and SARS-CoV-2 concentrations from 14 wastewater treatment plants in Switzerland. Coefficient of variation (CV) estimates are based on the bias-corrected empirical standard deviation of two technical replicates, respectively, divided by their empirical mean (dots). Probability of non-detection estimates are based on the percentage of second replicates with a result of zero among all replicate pairs where the result of the first replicate falls within a moving window of 1 gc/mL width (diamonds). Shown in gray with 95% uncertainty intervals are LOESS-based kernel regressions of the empirical estimates, indicating the relationship between the concentration in the sample and empirical CV and probability of non-detection, respectively. Dashed lines show the CV and probability of non-detection as predicted by the dPCR-specific measurement model, for different strengths of the pre-PCR coefficient of variation ν_{pre} under gamma distributed pre-PCR noise. Solid, wide lines show estimates based on simulated dPCR measurements, respectively. Note that as the empirical CV is based on technical replicates of the same sample, respectively, it does not include variation from pre-PCR noise.

F Likelihoods for non-zero dPCR measurements

F.1 Conditional distribution of non-zero measurements

In the proposed hurdle model, $f_{\text{PCR}}(x)$ is the distribution of measurements conditioned on a non-zero result. While the overall distribution of measurements has mean c and coefficient of variation $\nu_{\hat{C}}(c)$, this is not true for the conditional distribution. Instead, using the law of total expectation, i. e.

$$\mathbb{E}\left[\hat{C}\right] = \mathbb{E}\left[\hat{C}\middle|\hat{C}=0\right]\cdot P(\hat{C}=0) + \mathbb{E}\left[\hat{C}\middle|\hat{C}>0\right]\cdot P(\hat{C}>0), \tag{30}$$

we know that

$$\mathbb{E}\left[\hat{C}\middle|\hat{C}>0\right] = \frac{\mathbb{E}\left[\hat{C}\right]}{P(\hat{C}>0)} = \frac{\mathbb{E}\left[\hat{C}\right]}{1-p_{\text{zero}}(c)} = \frac{c}{1-p_{\text{zero}}(c)}. \tag{31}$$

That is, the conditional mean increases relative to the unconditional mean as the probability of non-detection becomes large. Moreover, by the same argument, we see that

$$\mathbb{E}\left[\hat{C}^2\middle|\hat{C}>0\right] = \frac{\mathbb{E}\left[\hat{C}^2\right]}{P(\hat{C}>0)} = \frac{\mathbb{E}\left[\hat{C}^2\right]}{1-p_{\text{zero}}(c)}. \tag{32}$$

Expanding the conditional variance and inserting the above, we get

$$\text{Var}\left[\hat{C}\middle|\hat{C}>0\right] = \mathbb{E}\left[\hat{C}^2\middle|\hat{C}>0\right] - \mathbb{E}\left[\hat{C}\middle|\hat{C}>0\right]^2 = \frac{\mathbb{E}\left[\hat{C}^2\right]}{1-p_{\text{zero}}(c)} - \frac{\mathbb{E}\left[\hat{C}\right]^2}{(1-p_{\text{zero}}(c))^2}. \tag{33}$$

Then, using $\mathbb{E}\left[\hat{C}^2\right] = \text{Var}\left[\hat{C}\right] + \mathbb{E}\left[\hat{C}\right]^2$, we obtain

$$\text{Var}\left[\hat{C}\middle|\hat{C}>0\right] = \frac{\text{Var}\left[\hat{C}\right] + \mathbb{E}\left[\hat{C}\right]^2}{1-p_{\text{zero}}(c)} - \frac{\mathbb{E}\left[\hat{C}\right]^2}{(1-p_{\text{zero}}(c))^2} = \frac{Var\left[\hat{C}\right]}{1-p_{\text{zero}}(c)} - \mathbb{E}\left[\hat{C}\right]^2 \frac{p_{\text{zero}}(c)}{(1-p_{\text{zero}}(c))^2}. \tag{34}$$

From the conditional variance, we can derive the conditional coefficient of variation as

$$\text{CV}[\hat{C}|\hat{C}>0] = \sqrt{\frac{\text{Var}\left[\hat{C}\middle|\hat{C}>0\right]}{\mathbb{E}\left[\hat{C}\middle|\hat{C}>0\right]^2}} \tag{35}$$

$$= \sqrt{\left(\frac{\mathrm{Var}\left[\hat{C}\right]}{1 - p_{\mathrm{zero}}(c)} - \mathbb{E}\left[\hat{C}\right]^2 \frac{p_{\mathrm{zero}}(c)}{(1 - p_{\mathrm{zero}}(c))^2} \right) \frac{(1 - p_{\mathrm{zero}}(c))^2}{\mathbb{E}\left[\hat{C}\right]^2}} \tag{36}$$

$$= \sqrt{\frac{\mathrm{Var}\left[\hat{C}\right]}{\mathbb{E}\left[\hat{C}\right]^2}(1 - p_{\mathrm{zero}}(c)) - p_{\mathrm{zero}}(c)} \tag{37}$$

$$= \sqrt{\nu_{\hat{C}}(c)^2 (1 - p_{\mathrm{zero}}(c)) - p_{\mathrm{zero}}(c)}. \tag{38}$$

Thus, the conditional CV decreases relative to the unconditional CV as the probability of non-detection becomes large. The resulting relationship between the unconditional and conditional mean and CV is also visualized in Figure S12.

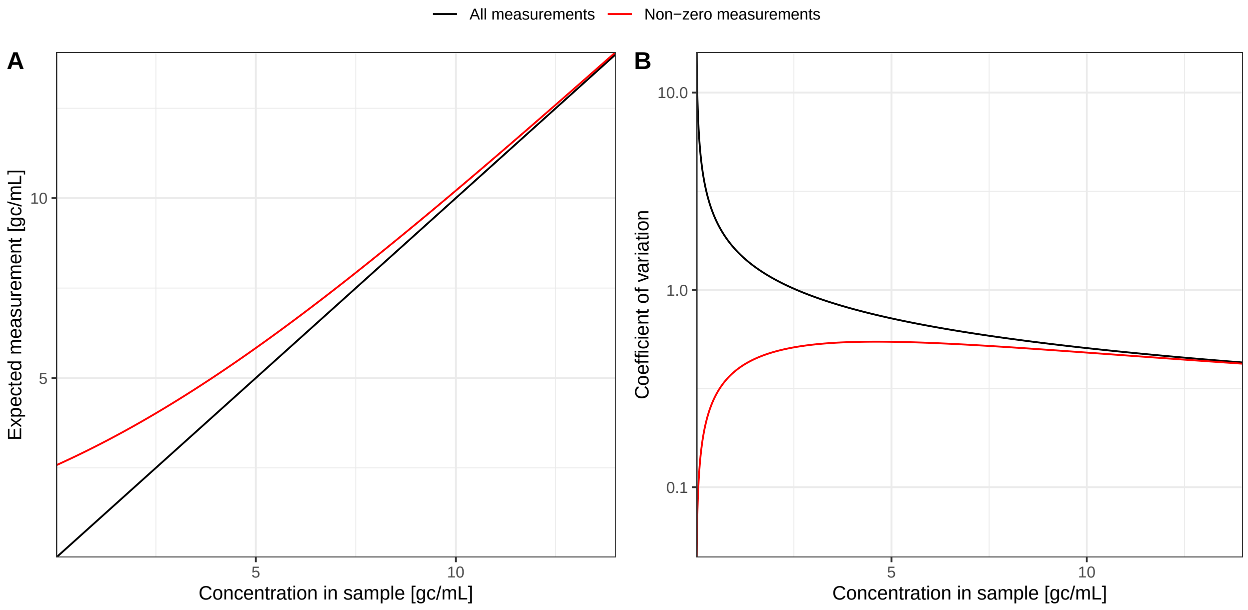

Fig S12. Effect of conditioning on non-zero measurements on the mean and coefficient of variation of measurements. Shown are the (A) mean and (B) coefficient of variation of the unconditional (black, all measurements) and conditional (red, non-zero measurements) distribution of measurements as a function of the true concentration in the sample. The differences between the unconditional and conditional distribution increase as the concentration becomes small.

F.2 Back-computation of binomial likelihood

If the total number of partitions, the partition volume, and the scaling factor of each measurement are known exactly, the number of positive partitions in the PCR can be back-calculated from the

concentration estimate $\hat{c}$ as

$$y = \sum_{i=1}^{n} m_i(1 - \exp(-\hat{c}\kappa)). \tag{39}$$

Given these quantities, it is also possible to fit a Binomial distribution to the observed y according to Eq (1) from the main text. However, this approach only works well if the specific parameters of the PCR are known exactly. Otherwise, the use of a Binomial likelihood has two important drawbacks. First, and most importantly, when using a Binomial distribution, any misspecification or uncertainty about the PCR parameters directly influences the expected concentration estimate, not only its variance. Thus, a lack of knowledge about the PCR parameters can bias concentration estimates even if the reported concentration value $\hat{C}$ is unbiased. Second, in contrast to our continuous likelihood approach, if only $\hat{c}$ is known exactly, then the back-computation of y depends on the assumed PCR parameters m, s, and v, which makes a joint estimation of these parameters from dPCR data difficult.

F.3 Comparison of continuous measurement distributions

In this work, we use a continuous distribution to model non-zero reported concentration values. Figure S13 compares the cumulative distribution functions (CDFs) of different continuous probability distributions to the discrete CDF of reported concentrations implied by the binomial distribution of the underlying positive partition counts. As can be seen, the normal distribution is an inappropriate approximation as it assigns non-zero probability to negative measurements. Since zero measurements are represented by our hurdle model, we use a strictly positive distribution to represent non-zero measurements (see Figure S13A). We here compared the log-normal, inverse Gaussian, and gamma distributions, each parameterized by their mean and coefficient of variation. While the log-normal distribution is often used as a default to model positive measurements, we note that the concentration estimates $\hat{c}$ are a log-linear transform of the binomially (and therefore approximately normally) distributed partition counts. The log-normal distribution, however, would fit well for an exponential transform. Indeed, we find that a gamma distribution offers a better fit than the log-normal or inverse Gaussian distribution, especially at larger concentrations (see Figure S13B), as indicated by their discretized Kullback-Leibler divergence to true distribution ($\text{KL}_{\text{Gamma}} = 0.01$, $\text{KL}_{\text{Log-normal}} = 0.05$, $\text{KL}_{\text{Inverse Gaussian}} = 0.06$). Other continuous positive distributions, such as the truncated normal

distribution, could also provide a suitable approximation, but are difficult to parameterize by their mean and coefficient of variation. We therefore use a gamma distribution to model non-zero measurements.

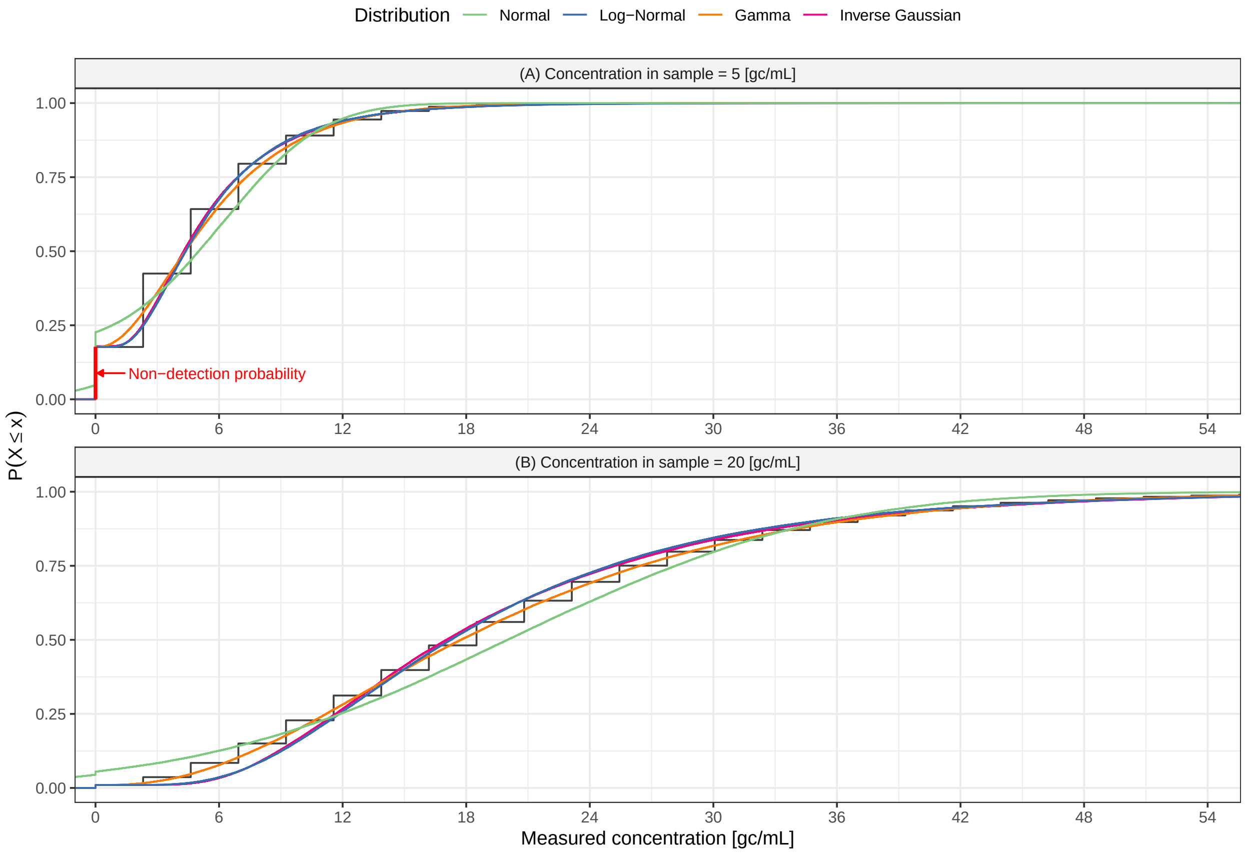

Fig S13. Continuous distributions approximating the distribution of dPCR measurements. Shown in black is the discrete cumulative distribution function of dPCR measurements obtained from the number of positive partitions in the assay for a concentration of (A) 5 gc/mL and (B) 20 gc/mL, assuming $m = 25\,000$ partitions, a conversion factor of $\kappa = 1.73 \times 10^{-5}$ and no pre-PCR noise. This distribution can be approximated using a hurdle model with a probability of non-detection as defined in Eq. (26) and continuously distributed non-negative measurements with conditional mean $\mathbb{E}[\hat{C}|\hat{C} > 0]$ and conditional coefficient of variation $\mathrm{CV}[\hat{C}|\hat{C} > 0]$. Shown in colors are the continuous cumulative distribution functions of a hurdle model using a normal (green), log-normal (blue), gamma (orange), and inverse Gaussian (pink) distribution for the non-negative measurements, respectively.

F.4 Continuous approximation to the binomial likelihood

By modeling reported concentration values as continuously distributed using a gamma distribution for non-zero measurements, the true distribution of possible concentration values is only approximated. Figure S14 shows a histogram-based comparison of the approximate dPCR likelihood with the true probabilities for observations under a binomial model. While the overall shape of the true distribution

is matched, we find systematic differences in the bin probabilities when the true concentration is low but the probability of non-zero measurements is not negligible (Figure S16). In contrast, when concentrations are extremely low, most of the probability mass is correctly attributed to non-detection via the hurdle model. Overall, this highlights that the fit of the gamma distribution to the discrete concentration estimates based on the underlying binomially distributed positive partition counts is limited, especially when the probability mass is distributed only over a small set of partition counts.

We also assessed the fit of the approximation under pre-PCR noise. Figure S15 shows a histogram-based comparison of the approximate dPCR likelihood (using our approximations for the probability of non-detection and CV of measurements under gamma distributed pre-PCR noise) with the true probabilities for observations under a binomial model (obtained using numerical marginalization over the multiplicative noise term η via quadrature over a uniform grid with 10 000 points). Overall, the deviation of our approximate likelihood from the binomial model did not significantly increase under pre-PCR noise and was even slightly smaller at high concentrations (Figure S16)

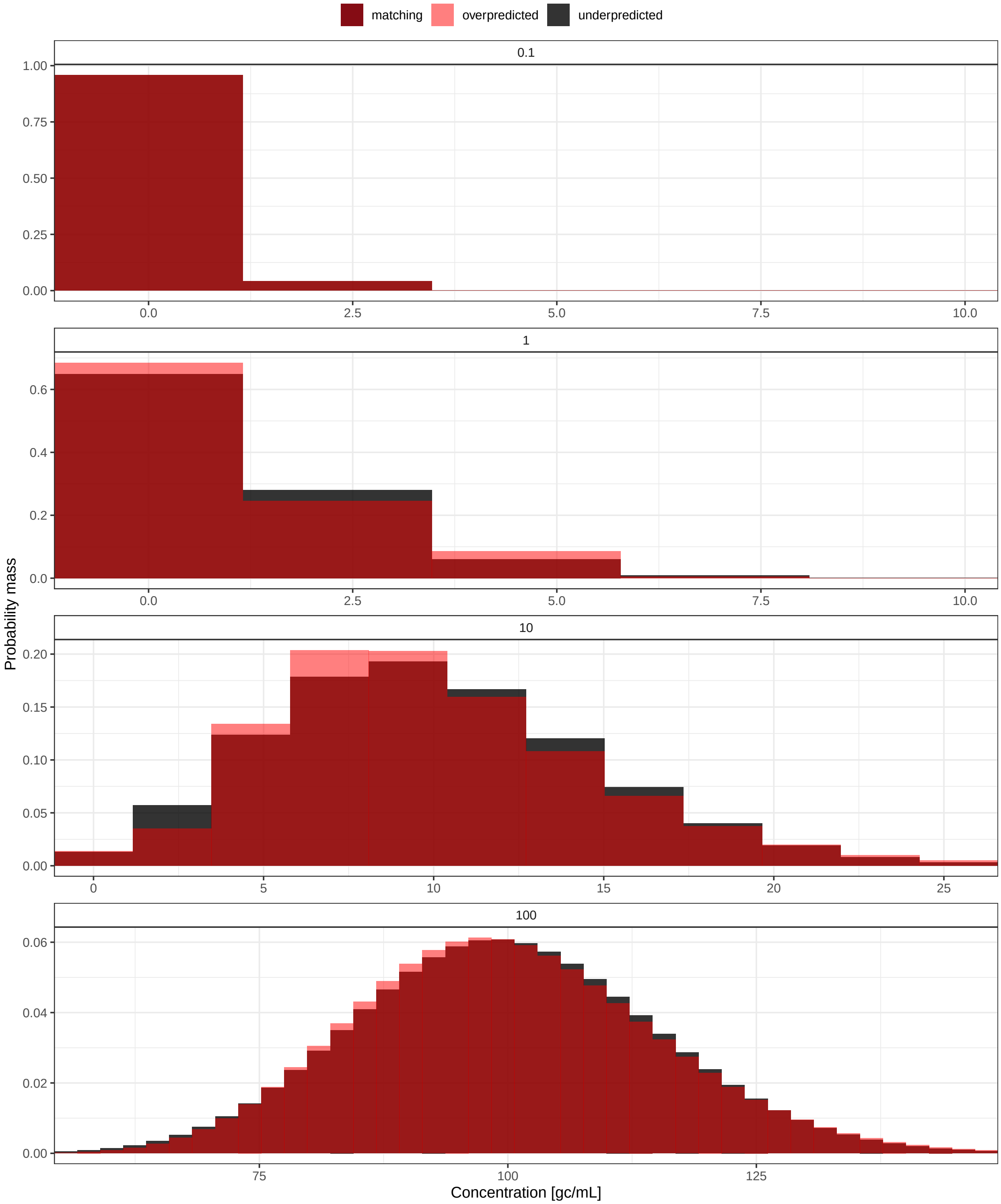

Fig S14. Comparison of the approximate dPCR likelihood and the binomial likelihood. Shown are likelihood histograms for a concentration of 0.1, 1, 10, and 100 gc per mL, based on a scaling factor of $s = 30$, a partition volume of $v = 0.519\,\text{nL}$, and $m = 25000$ partitions per measurement. Bins were centered on the discrete concentration estimates that can be obtained under Eq. (1). Bin probabilities from the continuous dPCR likelihood approximation are shown in red, with the probabilities of the gold-standard binomial model contrasted in grey.

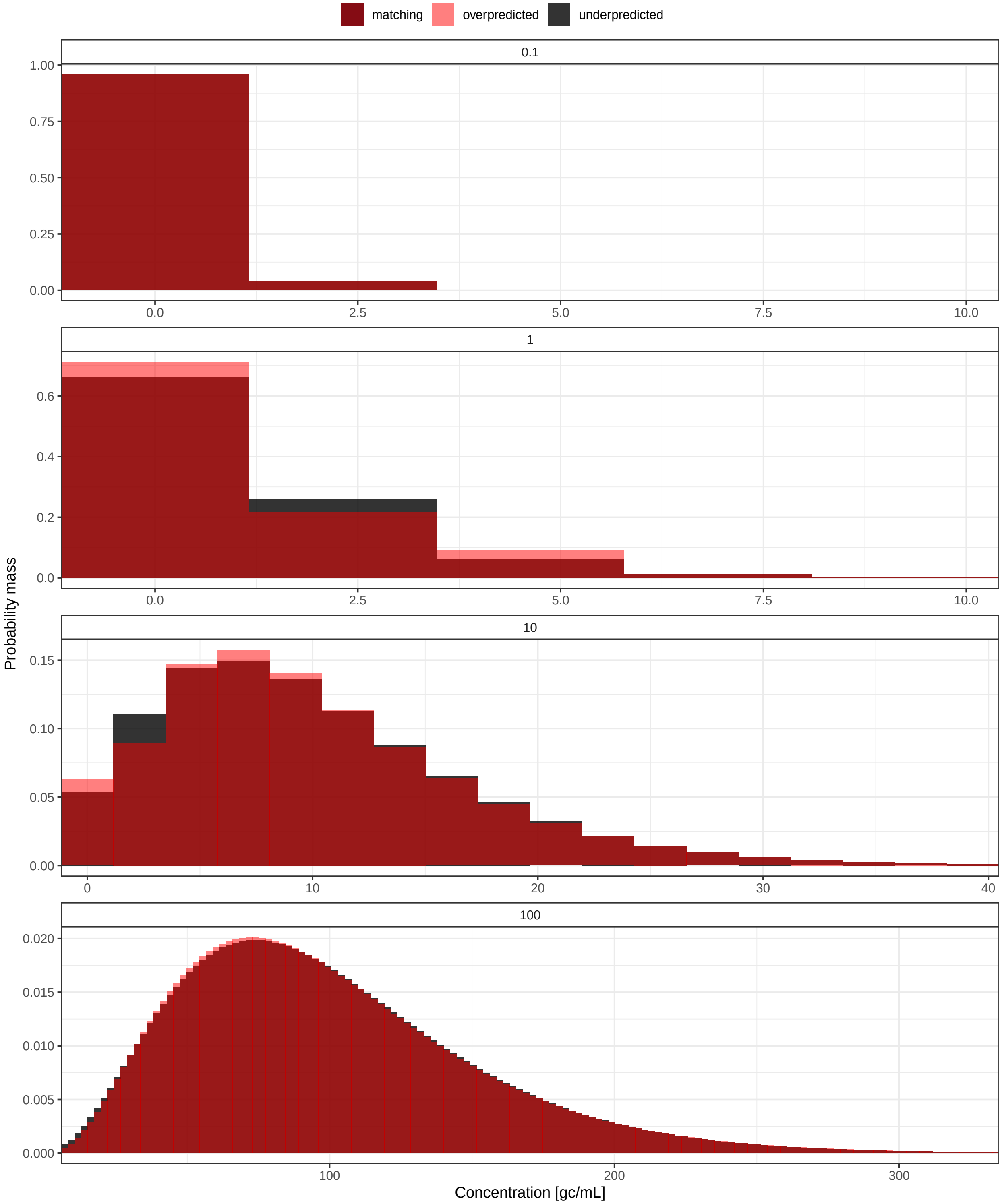

Fig S15. Comparison of the approximate dPCR likelihood and the binomial likelihood under pre-PCR noise. Shown are likelihood histograms for a concentration of 0.1, 1, 10, and 100 gc per mL and gamma distributed pre-PCR noise with coefficient of variation $\nu_{\text{pre}} = 0.5$, based on a scaling factor of $s = 30$, a partition volume of $v = 0.519\,\text{nL}$, and $m = 25000$ partitions per measurement. Bins were centered on the discrete concentration estimates that can be obtained under Eq. (1). Bin probabilities from the continuous dPCR likelihood approximation are shown in red. Probabilities of the gold-standard binomial model were computed using numerical integration over the multiplicative pre-PCR noise factor η and are contrasted in grey.

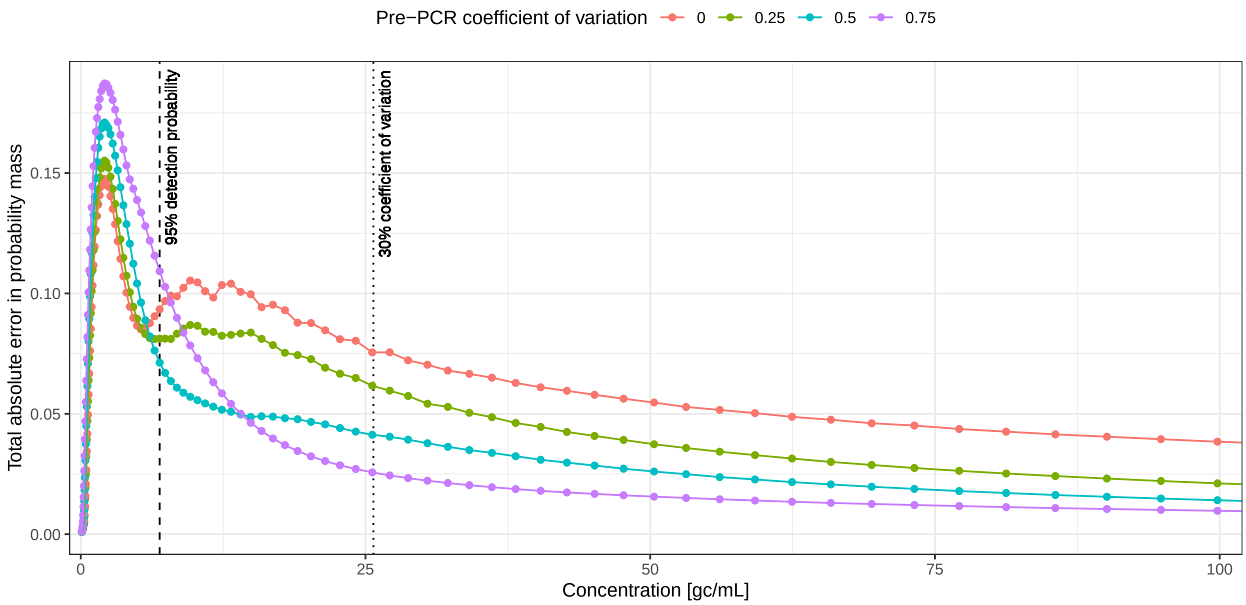

Fig S16. Deviation of the approximate dPCR likelihood from the binomial likelihood. Shown is the total absolute error in histogram bins between the continuous dPCR likelihood approximation and the gold-standard binomial model across a range of target concentrations (0.1–100 gc per mL) under different strengths of pre-PCR noise. Bins were centered on the discrete concentration estimates that can be obtained under Eq. (1). Likelihoods were computed assuming a scaling factor of $s = 30$, a partition volume of $v = 0.519\,\mathrm{nL}$, and $m = 25000$ partitions per measurement. Probabilities under the binomial model with pre-PCR noise were obtained using numerical marginalization over the multiplicative noise term η via quadrature over a uniform grid with 10 000 points.

F.5 Identifiability of c and κ

For a set of measurements from a sample with fixed concentration c, the CV of concentration estimates (Eq. (24)) and the probability of non-detection (Eq. (26)) provide no information about c on their own, because c always appears as the product with the conversion factor κ. This is expected, since the per-partition probability of detection in digital PCR depends on the concentration in the reaction mixture rather than the concentration in the original sample. Instead, in our likelihood, c becomes identifiable by combining Eqs. (24) and (26) with Eq. (31), i. e. the expected concentration estimate conditional on detection. Unless c is so small that $\mathbb{E}[C|C>0]$ is dominated by the probability of non-detection, c is therefore well identified through the likelihood. Thus, in turn, unless c is very high (such that Eqs. (24) and (26) become nearly constant in κ) the parameter κ is also identifiable, although this is not a primary aim of our analysis. This is illustrated further in Figure S17, which shows likelihood surfaces averaged over all possible observations $\hat{c}$ under the data-generating model. In each case, the surface is maximized near the true values of c and κ.

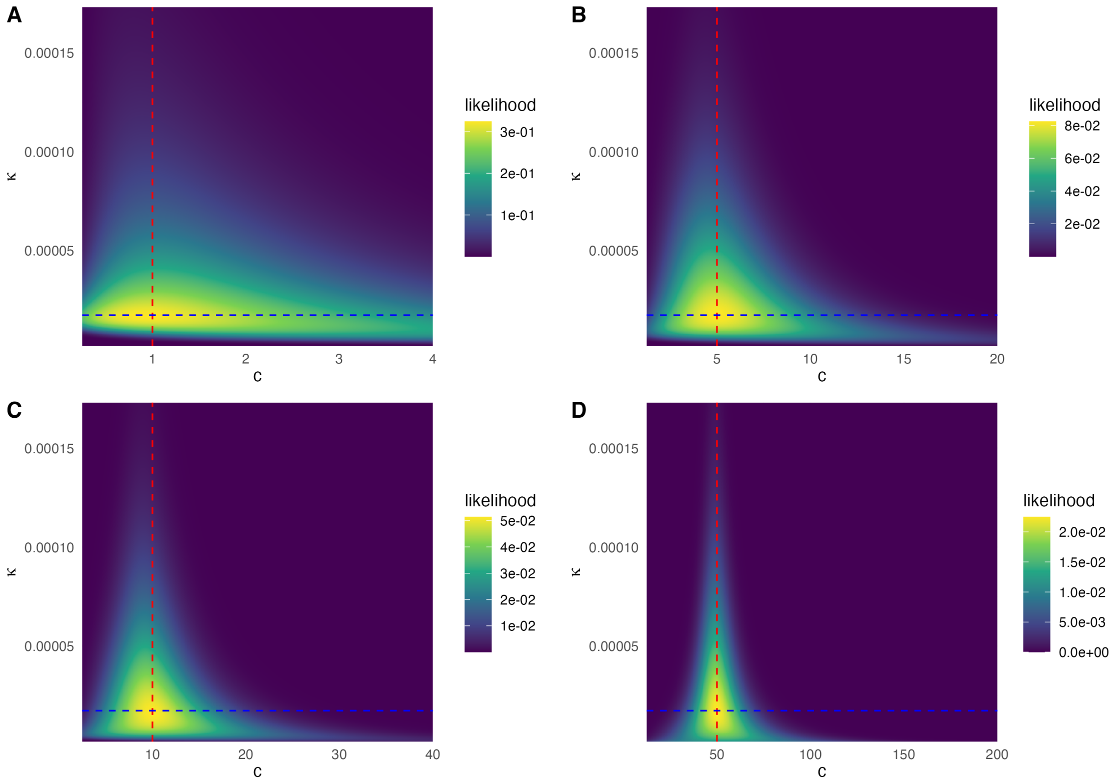

Fig S17. Likelihood surfaces for c and κ. Shown is the likelihood surface for c and κ, averaged over all possible observations $\hat{c}$ under the binomial model, for different true sample concentrations of (A) $c=1$, (B) $c=5$, (C) $c=10$, and (D) $c=50$. Vertical and horizontal lines indicate the assumed true values of c and κ, with κ fixed at 1.73×10^{-5}.

The identifiability analysis above assumes a fixed, known concentration c. When the shared dPCR-related parameters $m_{\max}$, μ_δ, σ_δ, κ, and ν_{pre} are inferred jointly from measurements at multiple unknown concentrations c_j, identifiability requires that the concentrations themselves are constrained by a structural model, such as a generalized linear model (GLM) relating concentrations to measured covariates, so that the residual variation can be attributed to the measurement error parameters. This assumption underlies the validation exercises in this work. Crucially, if the structural concentration model is misspecified, the shared dPCR-related parameters may absorb unexplained variation and their estimates will be biased. The same caution applies when combining data from different sources. For example, treating protocol-specific parameters such as κ or ν_{pre} as global constants could introduce systematic bias. We therefore recommend estimating these parameters on a per-lab/per-protocol basis when compiling and analyzing data from several sources.

G Inference from dPCR measurements

G.1 Priors for assay parameters

G.1.1 Number of total partitions

To estimate the total number of valid partitions across technical replicates $\sum_{i=1}^{n} m_i$, we explicitly sample the individual m_i. This allows us to take into account that the valid number of partitions varies between PCR runs due to random loss of partitions. We thus define $m_i = m_{\max}(1-\delta_i)$, with the maximum number of partitions $m_{\max}$ and the relative partition loss δ_i for technical replicate i. As can be seen in Figure S18, the proportion of lost partitions per PCR run can be modeled as approximately logit-normal distributed. Thus, we parameterize $\frac{\delta_i}{\delta_{\max}} \sim \text{Logit-normal}(\mu_\delta, \sigma_\delta)$, where $\delta_{\max} \in [0,1]$ is a fixed maximum partition loss and μ_δ and σ_δ are the location and scale parameters of the Logit-Normal distribution, respectively. We use $\delta_{\max}$ to account for potential quality control in the lab, whereby dPCR runs are discarded if they have fewer than a certain number of valid partitions. We here used a default value of $\delta_{\max} = 50\%$. For the other parameters, i. e. $m_{\max}$, μ_δ, and σ_δ, we used (truncated) normal priors (see Supplements H.3, I and J for detailed priors). Note that while the m_i are nuisance parameters that are not individually identifiable, they are drawn from the same underlying distribution with shared parameters $m_{\max}$, μ_δ, and σ_δ.

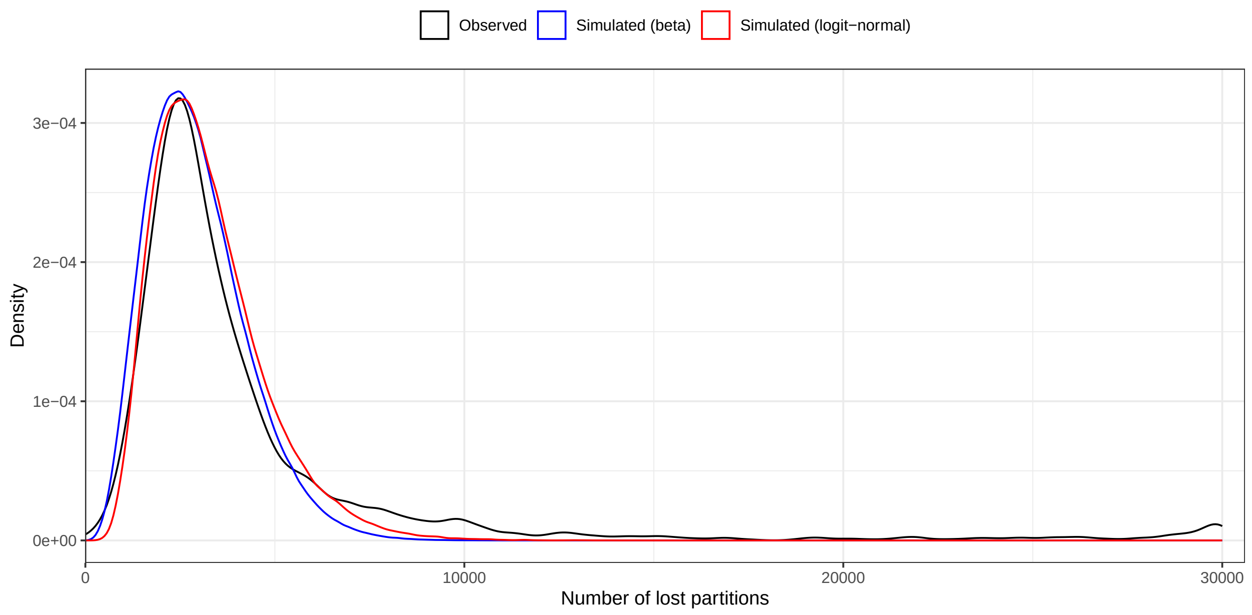

Fig S18. Distribution of lost partitions in a dPCR assay. Shown in black is a kernel density estimate for the distribution of the number of lost partitions in a dPCR assay based on 2733 runs on a Stilla® Sapphire chip with a maximum of 30000 partitions. The observed density was matched using a beta (blue) and logit-normal (red) distribution.

G.1.2 Conversion factor

Since the scaling factor s and partition volume v are not individually identifiable, we specify a prior for the "conversion factor" factor $\kappa = sv$. This factor expresses how many expected gene copies per partition a concentration of 1 gc/mL in the original sample corresponds to. We used a broad truncated normal prior for κ (see Supplements H.3, I and J for detailed priors).

G.1.3 Pre-PCR coefficient of variation

The pre-PCR coefficient of variation ν_{pre} describes all unexplained variation before the PCR step, including variation from extraction and preprocessing, but also from sampling and environmental processes not included in the model. We used a broad truncated normal prior for ν_{pre} (see Supplements H.3, I and J for detailed priors).

G.2 Estimation

The dPCR-specific likelihood was implemented as a function in the probabilistic programming language stan [10]. We integrated this likelihood function in a validation model for estimating a single concentration from replicate measurements, a generalized linear model (GLM) for estimating the association of covariates with the target concentration, and in the EpiSewer wastewater model for

estimating reproduction numbers and other transmission parameters from wastewater measurements over time. For comparison purposes, we also implemented a normal and log-normal likelihood for concentration measurements, as well as a binomial model for the number of positive partitions. Figure S19 shows an overview over the hierarchical structure of the generalized linear model, including priors. Importantly, the parameters defining the characteristics of the assay (the conversion factor κ, the pre-PCR coefficient of variation ν_{pre}, the maximum number of partitions m_{max}, and the logit mean and standard deviation of the relative partitions μ_δ and σ_δ) are shared across samples.

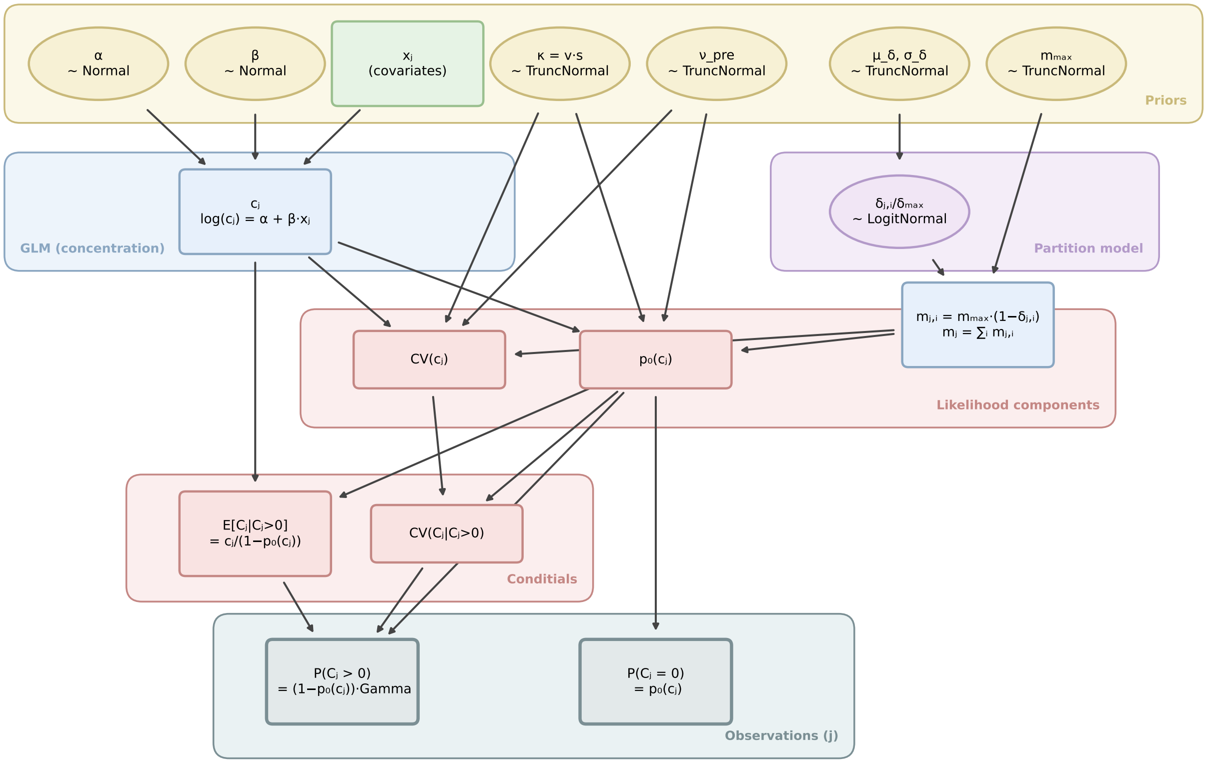

Fig S19. Overview of the GLM model structure with dPCR-specific likelihood. The concentration c_j in sample j is modeled via a linear regression model (here shown with logarithmic link function). The coefficient of variation and probability of non-detection of the dPCR measurement of sample j depend on the sample concentration c_j as well as the parameters of the assay (conversion factor κ, pre-PCR coefficient of variation ν_{pre} and the total number of valid partitions m_j, summed across all technical replicates of this sample). Together with the true concentration, the coefficient of variation and probability of non-detection define the parameters of a zero-augmented Gamma likelihood for the reported concentrations.

We fitted models via Markov chain Monte Carlo (MCMC) using cmdstan version 2.34.1 via cmdstanr version 0.8.1 [11]. We ran four No-U-Turn sampler chains with 1,000 warm-up and 1,000 sampling iterations each, with a maximum tree depth of 15, an initial step size of 0.01,

and an adaptation target acceptance statistic of 0.99. We checked the fitted models for low effective sample sizes (ESS < 400) [12], and for convergence problems using the number of divergent transitions [13], the Bayesian fraction of missing information (E-BFMI < 0.2) [14] and the Gelman-Rubin convergence diagnostic ($\hat{R} > 1.01$) [15]. The checks indicated sufficient effective sampling sizes and good convergence and mixing of chains.

H Validation of inference

H.1 Posterior distribution under approximate likelihood

To assess the accuracy of inference under a continuous likelihood approximation, we compared the posterior distributions for the true concentration c obtained under the approximate dPCR likelihood and the binomial likelihood, assuming a uniform prior $c \sim \mathcal{U}_{[0,10\,000]}$. Figure S20 shows example posterior distributions obtained from 10 simulated replicate dPCR measurements for different levels of the target concentration. We find that at low concentrations, the posterior distribution under the approximate likelihood can notably differ from that under the true model, but these differences are moderate compared to the overall uncertainty of the posterior estimates under limited sample size. We computed the mean of the posterior distribution under the approximate dPCR likelihood as the number of observations becomes large, and found a negative bias at low concentrations. Specifically, for concentrations with over 95% detection probability but substantial relative measurement noise (coefficient of variation of concentration estimates over 30%), we observed a negative bias of up to -1% of the true concentration. For extremely low concentrations, the bias was arbitrarily large in relative terms (see Figure S21) but remained limited in absolute terms (not larger than -0.13 gc per mL for the conditions assessed, see Figure S22). For larger concentrations, the continuous likelihood approximation becomes increasingly accurate, and the bias of the posterior tends to zero.

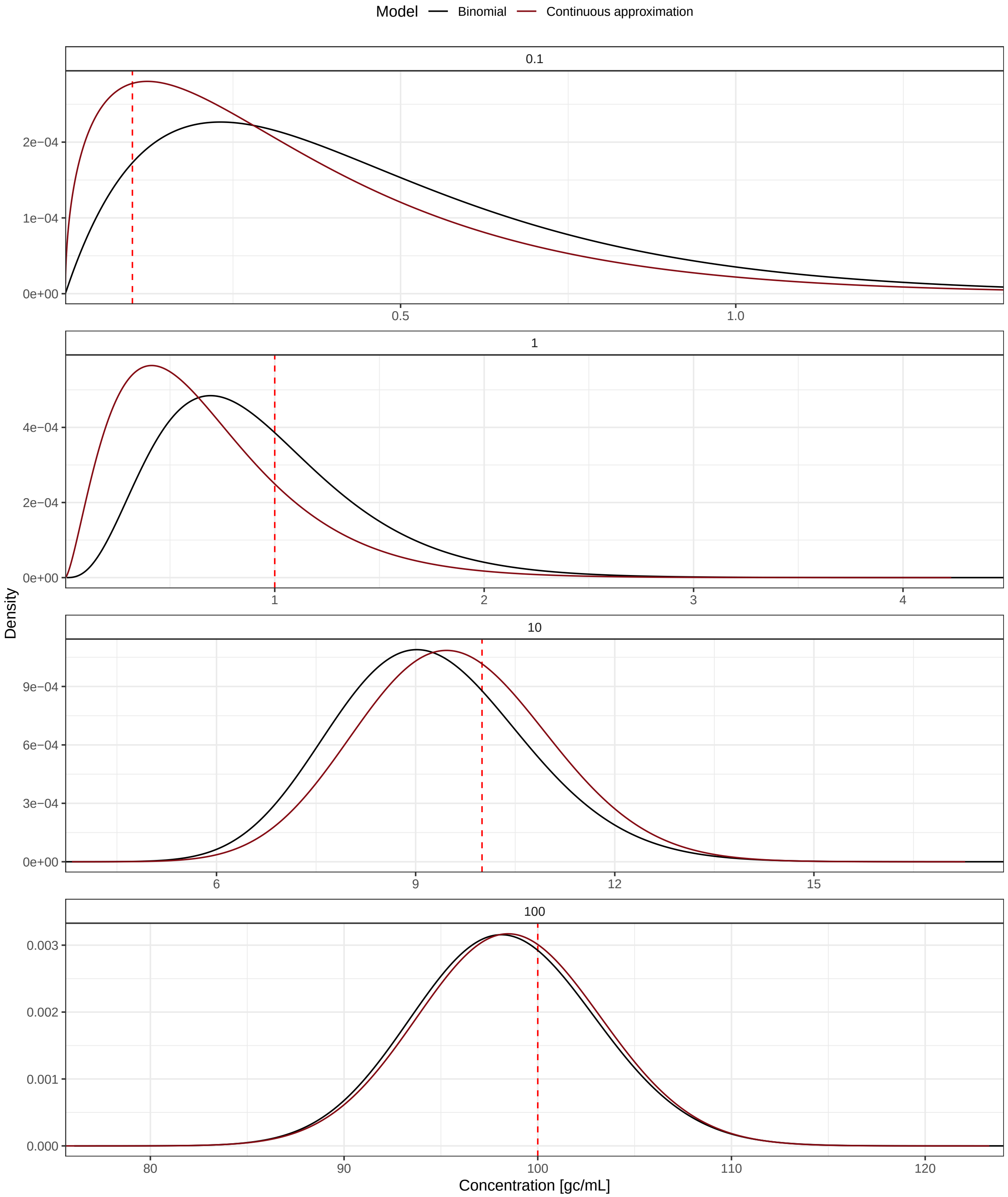

Fig S20. Example posterior distributions for the target concentration under the binomial and approximate dPCR likelihood. Posterior distribution for the true concentration c based on 10 simulated replicate dPCR measurements, obtained using numerical integration of the gold-standard binomial likelihood (black) and the continuous dPCR likelihood approximation (red). Measurements were simulated for a concentration of 0.1, 1, 10, and 100 gc per mL, based on a scaling factor of $s = 30$, a partition volume of $v = 0.519\,\text{nL}$, and $m = 25000$ partitions per measurement. A uniform prior $c \sim \mathcal{U}_{[0,10\,000]}$ was assumed.

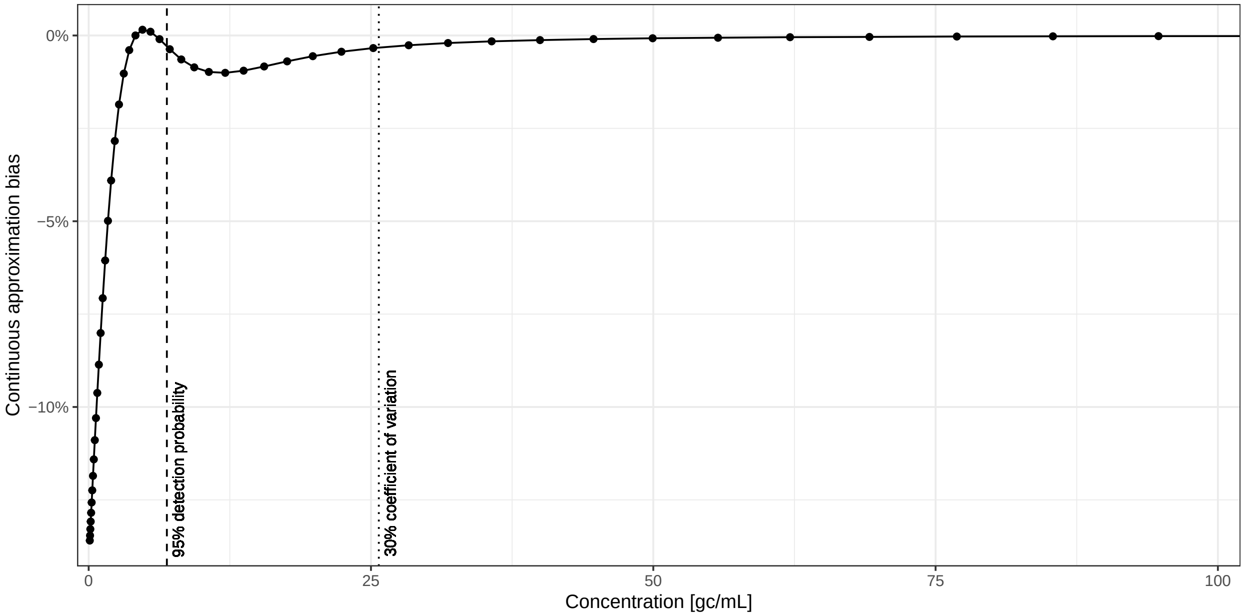

Fig S21. Relative bias of the posterior distribution for the target concentration of the approximate dPCR likelihood. The bias of the posterior distribution for the true concentration c under the continuous dPCR likelihood approximation was estimated using numerical integration across a range of target concentrations (0.1–100 gc per mL). Bias was computed as the relative deviation of the posterior mean from the true concentration as the number of replicate measurements $n \to \infty$, assuming a scaling factor of $s = 30$, a partition volume of $v = 0.519\,\text{nL}$, and $m = 25000$ partitions per measurement. A uniform prior $c \sim \mathcal{U}_{[0,10\,000]}$ was assumed.

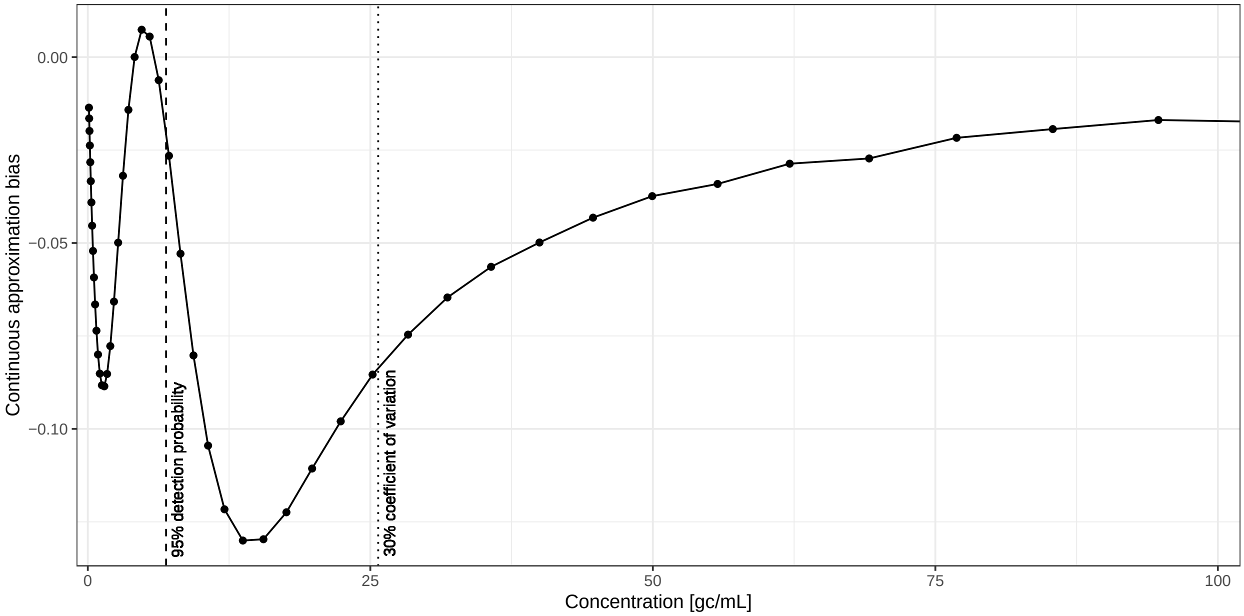

Fig S22. Absolute bias of the posterior distribution for the target concentration of the approximate dPCR likelihood. The bias of the posterior distribution for the true concentration c under the continuous dPCR likelihood approximation was estimated using numerical integration across a range of target concentrations (0.1–100 gc per mL). Bias was computed as the difference between the posterior mean and the true concentration as the number of replicate measurements $n \to \infty$, assuming a scaling factor of $s = 30$, a partition volume of $v = 0.519\,\mathrm{nL}$, and $m = 25000$ partitions per measurement. A uniform prior $c \sim \mathcal{U}_{[0,10\,000]}$ was assumed.

H.2 Estimating a single concentration

We tested inference via the dPCR-specific likelihood by fitting the model for a single concentration to simulated dPCR measurements with different true sample concentrations (1, 3, and 5 gc/mL). For comparison purposes, we also tested the model with two simpler likelihoods, i. e. i) using a normal distribution with constant variance and ii) using a log-normal distribution with constant CV. When using the log-normal likelihood, zero measurements had to be dropped from the analysis, otherwise, the models and data used were identical for all likelihoods. We deliberately used a large number of 100 simulated replicates per concentration for estimation to study asymptotic differences between the different likelihoods.

Figure S23 shows the true concentration and estimated concentrations using the dPCR-specific as well as the normal and log-normal likelihood function, together with the posterior predictive distributions for the dPCR measurements. When using a dPCR-specific likelihood, the probability of zero measurements is explicitly represented and the true concentration is estimated with little bias. When using a normal likelihood, estimates of the concentration show little bias too, however, the model can predict negative measurements. When using a log-normal likelihood, no negative measurements are predicted but zero measurements must be dropped from the analysis. This leads to a significant bias in the estimated concentration when the true concentration is small.

H.3 Estimating coefficients of a regression model

To test our model in a regression setting, we simulated concentrations using a log-linear model, i. e.

$$log(c) = \alpha + \beta x, \tag{40}$$

with intercept α and slope β. We used this model to simulate sample concentrations c_j at different points $x \in \{0, \dots, 9\}$. For each sample, we simulated pre-PCR noise (with coefficient of variation sampled from a truncated normal distribution with location 0 and scale 0.5) and subsequent quantification via dPCR, i.e. partitions counts and corresponding concentration estimates based on two technical replicates. Here we assumed a fixed conversion factor of 1.73×10^{-5}, a maximum of 30 000 partitions, and, for each replicate, a binomially distributed number of lost partitions (loss probability given by a scaled logit-normal distribution with parameters $\mu = -2$ and $\sigma = 0.4$ and a

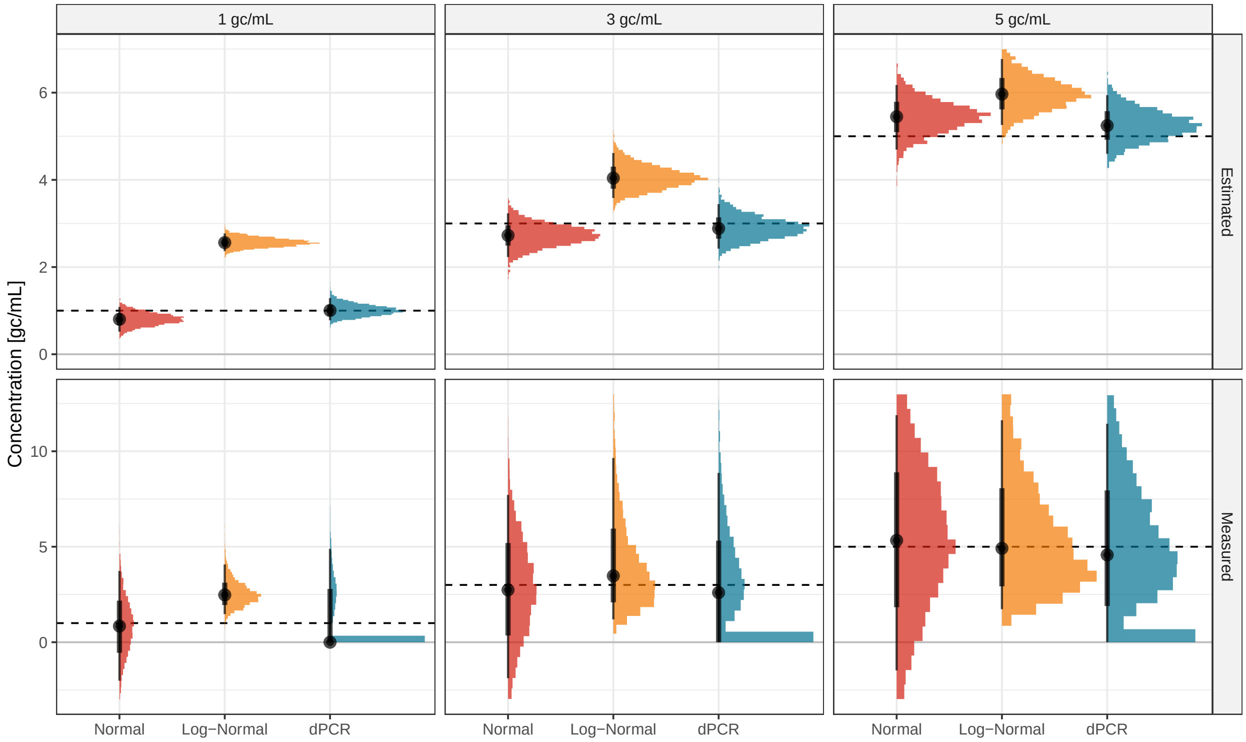

Fig S23. Comparison of estimated concentration and predicted dPCR measurements under different likelihood functions. Shown is the posterior distribution of the estimated true concentration (top) and the posterior predictive distribution of measurements (bottom) from a Bayesian model fitted with a normal (red), log-normal (orange), and dPCR-specific (blue) likelihood to simulated dPCR measurements of different concentrations (1, 3, and 5 gc/mL). Dashed lines show the true concentration, respectively.

scaling factor of 0.5, i. e. the maximum relative partition loss). We used this procedure to simulate concentration measurements for different slopes β (ranging between -0.5 and 0.5 in steps of 0.025).

We then fitted our dPCR-specific GLM to the simulated measurements, using broad priors for the assay parameters as described in Table S1. For comparison, we also fitted a normal and log-normal likelihood to the measurements. For the log-normal likelihood, we placed a half-normal prior with a standard deviation of 0.5 on the coefficient of variation. For the normal likelihood, we assumed a fixed variance, but parameterized it in terms of the average coefficient of variation to use the same noise prior as in the log-normal model. Finally, we also fitted a binomial model to the corresponding simulated partition counts. Here we assumed exact knowledge of true assay parameters described above.

For all models, we provided a normal prior with a mean of 0 and a standard deviation of 0.5 for α and β, respectively.

To assess the quality of our continuous likelihood approximation at large concentrations, we also

Parameter	Description	Prior	Details
m_{max}	maximum number of partitions	$\text{Normal}^{+}(\mu = 30000, \sigma = 10000)$	accommodates dPCR systems with 10 000 to 50 000 partitions
δ_{max}	maximum relative partition loss	$\delta_{\text{max}} = 0.5$	fixed to default of 50%
μ_{δ}	scaled logit-level expected relative partition loss	$\text{Normal}(\mu = -1.7, \sigma = 1.3)$	for $\delta_{\text{max}} = 0.5$, this corresponds to an average loss of 1–30%
σ_{δ}	scaled logit-level standard deviation of partition loss	$\text{Normal}^{+}(\mu = 1.2, \sigma = 0.5)$	$\sigma_{\delta} \approx [0.5, 2]$
κ	concentration conversion factor	$\text{Normal}^{+}\left(\mu = 1 \times 10^{-5}, \sigma = 5 \times 10^{-6}\right)$	$\kappa \ll 1 \times 10^{-4}$
ν_{pre}	pre-PCR coefficient of variation	$\text{Normal}^{+}(\mu = 0, \sigma = 0.5)$	weakly informative prior, less than 100% variation

Table S1. Overview of priors for the dPCR-specific model used for validation. Normal^{+} denotes a normal distribution truncated at zero.

simulated measurements from the same log-linear model as before, however with increasingly large intercepts. For this, we fixed the slope coefficient to 0.2 and varied the intercept corresponding to an expected share of positive partitions in the assay between 10% and 90% at steps of 1%. We then fitted the dPCR-specific, normal, and log-normal likelihood GLM to the simulated concentration measurements as well as the binomial likelihood GLM to the simulated partition counts, using the same priors as before, except for a broader prior for the intercept (normal prior with a mean of 0 and standard deviation of 10).

Figures S24 and S25 show the estimated slope coefficients and intercepts for the different shares of positive partitions in the assay. We find that the continuous approximations achieve good coverage of the true slope and intercept at high concentrations, although estimates under the normal approximation were more uncertain and variable. We found no systematic decrease in coverage or increase in error as the expected share of positive partitions increased, except for extreme concentrations that approach the saturation of the assay (Figures S26 and S27).

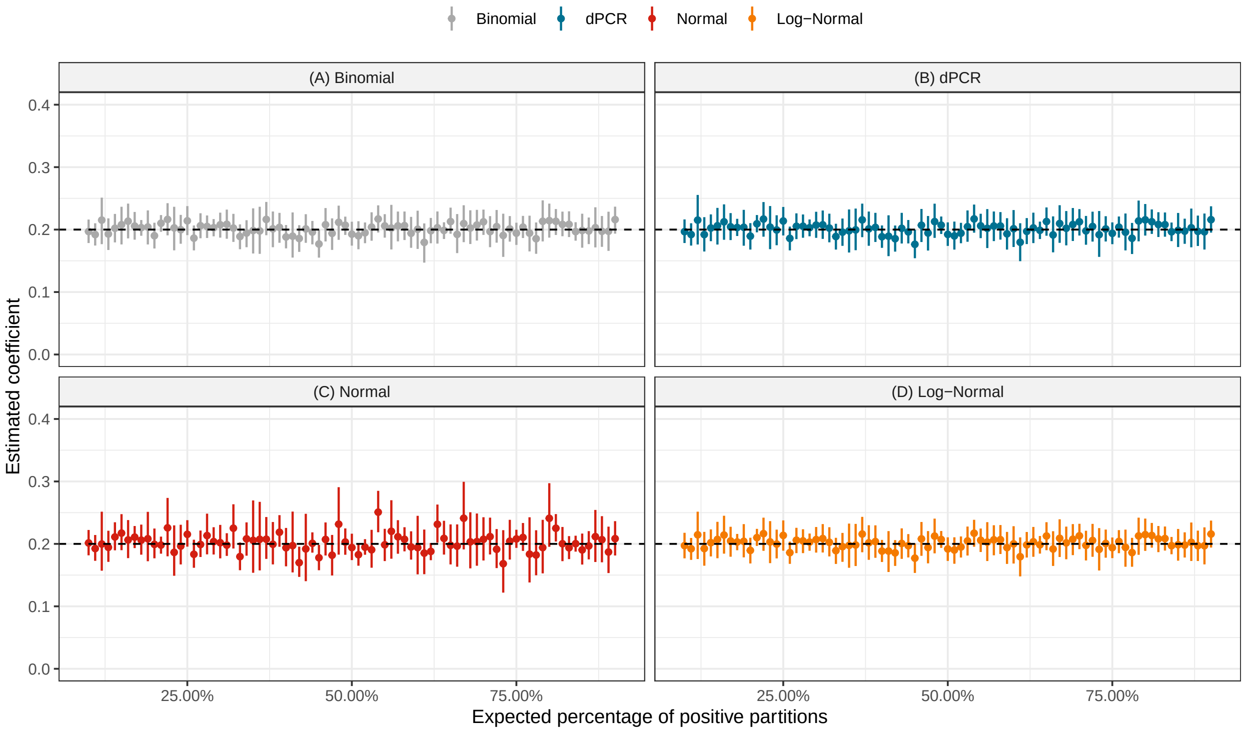

Fig S24. Estimation of a regression coefficient from simulated dPCR measurements at high concentrations. Partition counts and measured concentrations from dPCR were simulated using log-linear regression models with a coefficient of 0.2 and different intercepts (corresponding to an increasing average share of positive partitions in the dPCR). For each simulated data set, the slope coefficient was estimated using 10 simulated dPCR measurements, i. e. (A) with observed partition counts under a binomial model and (B–D) without partition counts (i. e. only reported concentrations) under a dPCR-specific, normal, and log-normal model. Shown are median estimates (dots) with 95% credible intervals (bars).

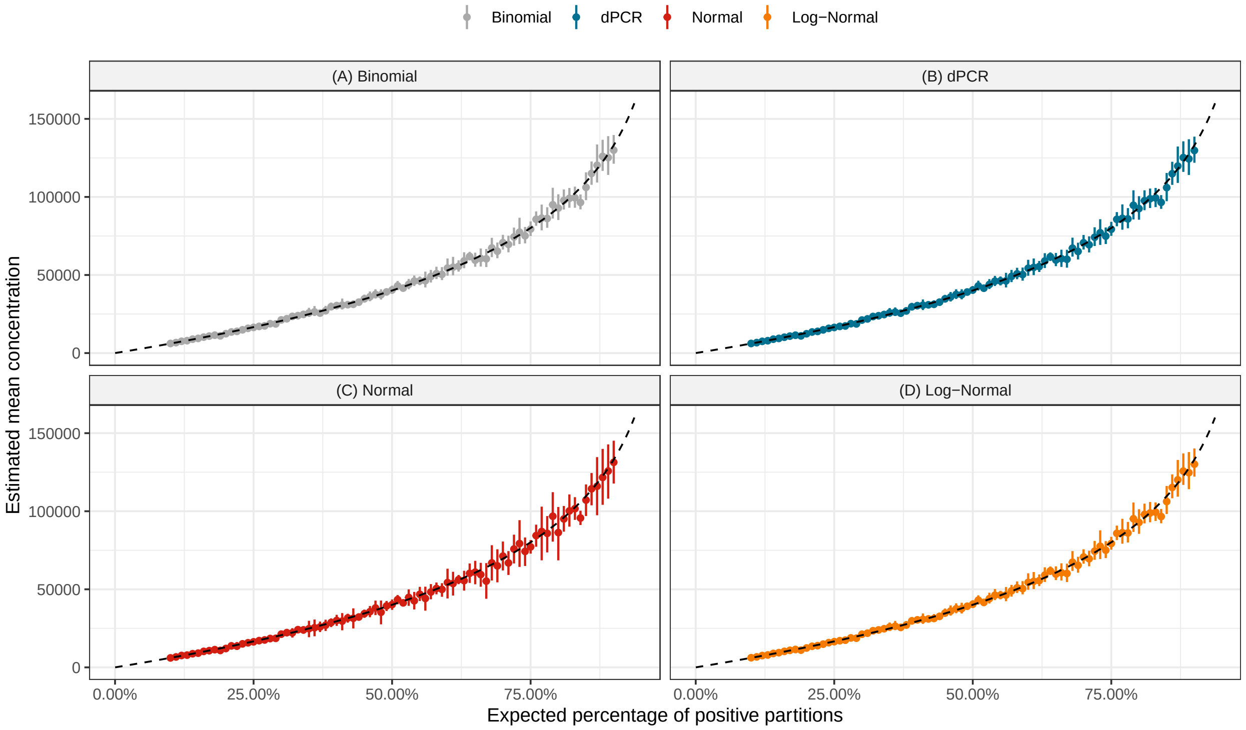

Fig S25. Estimation of a regression intercept from simulated dPCR measurements at high concentrations. Partition counts and measured concentrations from dPCR were simulated using log-linear regression models with a coefficient of 0.2 and different intercepts (corresponding to an increasing average share of positive partitions in the dPCR). For each simulated data set, the intercept (mean concentration across samples) was estimated using 10 simulated dPCR measurements, i. e. (A) with observed partition counts under a binomial model and (B–D) without partition counts (i. e. only reported concentrations) under a dPCR-specific, normal, and log-normal model. Shown are median estimates (dots) with 95% credible intervals (bars).

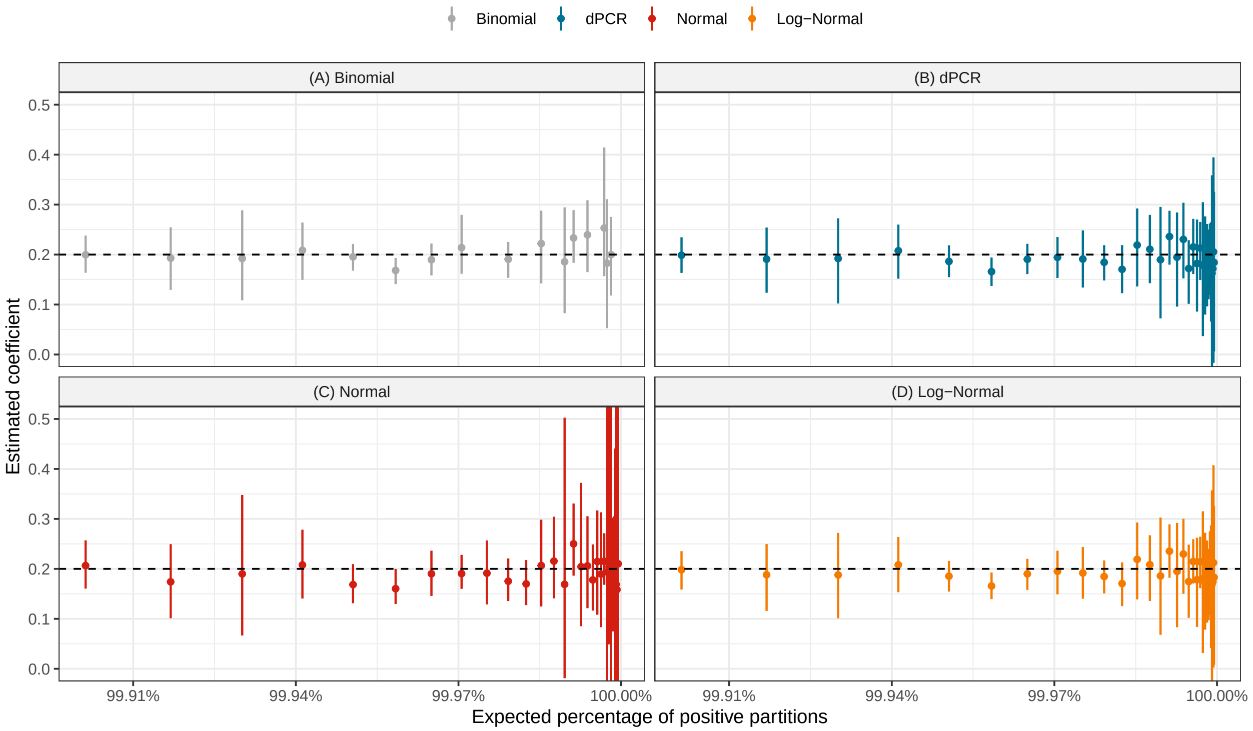

Fig S26. Estimation of a regression coefficient from simulated dPCR measurements at extreme concentrations. Partition counts and measured concentrations from dPCR were simulated using log-linear regression models with a coefficient of 0.2 and different intercepts (corresponding to an increasing average share of positive partitions in the dPCR). For each simulated data set, the slope coefficient was estimated using 10 simulated dPCR measurements, i. e. (A) with observed partition counts under a binomial model and (B–D) without partition counts (i. e. only reported concentrations) under a dPCR-specific, normal, and log-normal model. Shown are median estimates (dots) with 95% credible intervals (bars).

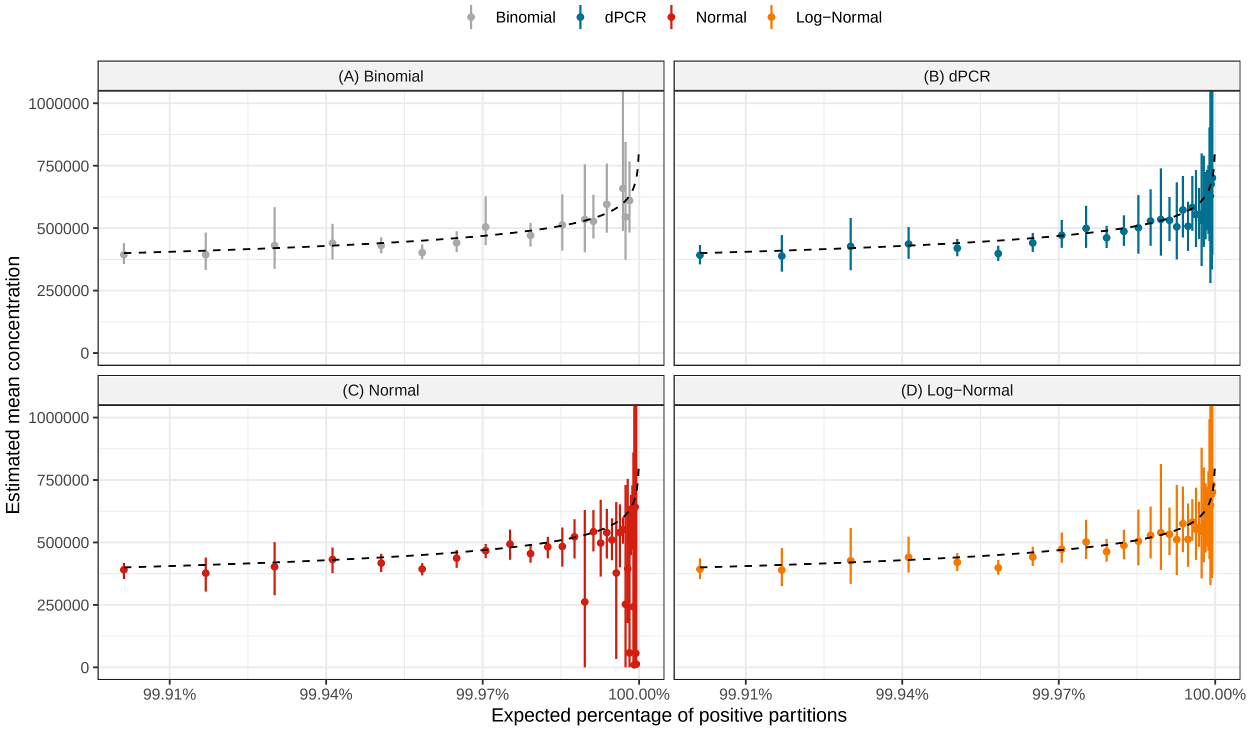

Fig S27. Estimation of a regression intercept from simulated dPCR measurements at extreme concentrations. Partition counts and measured concentrations from dPCR were simulated using log-linear regression models with a coefficient of 0.2 and different intercepts (corresponding to an increasing average share of positive partitions in the dPCR). For each simulated data set, the intercept (mean concentration across samples) was estimated using 10 simulated dPCR measurements, i. e. (A) with observed partition counts under a binomial model and (B–D) without partition counts (i. e. only reported concentrations) under a dPCR-specific, normal, and log-normal model. Shown are median estimates (dots) with 95% credible intervals (bars).

I Application to eDNA-based biomonitoring

I.1 Model

We modeled the expected concentration of free-eDNA of *Bugula neritina* t hours after removal of the organism as

$$\log(c_{t,i}) = a - bt + \gamma_i, \tag{41}$$

such that $\exp(a)$ is the concentration at $t = 0$ (intercept), b the exponential decay factor, and $\exp(\delta_i)$ a multiplicative fixed effect for the sample location in the aquarium (back left, back right, front left, front right, and middle). The half-life period can be computed as $\frac{\log(2)}{b}$.

I.2 Assay parameters

To fit our model with the dPCR-specific likelihood to the reported concentrations, we used broad priors for the assay parameters, as detailed in Table S2. Note that for the conversion factor κ, we here assumed $\kappa = v$ as the concentrations reported by Scriver et al. were in terms of the reaction concentration, not the original sample concentration. We furthermore fitted the model with a normal and log-normal likelihood, respectively. For the log-normal likelihood, we placed a half-normal prior with a standard deviation of 0.5 on the coefficient of variation. For the normal likelihood, we assumed a fixed variance, but parameterized it in terms of the average coefficient of variation to use the same noise prior as in the log-normal model. Finally, we also fitted a binomial model to the corresponding partition count data, which we back-calculated using information provided in the lab protocol in Scriver et al. [16]. For this, we assumed a scaling factor of $s = \frac{1}{21}$ for the reaction mix, and an average of 20 000 partitions with a volume of $v = 0.85\,\mathrm{nL}$ for the BioRad200^{TM} droplet generator. For zero measurements, we sampled the total number of partitions from a negative binomial distribution parameterized using the empirical mean and standard deviation of partition counts for the non-zero measurements. Again, we used a half-normal prior with a standard deviation of 0.5 for the remaining pre-PCR variation (ν_{pre}).

Parameter	Description	Prior	Details
$m_{\max}$	maximum number of partitions	$\text{Normal}^{+}(\mu = 30000, \sigma = 10000)$	accommodates dPCR systems with 10 000 to 50 000 partitions
$\delta_{\max}$	maximum relative partition loss	$\delta_{\max} = 0.5$	fixed to default of 50%
μ_{δ}	scaled logit-level expected relative partition loss	$\text{Normal}(\mu = -1.7, \sigma = 1.3)$	for $\delta_{\max} = 0.5$, this corresponds to an average loss of 1–30%
σ_{δ}	scaled logit-level standard deviation of partition loss	$\text{Normal}^{+}(\mu = 1.2, \sigma = 0.5)$	$\sigma_{\delta} \approx [0.5, 2]$
κ	concentration conversion factor	$\text{Normal}^{+}\left(\mu = 1 \times 10^{-3}, \sigma = 5 \times 10^{-2}\right)$	$\kappa = v \ll 1 \times 10^{-2}$
ν_{pre}	pre-PCR coefficient of variation	$\text{Normal}^{+}(\mu = 0, \sigma = 0.5)$	weakly informative prior, less than 100% variation

Table S2. Overview of priors for the dPCR-specific model of eDNA decay. Normal^{+} denotes a normal distribution truncated at zero.

J Application to wastewater-based epidemiology

To demonstrate inference from a time series of wastewater measurements, we used samples taken at the treatment plant of Zurich, Switzerland in the time period Sep 16, 2022 – Apr 30, 2025, extracted under the same protocol as described in Supplement E. During the 2022/23 winter season, pathogen concentrations were quantified from on average 5 samples per week using a fourplex dPCR assay with Influenza A (M gene), Influenza B (M gene), Respiratory Syncytial Virus (N gene), and SARS-CoV-2 (N2 gene) as targets. In the 2023/24 and 2024/25 seasons, a similar, sixplex dPCR assay with two additional targets (N1 gene of SARS-CoV-2, M gene of Murine Hepatitis Virus for extraction efficiency control) was used. In the 2024/25 season, sampling frequency was decreased to on average 4 samples per week.

For each winter season, we fitted the wastewater model implemented in the EpiSewer R package to the longitudinal measurements of Influenza A virus [17]. In this model, the effective reproduction number was smoothed using a random walk prior, and infections were assumed to arise from a stochastic renewal process with Poisson distributed noise. The full model specification is provided as reproducible code at `https://github.com/adrian-lison/dPCR-observation-model-study`.

J.1 Assay parameters

As a "gold standard" model, we used the observed number of positive and total partitions from the dPCR assay with a binomial likelihood and supplied the exact assay parameters to the model, i. e. a partition volume of $v = 0.519nL$, a sample volume of 40 mL, an elution of 80 µL, a dilution factor of 1:3, and a 25 µL reaction mix with 5 µL of the template and 20 µL of reagents. Overall, this yielded a conversion factor of 1.73×10^{-5}. Importantly, while there may be further factors influencing the relationship between the original concentration in the sample and the concentration in the PCR, e. g. recovery efficiency or inhibition, c should only include factors that were accounted for by the lab when reporting the sample concentration.

For the dPCR-specific model of measured wastewater concentrations, we assumed that the detailed assay parameters were unknown and used broad priors for the number of total partitions and the conversion factor (Table S3). Here we expected κ to be below 1×10^{-4}, but often much lower than that. We thus used a $\text{Normal}^{+}\left(\mu = 1 \times 10^{-5}, \sigma = 4 \times 10^{-5}\right)$ prior for κ, which gives a rather flat prior that has most of its probability mass below 1×10^{-4}.

Parameter	Description	Prior	Details
$m_{\max}$	maximum number of partitions	$\text{Normal}^{+}(\mu = 30000, \sigma = 10000)$	accommodates dPCR systems with 10 000 to 50 000 partitions
$\delta_{\max}$	maximum relative partition loss	$\delta_{\max} = 0.5$	fixed to default of 50%
μ_{δ}	scaled logit-level expected relative partition loss	$\text{Normal}(\mu = -1.7, \sigma = 1.3)$	for $\delta_{\max} = 0.5$, this corresponds to an average loss of 1–30%
σ_{δ}	scaled logit-level standard deviation of partition loss	$\text{Normal}^{+}(\mu = 1.2, \sigma = 0.5)$	$\sigma_{\delta} \approx [0.5, 2]$
κ	concentration conversion factor	$\text{Normal}^{+}\left(\mu = 1 \times 10^{-5}, \sigma = 4 \times 10^{-5}\right)$	$\kappa \ll 1 \times 10^{-4}$
ν_{pre}	pre-PCR coefficient of variation	$\text{Normal}^{+}(\mu = 0, \sigma = 1)$	weakly informative prior, less than 200% variation

Table S3. Overview of priors for the dPCR-specific model for wastewater concentrations. Normal^{+} denotes a normal distribution truncated at zero.

We also fitted the model with a normal and log-normal likelihood. For the log-normal likelihood, we placed a half-normal prior with a standard deviation of 1 on the coefficient of variation. For the normal likelihood, we assumed a fixed variance, but parameterized it in terms of the average

coefficient of variation to use the same noise prior as in the log-normal model.

Figure S28 shows prior and posterior distributions for the maximum number of partitions, average relative partition loss, concentration conversion factor, and pre-PCR coefficient of variation based on a model fitted to real-world measurements of Influenza A virus as presented in the main text.

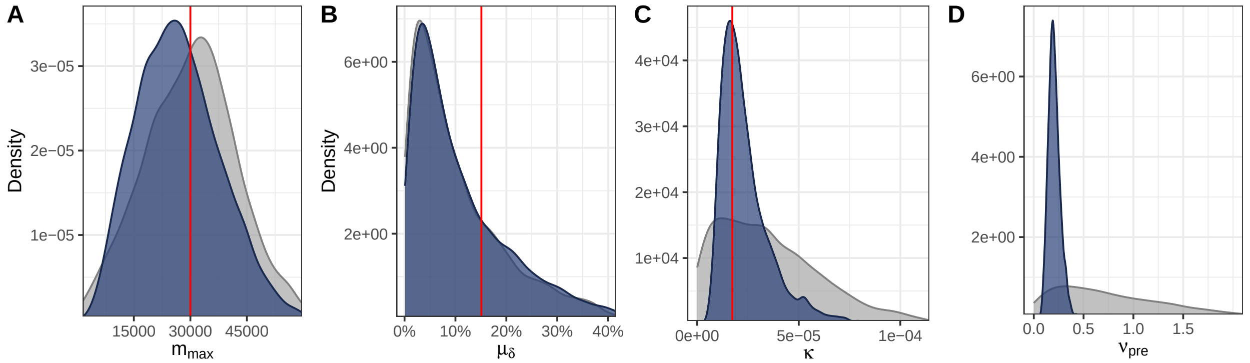

Fig S28. Inference of dPCR parameters and pre-PCR variation from a time series of dPCR measurements: Shown are prior and posterior distributions for (A) the maximum number of partitions $m_{\max}$, (B) the average relative partition loss μ_δ, (C) the conversion factor κ, and (D) the pre-PCR coefficient of variation ν_{pre} of a model applied to dPCR measurements of Influenza A virus concentrations at the municipal treatment plant of Zurich, Switzerland (Sep 16 – Dec 29, 2022). Vertical dashed lines show the true $m_{\max}$, μ_δ, and κ values based on the laboratory setup.

J.2 Additional results

In the following, we provide additional results of our model applied to wastewater data on different date ranges of measurements at the treatment plant of Zurich, Switzerland. As in the main text, we compared R_t estimates from a binomial model with partition count data to a model of concentration measurements using 1) the dPCR-specific likelihood, 2) a normal likelihood with constant variance, 3) a log-normal likelihood with constant coefficient of variation (Figures S29–S33).

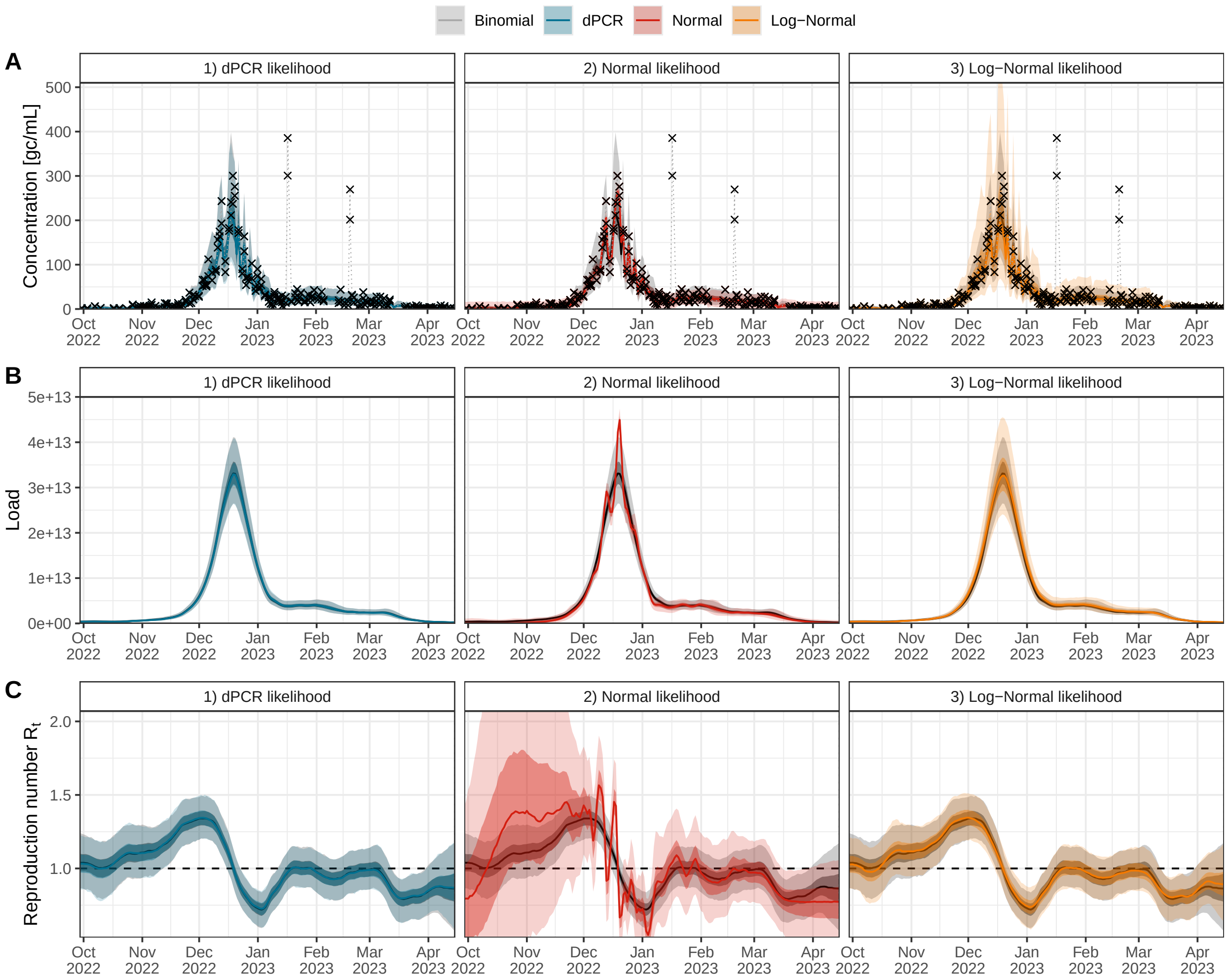

Fig S29. Modeling Influenza A virus concentrations at the municipal treatment plant of Zurich, Switzerland, Sep 16, 2022 – Apr 15, 2023: The epidemiological wastewater model (“EpiSewer”) was fitted to duplicate dPCR measurements of Influenza A virus concentrations at the municipal treatment plant of Zurich, Switzerland using 1) a dPCR-specific likelihood (blue), 2) a normal likelihood (red), and 3) a log-normal likelihood (orange) for concentration measurements. “Gold standard” estimates using a binomial likelihood for the partition counts and exact assay information are shown in grey. Each panel shows the median (lines) and 50% and 95% credible intervals (strong and weakly shaded areas) of A) posterior predictive distributions for dPCR measurements, B) estimated viral loads in wastewater over time, and C) the estimated effective reproduction number over time. Measured concentrations are shown in panel A (crosses).

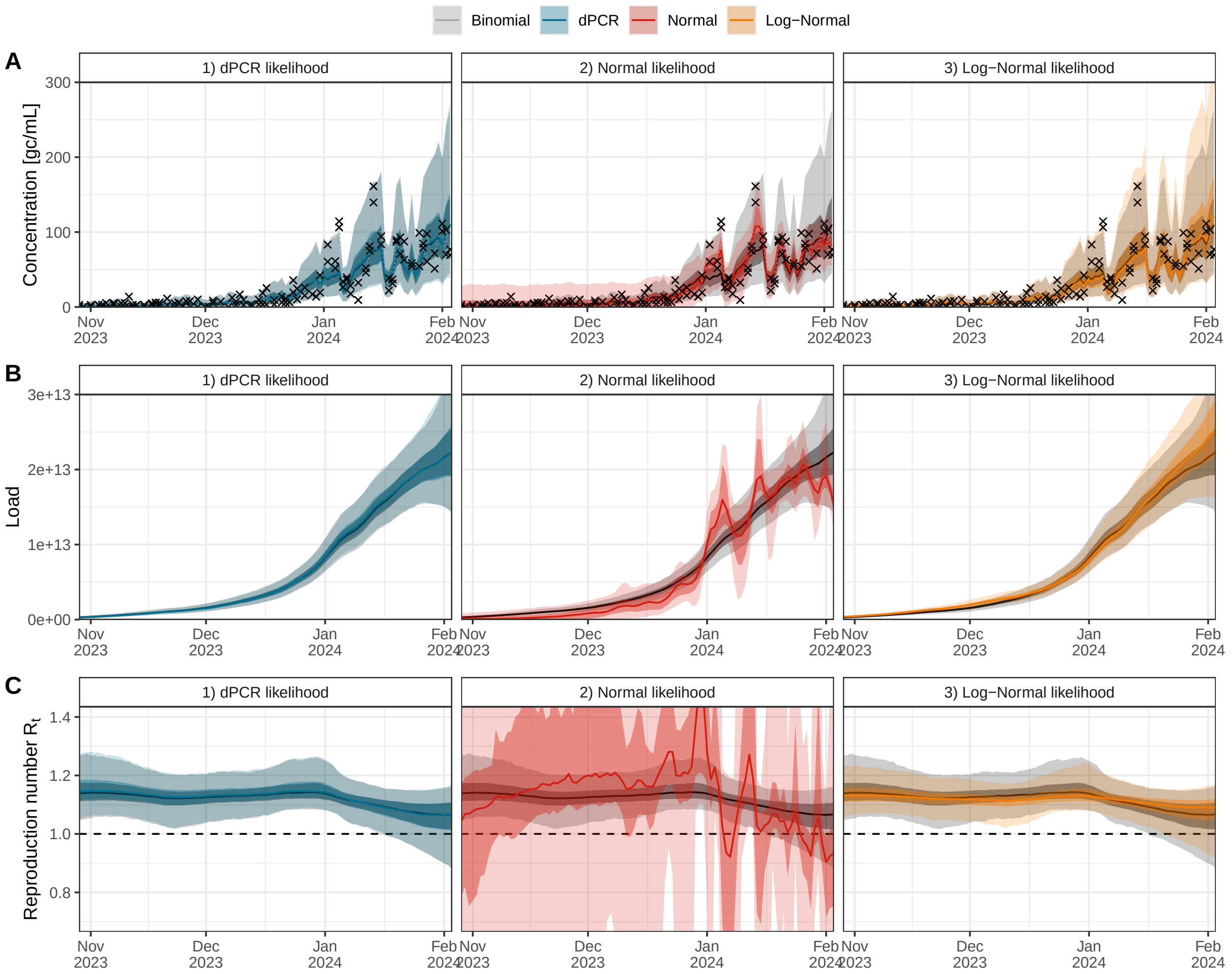

Fig S30. Modeling Influenza A virus concentrations at the municipal treatment plant of Zurich, Switzerland, Sep 15, 2023 – Feb 03, 2024: The epidemiological wastewater model (“EpiSewer”) was fitted to duplicate dPCR measurements of Influenza A virus concentrations at the municipal treatment plant of Zurich, Switzerland using 1) a dPCR-specific likelihood (blue), 2) a normal likelihood (red), and 3) a log-normal likelihood (orange) for concentration measurements. “Gold standard” estimates using a binomial likelihood for the partition counts and exact assay information are shown in grey. Each panel shows the median (lines) and 50% and 95% credible intervals (strong and weakly shaded areas) of A) posterior predictive distributions for dPCR measurements, B) estimated viral loads in wastewater over time, and C) the estimated effective reproduction number over time. Measured concentrations are shown in panel A (crosses).

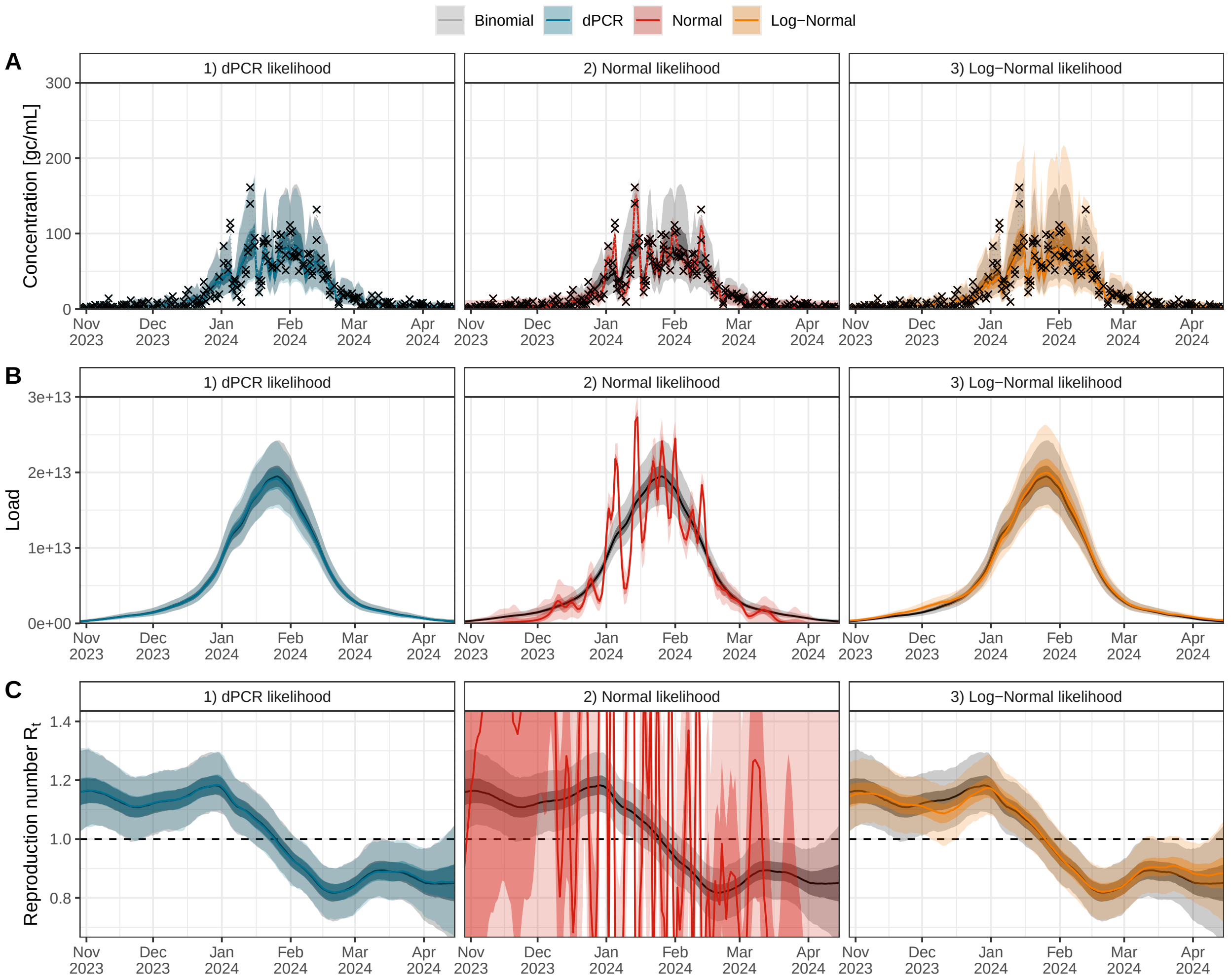

Fig S31. Modeling Influenza A virus concentrations at the municipal treatment plant of Zurich, Switzerland, Sep 15, 2023 – Apr 15, 2024: The epidemiological wastewater model ("EpiSewer") was fitted to duplicate dPCR measurements of Influenza A virus concentrations at the municipal treatment plant of Zurich, Switzerland using 1) a dPCR-specific likelihood (blue), 2) a normal likelihood (red), and 3) a log-normal likelihood (orange) for concentration measurements. "Gold standard" estimates using a binomial likelihood for the partition counts and exact assay information are shown in grey. Each panel shows the median (lines) and 50% and 95% credible intervals (strong and weakly shaded areas) of A) posterior predictive distributions for dPCR measurements, B) estimated viral loads in wastewater over time, and C) the estimated effective reproduction number over time. Measured concentrations are shown in panel A (crosses).

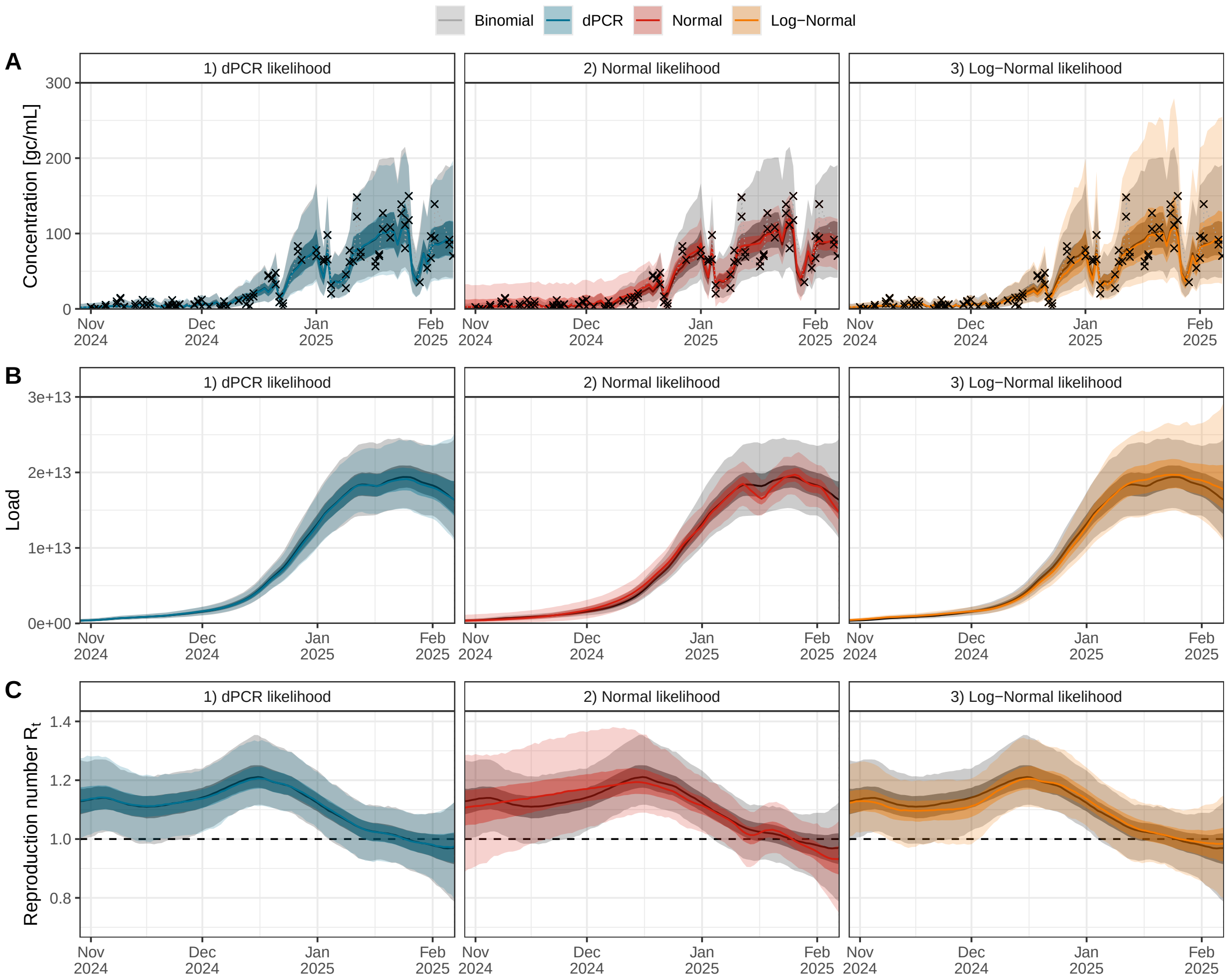

Fig S32. Modeling Influenza A virus concentrations at the municipal treatment plant of Zurich, Switzerland, Sep 15, 2024 – Feb 07, 2024: The epidemiological wastewater model ("EpiSewer") was fitted to duplicate dPCR measurements of Influenza A virus concentrations at the municipal treatment plant of Zurich, Switzerland using 1) a dPCR-specific likelihood (blue), 2) a normal likelihood (red), and 3) a log-normal likelihood (orange) for concentration measurements. "Gold standard" estimates using a binomial likelihood for the partition counts and exact assay information are shown in grey. Each panel shows the median (lines) and 50% and 95% credible intervals (strong and weakly shaded areas) of A) posterior predictive distributions for dPCR measurements, B) estimated viral loads in wastewater over time, and C) the estimated effective reproduction number over time. Measured concentrations are shown in panel A (crosses).

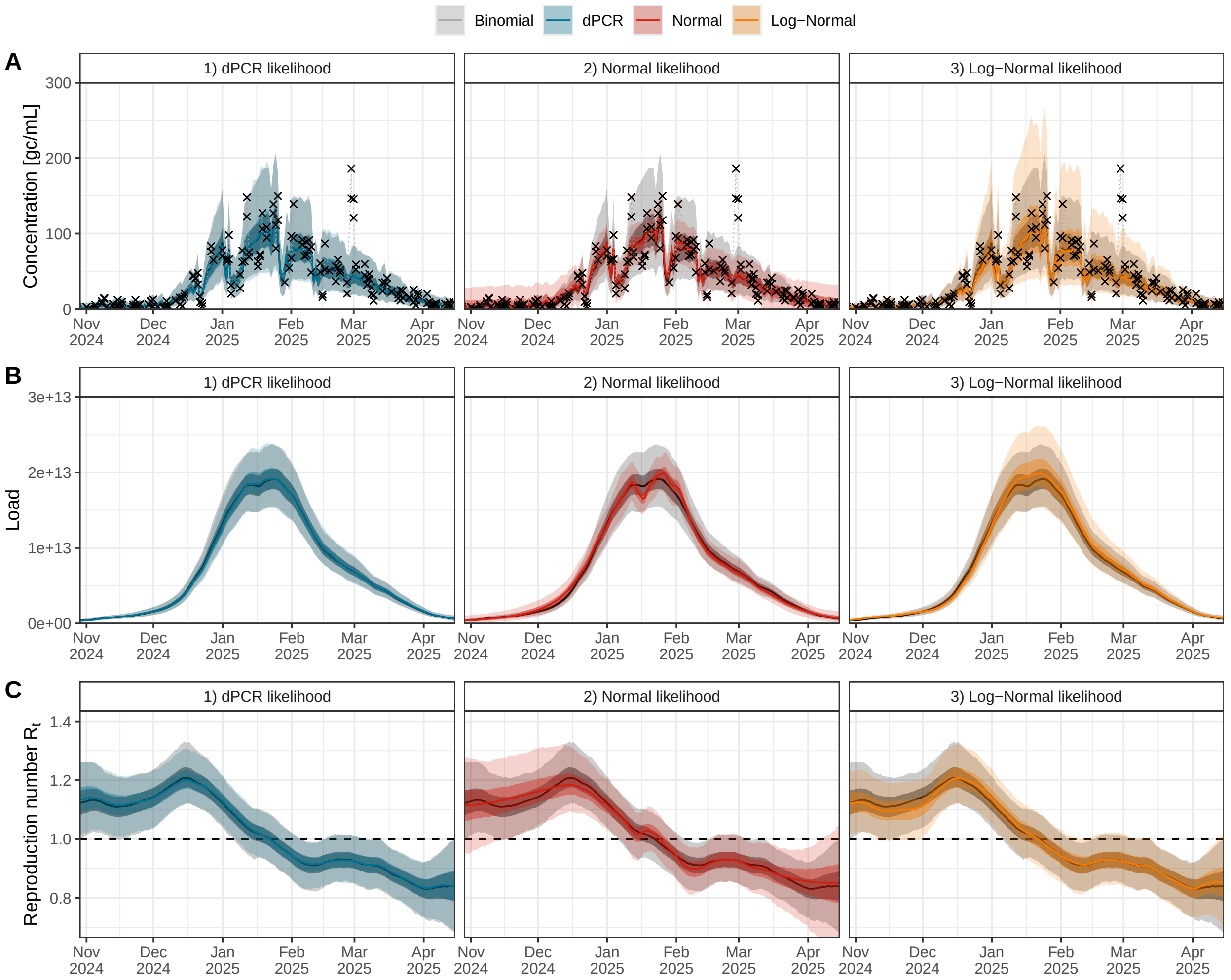

Fig S33. Modeling Influenza A virus concentrations at the municipal treatment plant of Zurich, Switzerland, Sep 15, 2024 – Apr 15, 2024: The epidemiological wastewater model ("EpiSewer") was fitted to duplicate dPCR measurements of Influenza A virus concentrations at the municipal treatment plant of Zurich, Switzerland using 1) a dPCR-specific likelihood (blue), 2) a normal likelihood (red), and 3) a log-normal likelihood (orange) for concentration measurements. "Gold standard" estimates using a binomial likelihood for the partition counts and exact assay information are shown in grey. Each panel shows the median (lines) and 50% and 95% credible intervals (strong and weakly shaded areas) of A) posterior predictive distributions for dPCR measurements, B) estimated viral loads in wastewater over time, and C) the estimated effective reproduction number over time. Measured concentrations are shown in panel A (crosses).

References

1. Dorazio RM, Hunter ME. Statistical Models for the Analysis and Design of Digital Polymerase Chain Reaction (dPCR) Experiments. Analytical Chemistry. 2015;87(21):10886–10893.

2. Dube S, Qin J, Ramakrishnan R. Mathematical Analysis of Copy Number Variation in a DNA Sample Using Digital PCR on a Nanofluidic Device. PLOS ONE. 2008;3(8):e2876.

3. Asmussen S, Jensen JL, Rojas-Nandayapa L. On the Laplace Transform of the Lognormal Distribution. Methodology and Computing in Applied Probability. 2016;18(2):441–458.

4. Corless RM, Gonnet GH, Hare DEG, Jeffrey DJ, Knuth DE. On the LambertW Function. Advances in Computational Mathematics. 1996;5(1):329–359.

5. Armbruster DA, Pry T. Limit of Blank, Limit of Detection and Limit of Quantitation. The Clinical Biochemist Reviews. 2008;29(Suppl 1):S49–S52.

6. Huisman JS, Scire J, Caduff L, Fernandez CX, Ganesanandamoorthy P, Kull A, et al. Wastewater-Based Estimation of the Effective Reproductive Number of SARS-CoV-2. Environmental Health Perspectives. 2022;130(5):057011.

7. Nadeau S, Devaux AJ, Bagutti C, Alt M, Hampe EI, Kraus M, et al. Influenza transmission dynamics quantified from RNA in wastewater in Switzerland. Swiss Medical Weekly. 2024;154(1):3503–3503.

8. Cureton EE. Unbiased Estimation of the Standard Deviation. The American Statistician. 1968;22(1):22–22.

9. Cleveland WS, Loader C. Smoothing by Local Regression: Principles and Methods. In: Härdle W, Schimek MG, editors. Statistical Theory and Computational Aspects of Smoothing. Heidelberg: Physica-Verlag HD; 1996. p. 10–49.

10. Stan development team. Stan Modeling Language Users Guide and Reference Manual, Version 2.35; 2024.

11. Gabry J, Češnovar R. Cmdstanr: R Interface to 'CmdStan' [Manual]; 2024.

12. Geyer C. Introduction to Markov Chain Monte Carlo. In: Brooks S, Gelman A, Jones G, Meng XL, editors. Handbook of Markov Chain Monte Carlo. Chapman and Hall/CRC; 2011. p. 3–48.

13. Betancourt M. A Conceptual Introduction to Hamiltonian Monte Carlo. arXiv; 2018.

14. Betancourt M. Diagnosing Suboptimal Cotangent Disintegrations in Hamiltonian Monte Carlo. arXiv; 2016.

15. Gelman A, Rubin DB, et al. Inference from Iterative Simulation Using Multiple Sequences. Statistical Science. 1992;7(4):457–472.

16. Scriver M, von Ammon U, Youngbull C, Pochon X, Stanton JAL, Gemmell NJ, et al. Drop It All: Extraction-Free Detection of Targeted Marine Species through Optimized Direct Droplet Digital PCR. PeerJ. 2024;12:e16969.

17. Lison A. EpiSewer: Estimate Epidemiological Parameters from Wastewater Measurements. Zenodo. 2024; Available from: https://zenodo.org/doi/10.5281/zenodo.13899759.
